# Two distributed enhancers of *Sox17* additively regulate the developmental fate of bipotential ventral pancreato-biliary progenitor cells

**DOI:** 10.1242/dev.205751

**Published:** 2026-09-18

**Authors:** Ryan R. Finnel, Jessica R. Musselman, Margaret E. McFadden, Isabella M. Silvestri, Jennifer Skelton, Leesa L. Sampson, Anna B. Osipovich, Christopher V. E. Wright, Mark A. Magnuson

**Affiliations:** ^1^ Center for Stem Cell Biology, Vanderbilt University, Nashville, TN 37232, USA; ^2^ Department of Cell and Developmental Biology, Vanderbilt University, Nashville, TN 37232, USA; ^3^ Program in Developmental Biology, Vanderbilt University, Nashville, TN 37232, USA; ^4^ School of Engineering, Vanderbilt University, Nashville, TN 37232, USA; ^5^ Department of Molecular Physiology and Biophysics, Vanderbilt University, Nashville, TN 37232, USA

**Keywords:** *Sox17*, Endoderm, Enhancers, *Cis*-regulatory elements, Gallbladder, Ventral pancreas, Mouse

## Abstract

SRY-box transcription factor 17 (*Sox17*) is essential for hepato-pancreato-biliary development. We identified two distributed enhancers of *Sox17*, Sox17e-13 and Sox17e-231, within the likely murine Sox17 topologically associating domain. Individual CRISPR-mediated enhancer deletions temporally reduce endodermal *Sox17* expression, delaying the transition of bipotent ventral pancreato-biliary progenitor cells to a lineage-unipotent ventral pancreatic fate, reducing pancreas size and increasing gallbladder size. Deleting both enhancers further reduces *Sox17* expression and delays conversion of bipotent progenitor cells to ventral pancreatic or biliary fates, reducing both pancreas and gallbladder size. Crossing each enhancer mutation to a *Sox17* null allele revealed differential and profound sensitivities of the extrahepatic biliary system to alterations of *Sox17* expression, as demonstrated by gallbladder hypoplasia, gallbladder agenesis with a cystic duct, and biliary atresia. These studies indicate that Sox17e-13 and Sox17e-231 additively modulate *Sox17* expression, that the differentiation of bipotential ventral pancreato-biliary cells to unipotent fates is *Sox17* dosage dependent, and that thresholds for *Sox17* action during pancreato-biliary formation vary by developmental stage, further defining the role of *Sox17* in hepato-pancreato-biliary formation and function.

## Teaser

**Summary:** Two enhancers of *Sox17* regulate the conversion of bipotential ventral pancreato-biliary progenitor cells towards lineage unipotency in an additive and temporal manner.

## INTRODUCTION

During development, critical cell-state transitions are precisely regulated to ensure accurate and robust tissue development (Luo et al., 2023). Transcriptional enhancers govern such transitions by ensuring accurate spatial, temporal and quantitative expression of key transcriptional regulators (Long et al., 2016). Mutations within critical enhancer regions cause developmental abnormalities, contribute to polygenic disease susceptibility, and affect many quantitative traits (Zaugg et al., 2022; Jindal et al., 2023; Lim et al., 2024). Enhancers typically contain multiple *cis*-regulatory elements (CREs) for which identity, spacing and orientation determine overall enhancer behavior (Jindal and Farley, 2021). In addition, enhancers likely contribute to transcription factor (TF) dosage effects (Naqvi et al., 2023, 2025), which regulate the tempo of critical cell state transitions (Tobias et al., 2025) and control the allocation of stem and progenitor cells to specific lineages (Goolam et al., 2016).

Robust regulation of gene expression is often achieved by the presence of multiple distributed enhancers, also known as shadow enhancers, which have overlapping tissue and temporal activity that contribute to the expression of a common target gene (Barolo, 2012). Distributed enhancers, which can vary extensively in their distance from the regulated gene and exhibit different interaction modes (Long et al., 2016), may serve to buffer against genetic and environmental perturbations during critical developmental phases (Hong et al., 2008; Frankel et al., 2010; Perry et al., 2010; Osterwalder et al., 2018). Multiple enhancers with similar TF binding profiles may locally concentrate key TFs into transcriptional condensates that establish a permissive transcriptional microenvironment (Sabari et al., 2018; Tsai et al., 2019).

SRY-box (Sox) TFs regulate many critical cell-fate transitions during development (Kamachi and Kondoh, 2013; Goolam et al., 2016; Gonen et al., 2018; Abatti et al., 2023). *Sox17*, a member of the SoxF family, is essential for the formation of endodermal and hemogenic endothelial cells (Kanai-Azuma et al., 2002; Engert et al., 2009; Liao et al., 2009; Spence et al., 2009). Previously, we identified two promoters within *Sox17* that produce endodermal and endothelial lineage-specific RNAs that encode the same protein (Choi et al., 2012; Trinh et al., 2022, 2023). More recently, we determined that CREs near the *Sox17* transcription start site (TSS) 2, which include a Sox17 binding motif, regulate endodermal *Sox17* expression (Trinh et al., 2025), revealing the importance of *Sox17* gene dosage for gallbladder and extrahepatic biliary duct (EHBD) development.

Multiple cell-fate lineage-diversification decisions occur between embryonic (E) day 8.5 and E11.5 in the mouse that are involved in the formation of the hepato-pancreato-biliary (HPB) system (Spence et al., 2009; Villasenor and Stainier, 2017). One such lineage-determining transition is the conversion of bipotential ventral pancreato-biliary progenitor cells, which express both *Sox17* and *Pdx1*, to unipotent lineage fates around E9.5. At this time, cells that assume a biliary fate continue to express *Sox17* and cease expressing *Pdx1*, a key regulator of the pancreatic cell fate, whereas cells that assume a ventral pancreatic cell fate cease *Sox17* expression but continue to express *Pdx1* (Spence et al., 2009; Uemura et al., 2010). Indeed, as *Sox17* is expressed within ventral pancreato-biliary progenitor cells but not in dorsal pancreatic progenitors, the gene may contribute to the differential regulation of the many transcriptional and morphogenic events that occur during ventral and dorsal pancreatic organ bud differentiation (Pan and Wright, 2011; Li et al., 2018).

To better understand how *Sox17* is regulated during HPB development, and to determine how specific distal enhancers contribute to this process, we identified two widely separated endodermal enhancers of *Sox17*, Sox17e-13 and Sox17e-231, and used CRISPR mutagenesis to derive mice with single or dual enhancer deletions, allowing us to determine the individual and combinatorial effects of enhancer deletions during HPB formation. Analysis of embryos and adult mice revealed that the two enhancers function in an additive and temporally dependent manner to regulate *Sox17* expression in bipotent ventral pancreato-biliary cells, thereby playing a crucial role in the formation of both the ventral pancreas and EHBD system.

## RESULTS

### Cross-species conservation of the *SOX17/Sox17* gene locus

Topologically associating domains (TADs) bring linearly distant DNA sequences into close spatial proximity to create discrete regulatory domains (Dixon et al., 2012; Nora et al., 2012). Consistent with key developmental TFs often being the only gene within a TAD (Dowen et al., 2014; Wu et al., 2021), in humans *SOX17* is the only protein-coding gene within a ∼336 kb TAD bounded by two tandem-oriented CTCF motifs (Rhodes et al., 2019; Wu et al., 2021; Landshammer et al., 2023). Mice contain two similarly spaced, highly conserved, tandem-oriented CTCF sites corresponding to the human *SOX17* TAD-defining CTCF sites, suggesting the mouse *Sox17* TAD is similar in size to that in humans (∼352 kb versus ∼336 kb, respectively) (Fig. S1A). Moreover, *Sox17* is the only protein-coding gene within the putative mouse TAD. These findings suggest significant organizational conservation of the *SOX17/Sox17* locus during mammalian evolution (Fig. 1A, Fig. S1B).

**Fig. 1.**
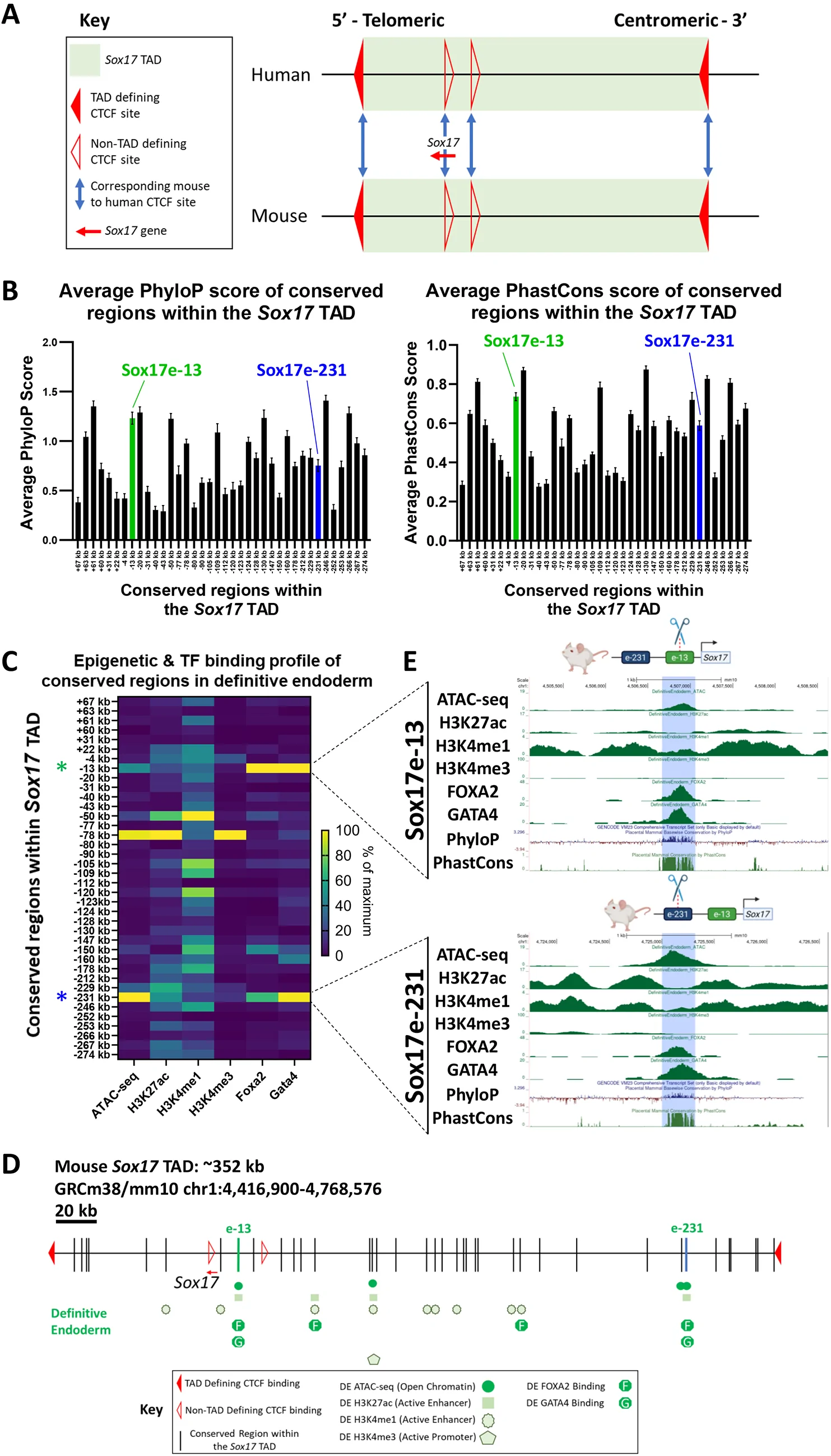
**SOX17/*Sox17* gene locus conservation and identification of two enhancer regions.** (A) Schematic of conserved CTCF binding sites between mouse and human genomes (based on comparative mapping in Fig. S1A). (B) Conservation scores of conserved regions identified within the likely *Sox17* TAD using PhyloP (left) and PhastCons (right). Data presented as mean±s.e.m.; *n* values vary depending on the length of the conserved region. (C) Signal intensity heatmap of open chromatin, epigenetic marks and endoderm-specific TF binding at each conserved region within the *Sox17* TAD. Signal intensity is quantified as a percentage of maximum intensity within each column. Asterisks denote Sox17e-13 (green) and Sox17e-231 (blue). (D) *Sox17* TAD schematic incorporating CTCF binding sites, locations of conserved regions, and the presence of epigenetic or TF binding at these sites. Symbols were placed when a signal reached 25% of the maximum signal, except for H3K4me1, which was placed when the signal reached 50% of the maximum signal. (E) Insets showing the epigenetic, transcription factor binding, and conservation peaks within each enhancer. The blue highlight is over each corresponding enhancer. Viewing window for Sox17e-13: mm10 chr1:4505106-4508624. Viewing window for Sox17e-231: mm10 chr1:4725005-4725319. Near each inset is a schematic of the enhancer deletion alleles generated by this study. Sox17e-13, mm10 chr1:4506670-4507060; Sox17e-231, mm10 chr1:4725005-4725319.

To identify conserved regions within the putative mouse *Sox17* TAD, possibly revealing enhancers and other important regulatory sites, we developed four criteria based on region size and cross-species conservation scores using the PhyloP and PhastCons algorithms within the UCSC genome browser, as detailed in the Materials and Methods (Siepel et al., 2005; Pollard et al., 2010; Perez et al., 2025). This approach successfully identified mouse and human enhancers previously established in the literature, validating the method (Fig. S1C) (Fujitani et al., 2006; Gonen et al., 2018; Liu et al., 2022; Luo et al., 2023; Kaplan et al., 2024). We then applied our approach to the *Sox17* TAD and identified 38 conserved regions of at least 100 bp (Fig. 1B, Table S1).

To determine whether any of the 38 identified conserved regions play a role in regulating *Sox17* endodermal expression, we assessed chromatin accessibility, permissive histone modifications, and binding of endoderm-enriched TFs to these regions in mouse definitive endoderm using previously published ATAC-seq and ChIP-seq datasets (Cernilogar et al., 2019) (Fig. 1C, Fig. S1D, Table S1). We then created a graphical model of the mouse *Sox17* TAD showing the epigenetic features and TF binding profiles for each of the 38 conserved regions (Fig. 1D). Inspection of the model revealed two potential enhancer regions of interest, Sox17e-13 and Sox17e-231, that are located −13 kb and −231 kb upstream of *Sox17* TSS2. The two regions were 391 and 315 bp in length, respectively, and both displayed open chromatin, permissive histone marks, and co-binding of FOXA2 (a pioneer factor) and GATA4 (stabilizes FOXA2-mediated chromatin opening) (Fig. 1D,E) (Cernilogar et al., 2019). Prior studies using mouse and human embryonic stem cells (ESCs) have implied a functional role for both regions as *Sox17* enhancers in endodermal and pancreatic development. Specifically, the mouse Sox17e-13 region showed interaction with the *Sox17* TSS2 promoter in a 3C assay of definitive endoderm (Trinh et al., 2022). CRISPRi-based screens in human embryonic stem cells revealed that the orthologous Sox17e-13 and Sox17e-231 regions contribute to *SOX17* expression during definitive endoderm differentiation (Luo et al., 2023), and Sox17e-231 contributes to pancreatic differentiation (Kaplan et al., 2024). Given these prior findings and our re-identification of both regions using a metadata strategy, we used CRISPR mutagenesis to derive mice containing either single or dual enhancer deletions, allowing us to precisely assess the individual and combined roles of Sox17e-13 and Sox17e-231 during mouse development (Fig. 1E).

### Sox17e-13 and Sox17e-231 additively regulate *Sox17* expression

We first assessed the homozygous viability of single- and dual-deletion mutations in weaning-age mice and observed no associated lethality for any of the three alleles (Table S2). To determine whether the deleted regions might have altered *Sox17* gene expression during HPB formation, we measured the *Sox17* endoderm-enriched TSS2-derived short mRNA in embryos (Fig. 2A), focusing on a 4-day interval (E8.5 to E11.5). During this period, two critical cell-state transitions occur: multipotent HPB progenitor cells differentiate into hepatic and bipotent ventral pancreato-biliary progenitors, and bipotent ventral pancreato-biliary progenitors give rise to the ventral pancreatic and biliary lineages (Spence et al., 2009; Uemura et al., 2010). No differences in *Sox17* short mRNA expression were observed in *Sox17*^Δ*e-13/*Δ^*^e-13^* or *Sox17*^Δ*e-231/*Δ^*^e-231^* embryos at E8.5, but reduced expression was observed at E9.5 and E10.5 before returning to wild-type expression levels at E11.5 (Fig. 2B,C). In contrast, *Sox17*^Δ*e-13&e-231/*Δ^*^e-13&e-231^* embryos showed no change at E8.5, decreases at both E9.5 and E10.5, and a failure of *Sox17* expression to return to normal at E11.5 (Fig. 2D). No changes were observed in endothelium-specific TSS1-derived *Sox17* long mRNA expression (Fig. 2A-D), nor in endothelium or liver markers across mutant lines (Fig. S2). These data indicate that deletion of the two genomic regions, individually or in combination, exerts measurable effects on endodermal *Sox17* mRNA expression during a narrow time window that coincides with a major period of ventral pancreato-biliary specification and lineage separation.

**Fig. 2.**
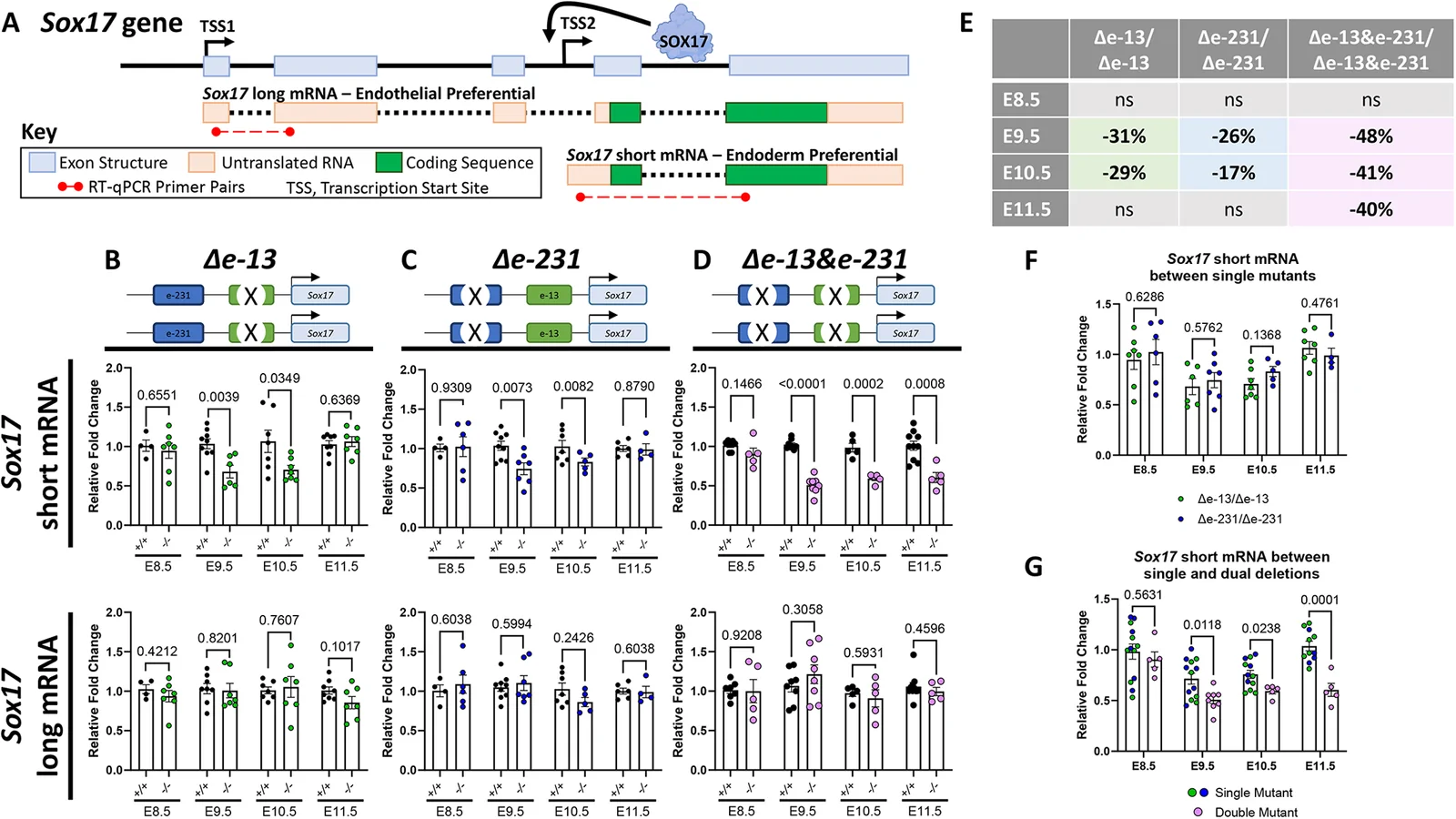
**Sox17e-13 and Sox17e-231 additively regulate *Sox17* expression.** (A) Schematic of the mouse *Sox17* gene showing DNA exon structure with the two alternate promoters and positive autoregulation at the TSS2 promoter, the two mRNA forms derived from each alternate promoter, and the RT-qPCR primer strategy for each mRNA form. (B-D) Whole-embryo RT-qPCR of *Sox17* short and long mRNA expression from E8.5 to E11.5 in enhancer mutant and wild-type embryos for: (B) *Sox17*^Δ*e-13*^: E8.5 +/+ *n*=4, −/− *n*=7; E9.5 +/+ *n*=9, −/− *n*=6; E10.5 +/+ *n*=7, −/− *n*=7; E11.5 +/+ *n*=8, −/− *n*=7; (C) *Sox17*^Δ*e-231*^: E8.5 +/+ *n*=4, −/− *n*=6; E9.5 +/+ *n*=9, −/− *n*=7; E10.5 +/+ *n*=7, −/− *n*=5; E11.5 +/+ *n*=6, −/− *n*=4; (D) *Sox17*^Δ*e-13&e-231*^: E8.5 +/+ *n*=7, −/− *n*=5; E9.5 +/+ *n*=8, −/− *n*=8; E10.5 +/+ *n*=5, −/− *n*=5; E11.5 +/+ *n*=10, −/− *n*=5. Each dot is one biological replicate. Each biological replicate is averaged from three technical replicates. (E) Table displaying the percentage change in *Sox17* short mRNA expression in mutant embryos in comparison to wild-type embryos from E8.5-E11.5 time points (derived from the data shown in B-D). ns, non-significant. (F) Comparison of short *Sox17* mRNA expression levels between Sox17e-13 and Sox17e-231 single enhancer mutants at E8.5-E11.5 (derived from the data shown in B-D). (G) Comparison of short *Sox17* mRNA expression levels between pooled *Sox17*^Δ*e-13/*Δ*e-13*^ and *Sox17*^Δ*e-231/*Δ*e-231*^ single mutants to *Sox17*^Δ*e-13&e-231/*Δ*e-13&e-231*^ deletion mutants at E8.5-E11.5 (derived from the data shown in B-D). Data presented as mean±s.e.m.; significance assessed by unpaired *t*-test, all *P*-values displayed.

*Sox17* short mRNA levels at E9.5 showed a ∼31% decrease in *Sox17*^Δ*e-13/*Δ^*^e-13^*, a ∼25% decrease in *Sox17*^Δ*e-231/*Δ^*^e-231^* and a ∼48% decrease in *Sox17*^Δ*e-13&e-231/*Δ^*^e-13&e-231^* (Fig. 2E). The similar reduction of *Sox17* short mRNA between the single mutants suggests similar functional potency of the Sox17e-13 and Sox17e-231 regions (Fig. 2F). The impairment of *Sox17* short mRNA expression between E9.5 and E10.5 in the dual-deletion mutant was approximately double that of the single-deletion mutants (Fig. 2G), strongly suggesting that the two regions act as enhancers that additively stimulate *Sox17* expression during this time window.

### Sox17e-13 and Sox17e-231 temporally regulate the binary cell-fate decision of ventral pancreato-biliary progenitor cells

*Sox17* is required for the specification of bipotential ventral pancreato-biliary progenitors, denoting an early positive role for both ventral pancreas and gallbladder development. When the binary ventral-pancreas versus gallbladder fate decision occurs between E9.5 and E10.5, *Sox17* becomes restricted to biliary progenitors and shifts solely to a positive role in gallbladder development (Spence et al., 2009; Uemura et al., 2010). Since the reduction in *Sox17* short mRNA expression at E9.5 and E10.5 coincides temporally with this binary fate decision, and because our prior studies showed a role for TSS2-driven *Sox17* expression in gallbladder and EHBD formation, we next performed immunofluorescence staining of embryos at E9.5 and E10.5 to determine how the enhancer deletions affected the progression of biliary versus pancreas development and lineage separation.

In wild-type embryos at E9.5, both the biliary and ventral pancreatic buds were clearly demarcated with bipotent SOX17^+^/PDX1^+^ cells present in the ventral pancreatic and biliary buds (Fig. 3A). In marked contrast, *Sox17*^Δ*e-13/*Δ^*^e-13^* and *Sox17*^Δ*e-231/*Δ^*^e-231^* embryos exhibited altered biliary and ventral pancreatic bud morphology, an extended zone of bipotent progenitor cells, and decreased numbers of ventral pancreatic progenitor cells. *Sox17*^Δ*e-13&e-231/*Δ^*^e-13&e-231^* dual-deletion embryos had a severely diminished and spatially disorganized ventral pancreato-biliary bud, with fewer ventral pancreatic and biliary progenitor cells, as well as increased numbers of bipotential progenitor cells (Fig. 3A,C). Consistent with *Sox17* being required to establish and/or maintain distinct spatial boundaries between the ventral pancreas, gallbladder and liver (Spence et al., 2009), one E9.5 *Sox17*^Δ*e-13&e-231/*Δ^*^e-13&e-231^* embryo exhibited HNF4α^+^ liver progenitor cells that were intermingled with other cells in the ventral pancreato-biliary bud (Fig. S3A).

**Fig. 3.**
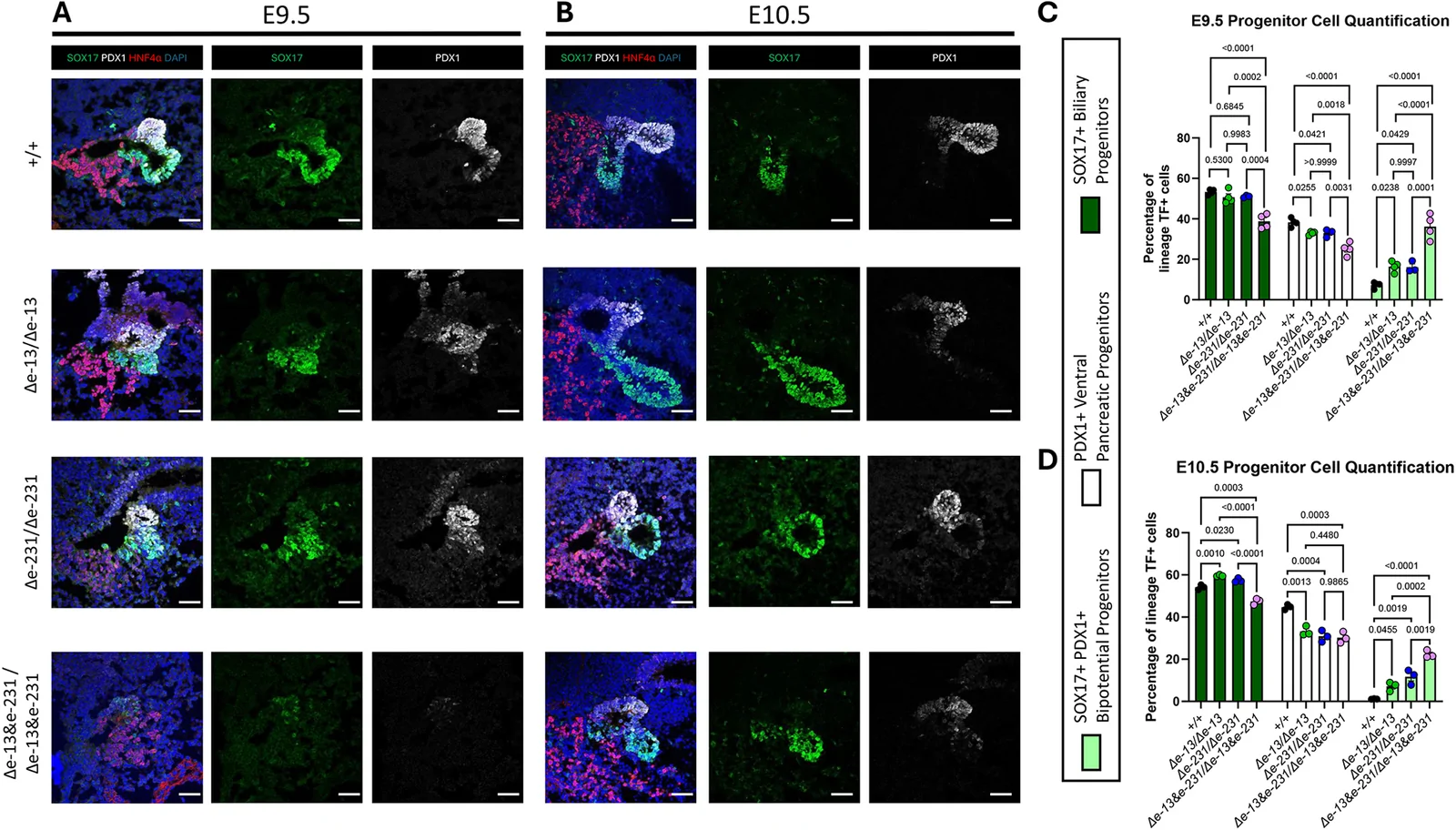
**Sox17e-13 and Sox17e-231 deletions delay the pancreato-biliary cell state transition.** (A) E9.5 immunofluorescence staining of enhancer mutants for SOX17 (green; biliary), PDX1 (white; pancreas), HNF4α (red; liver) and DAPI (blue; nucleus). Scale bars: 50 μm. (B) E10.5 immunofluorescent staining of enhancer mutants using the same scheme as A. Scale bars: 50 μm. (C) Quantification of E9.5 progenitor populations. Dark green bars, SOX17^+^ biliary progenitors; white bars, PDX1^+^ ventral pancreatic progenitors; light green bars, SOX17^+^ PDX1^+^ bipotential ventral-pancreato biliary progenitors. *Sox17^+/+^ n*=4; *Sox17*^Δ*e-13/*Δ*e-13*^ *n*=4; *Sox17*^Δ*e-231/*Δ*e-231*^ *n*=3; *Sox17*^Δ*e-13&e-231/*Δ*e-13&e-231*^ *n*=4. (D) Quantification of E10.5 progenitor populations. Graph uses same color scheme as C. *Sox17^+/+^ n*=3; *Sox17*^Δ*e-13/*Δ*e-13*^ *n*=3; *Sox17*^Δ*e-231/*Δ*e-231*^ *n*=3; *Sox17*^Δ*e-13&e-231/*Δ*e-13&e-231*^ *n*=3. Each dot is one biological replicate. Data presented as mean±s.e.m.; significance assessed by one-way ANOVA; all *P*-values shown.

While wild-type embryos at E10.5 showed the expected separated biliary and ventral pancreatic bud morphologies with few remaining bipotential progenitors (Fig. 3B), both *Sox17*^Δ*e-13/*Δ^*^e-13^* and *Sox17*^Δ*e-231/*Δ^*^e-231^* embryos exhibited impaired ventral pancreatic and biliary bud morphologies indicated by an increased number of bipotent SOX17^+^/PDX1^+^ progenitor cells, increased number of SOX17^+^ biliary progenitor cells, and fewer PDX1^+^ ventral pancreatic progenitor cells (Fig. 3B,D). These results suggest a delayed transition of bipotential progenitor cells towards the binary fate-separation decision and a rebalancing of progenitors culminating in fewer ventral pancreatic progenitors and increased biliary progenitors. In contrast to the single-enhancer deletions, the *Sox17*^Δ*e-13&e-231/*Δ^*^e-13&e-231^* dual-deletion embryos had reduced numbers of biliary and ventral pancreatic progenitor cells compared to wild type, as well as notably reduced immunofluorescence signals for SOX17 and PDX1 (Fig. 3B,D). While the percentage of ventral pancreatic progenitors in the *Sox17*^Δ*e-13&e-231/*Δ^*^e-13&e-231^* embryos was similar to single-deletion *Sox17*^Δ*e-13/*Δ^*^e-13^* and *Sox17*^Δ*e-231/*Δ^*^e-231^* embryos, a larger fraction of cells exhibited SOX17/PDX1 co-immunofluorescence, suggesting an even further delay in the transition of bipotential progenitors toward selecting either ventral pancreas or biliary fate (Fig. 3D). Immunofluorescence staining at E10.5 to address proliferation and apoptosis showed no obvious differences, supporting the idea that the greater number of bipotent progenitors arise from a delay in progression towards unipotent fates (Fig. S3B).

As the cell-state transition towards lineage unipotency is further delayed but not eliminated when both enhancers are deleted, we conclude that Sox17e-13 and Sox17e-231 are not directly required for the ventral pancreato-biliary cell-state transition. Instead, they may ensure that *Sox17* expression exceeds the threshold necessary to maintain the developmental tempo for proper lineage allocation during a 2-day period when critical cell fate decisions are occurring.

### Sox17e-13 and Sox17e-231 independently promote ventral pancreas development

To further investigate how deleting both Sox17e-13 and Sox17e-231 affects pancreatic formation, we measured the expression of *Pdx1* and *Ptf1a*, two essential regulators of the pancreas fate (Pan and Wright, 2011). Because their ability to capture pancreatic fate varies by timing (Offield et al., 1996; Duque et al., 2022), *Pdx1* was analyzed from E8.5 to E10.5 and *Ptf1a* from E9.5 to E11.5. *Pdx1* expression in single-deletion *Sox17*^Δ*e-13/*Δ^*^e-13^* and *Sox17*^Δ*e-231/*Δ^*^e-231^* embryos was unchanged at E8.5 and then downregulated at E9.5 and E10.5. In contrast, in dual-deletion *Sox17*^Δ*e-13&e-231/*Δ^*^e-13&e-231^* embryos *Pdx1* expression was modestly decreased at E8.5 followed by a greater decrease at E9.5 and E10.5 (Fig. 4A). *Ptf1a* expression was similarly downregulated in all mutants from E9.5 to E11.5 (Fig. 4B). These findings corroborate the early role of *Sox17* in determining the size of the ventral pancreato-biliary progenitor cell pool prior to restriction to a biliary fate (Spence et al., 2009; Uemura et al., 2010), and indicate that ventral pancreatic organogenesis is greatly attenuated in the absence of either or both Sox17e-13 or Sox17e-231.

**Fig. 4.**
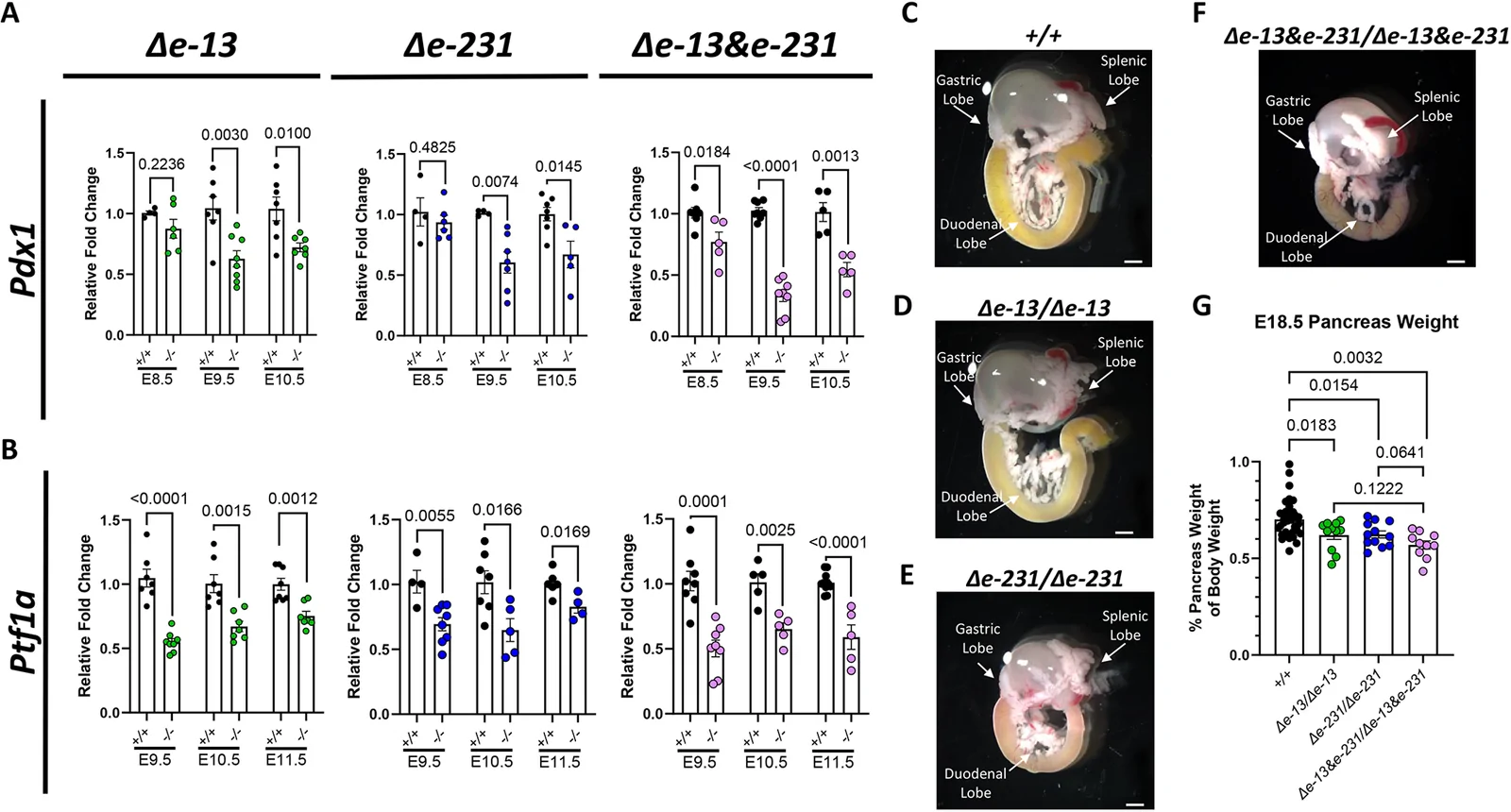
**Sox17e-13 and Sox17e-231 deletions diminish pancreas development.** (A) Whole-embryo RT-qPCR of *Pdx1* from E8.5 to E10.5. (B) Whole-embryo RT-qPCR of *Ptf1a* from E9.5 to E11.5. *Sox17^e-13^*: E8.5 +/+ *n*=4, −/− *n*=6; E9.5 +/+ *n*=7, −/− *n*=8; E10.5 +/+ *n*=7, −/− *n*=7; E11.5 +/+ *n*=8, −/− *n*=7; *Sox17^e-231^*: E8.5 +/+ *n*=4, −/− *n*=6; E9.5 +/+ *n*=4, −/− *n*=7; E10.5 +/+ *n*=7, −/− *n*=5; E11.5 +/+ *n*=6, −/− *n*=4; *Sox17^e-13&e-231^*: E8.5 +/+ *n*=7, −/− *n*=5; E9.5 +/+ *n*=8, −/− *n*=8; E10.5 +/+ *n*=5, −/− *n*=5; E11.5 +/+ *n*=10, −/− *n*=5. Each dot is one biological replicate. Each biological replicate is averaged from three technical replicates. Data presented as mean±s.e.m.; significance assessed by an unpaired *t-*test; all *P*-values displayed. (C-F) Representative images of E18.5 pancreata. Scale bars: 1 mm. (G) Percentage pancreas weight of body weight at E18.5. *Sox17^+/+^*, *n*=35; *Sox17*^Δ*e-13/*Δ*e-13*^, *n*=11; *Sox17*^Δ*e-231/*Δ*e-231*^, *n*=12; *Sox17*^Δ*e-13&e-231/*Δ*e-13&e-231*^, *n*=10. Each dot is one biological replicate. Data presented as mean±s.e.m.; significance assessed by one-way ANOVA; all *P*-values shown.

As final pancreas size is determined by the initial number of PDX1^+^ progenitors (Stanger et al., 2007), and our data showed decreased ventral pancreatic progenitors at E9.5 and E10.5 (Fig. 3), we measured pancreas mass at E18.5 to determine how the loss of either or both enhancers, and their effect on early fate segregation, led to later alterations in pancreas size. The size of E18.5 pancreata across mutant lines was smaller than that of wild-type animals (Fig. 4C-G), and images of mutant pancreata suggested a reduced size primarily of the partly ventral pancreatic-derived duodenal lobe, but not of the dorsal bud-derived gastric and splenic lobes (Dolenšek et al., 2015). Blood-glucose levels and glucose tolerance measured in adult *Sox17*^Δ*e-13/*Δ^*^e-13^* and *Sox17*^Δ*e-231/*Δ^*^e-231^* male animals were unchanged (Fig. S4), demonstrating that the modestly decreased pancreas size had no readily apparent effect on overall glucose homeostasis in metabolically unstressed adult animals. The lack of differences in overall pancreas size between the single and dual enhancer deletion animals (Fig. 4G) may reflect a lesser fractional contribution of the ventral bud to the overall organ size (Li et al., 2018).

### Sox17e-13 and Sox17e-231 differentially affect gallbladder development in single- and dual-deletion mutants

We next sought to determine whether the observed early alterations in numbers of biliary progenitor cells (Fig. 3C,D) affected final gallbladder size. Both gallbladder wet weight and 2D area were increased in 8- to 10-week-old *Sox17*^Δ*e-13/*Δ^*^e-13^* and *Sox17*^Δ*e-231/*Δ^*^e-231^* mice (Fig. 5A-C), but deletion of both enhancers in the *Sox17*^Δ*e-13&e-231/*Δ^*^e-13&e-231^* animals resulted in smaller or nearly absent gallbladders (Fig. 5A-C). These results are consistent with the earlier (E10.5) increase in number of biliary progenitors in single enhancer mutants and decrease in dual-deletion embryos (Fig. 3D). Histological analysis of *Sox17*^Δ*e-13/*Δ^*^e-13^*, *Sox17*^Δ*e-231/*Δ^*^e-231^* and *Sox17*^Δ*e-13&e-231/*Δ^*^e-13&e-231^* animals revealed no overt cellular morphological changes in hepatic or pancreatic architectures, or the gallbladder epithelium of *Sox17*^Δ*e-13/*Δ^*^e-13^* and *Sox17*^Δ*e-231/*Δ^*^e-231^* animals (Fig. S5). Notably, the gallbladder epithelium of dual-deletion *Sox17*^Δ*e-13&e-231/*Δ^*^e-13&e-231^* animals exhibited marked thickening and epithelial invaginations (Fig. S5D), consistent with decreases in *Sox17* expression altering the gallbladder epithelium (Uemura et al., 2020; Trinh et al., 2025; Zeng et al., 2025).

**Fig. 5.**
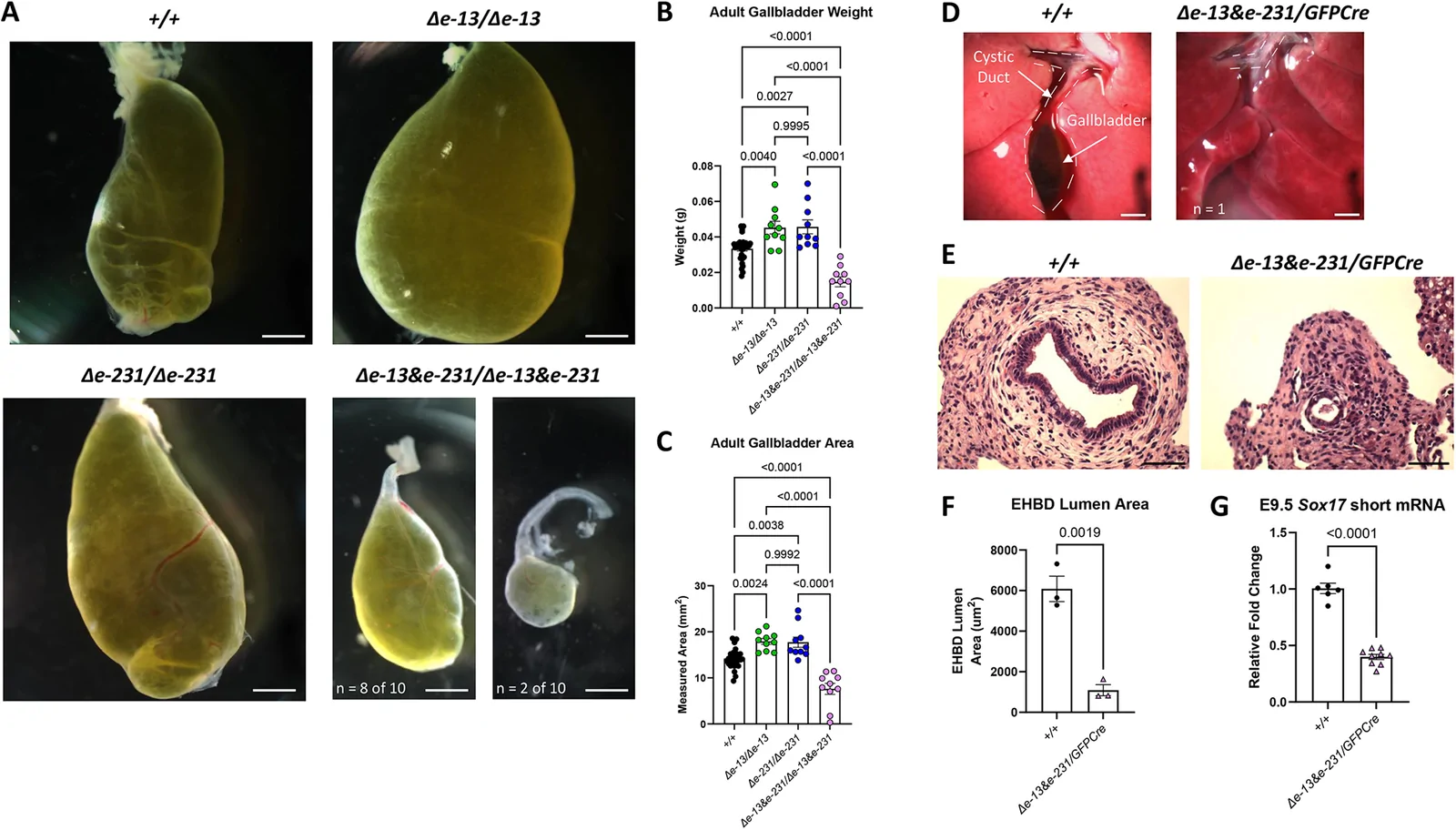
**The biliary system is differentially sensitive to changes in *Sox17* expression.** (A) Adult gallbladder images across mutant lines. *Sox17*^Δ*e-13&e-231/*Δ*e-13&e-231*^ has two phenotypes as shown, with numbers in the bottom left corner indicating the number of occurrences. Scale bars: 1 mm. (B) Quantitation of wet gallbladder weight (B) and 2D organ area (C). For both B and C: *Sox17^+/+^*, *n*=30; *Sox17*^Δ*e-13/*Δ*e-13*^, *n*=10; *Sox17*^Δ*e-231/*Δ*e-231*^, *n*=10; *Sox17*^Δ*e-13&e-231/*Δ*e-13&e-231*^, *n*=10. Each dot is one biological replicate. Data presented as mean±s.e.m.; significance assessed by one-way ANOVA; all *P*-values displayed. (D) Gallbladder and cystic duct agenesis in the only *Sox17*^Δ*e-13&e-231/GFPCre*^ animal that survived to adulthood. Scale bars: 1 mm. (E) Image of P2 extrahepatic bile ducts in *Sox17^+/+^* and *Sox17*^Δ*e-13&e-231/GFPCre*^. Scale bars: 50 μm. (F) Quantified lumen area of an extrahepatic bile duct, as shown in E. *n*=3 biological replicates per genotype; each biological replicate is the average of three technical replicates. Data presented as mean±s.e.m; significance assessed by unpaired *t*-test; *P*-value shown. (G) E9.5 whole-embryo RT-qPCR *Sox17* short mRNA measurements. *Sox17^+/+^*, *n*=6; *Sox17*^Δ*e-13&e-231/GFPCre*^ *n*=9. Each dot is one biological replicate. Data presented as mean±s.e.m., significance assessed by unpaired *t*-test; *P*-value shown.

### Sox17e-13 and Sox17e-231 promote EHBD formation

Biliary atresia is a disease in which EHBDs are blocked, constricted or absent, and is often lethal if left untreated (Bezerra et al., 2018; Tam et al., 2024). Decreased *Sox17* dosage is associated with biliary atresia in both humans and mice (Fried et al., 2020; Uemura et al., 2020; Glessner et al., 2023), and causes perinatal lethality in mice by postnatal day (P) 3 due to bile-induced hepatic necrosis (Uemura et al., 2013; Pattarapanawan et al., 2020; Trinh et al., 2025). Since impaired *Sox17* expression adversely affects EHBD formation, we utilized the null *Sox17^GFPCre^* allele to create a *Sox17* dosage-sensitized background. To test whether both enhancers contribute to EHBD formation, we crossed the *Sox17*^Δ^*^e-13&e-231^* allele with *Sox17^GFPCre/+^* mice and assessed survival of the *Sox17*^Δ*e-13&e-231*/GFPCre^ progeny at weaning (Table S2). While most *Sox17*^Δ^*^e-13&e-231/GFPCre^* compound heterozygotes died by P3, indicative of biliary atresia, a single *Sox17*^Δ^*^e-13&e-231/GFPCre^* mouse survived past weaning. At 8 weeks of age, this animal had gallbladder and cystic duct agenesis (Fig. 5D). To confirm the biliary atresia phenotype, we sacrificed *Sox17*^Δ^*^e-13&e-231/GFPCre^* animals at P2, and found reduced bile-duct luminal area, indicative of highly impaired EHBD development (Fig. 5E,F). Analysis of E9.5 *Sox17*^Δ^*^e-13&e-231/GFPCre^* embryos showed that *Sox17* short RNA expression was reduced by ∼60% (Fig. 5G), an amount previously shown to associate predictably with biliary atresia and its lethal impairments in EHBD development (Trinh et al., 2025). Together, these findings suggest lethal or near-lethal biliary atresia in *Sox17*^Δ^*^e-13&e-231/GFPCre^* compound heterozygotes, indicating that Sox17e-13 and Sox17e-231, in addition to being necessary for the formation of the ventral pancreas and gallbladder, contribute to the development of the EHBD system.

### *Sox17* dosage sensitization reveals similar potencies of Sox17e-13 and Sox17e-231

To identify any subtle differences in enhancer strength between Sox17e-13 and Sox17e-231, we explored the same gene dosage-sensitization strategy, crossing *Sox17^Δe-13/+^* and *Sox17^Δe-231/+^* mice with the *Sox17^GFPCre^* functional null allele. Measurement of *Sox17* short-form mRNA expression at E9.5 revealed a stepwise but non-significant decrease between *Sox17^GFPCre/+^*, *Sox17*^Δ^*^e-13/GFPCre^* and *Sox17*^Δ^*^e-231/GFPCre^* animals (Fig. 6A). In contrast to the lethality of the *Sox17^Δe-13&e-231/GFPCre^* genotype, both the *Sox17^Δe-13/GFPCre^* and *Sox17^Δe-231/GFPCre^* compound-heterozygous mice were viable (Table S2). Gross morphological analysis identified two similar but different phenotypes within these animals: either gallbladder hypoplasia or gallbladder agenesis with a normal cystic duct. All compound heterozygotes had smaller gallbladders compared with *Sox17^GFPCre/+^* mice, but no differences were observed between the *Sox17^Δe-13/GFPCre^* and *Sox17^Δe-231/GFPCre^* animals (Fig. 6B,C). The compound heterozygous phenotypes extended beyond those in *Sox17^GFPCre/+^* animals as they sometimes exhibited gallbladder agenesis with a normal cystic duct, suggesting differential sensitivity of the cystic duct and gallbladder to reduced *Sox17* expression. These findings again indicate similar functional potency for the Sox17e-13 and Sox17e-231 enhancers in driving *Sox17* expression to a level sufficient for proper gallbladder and EHBD formation.

**Fig. 6.**
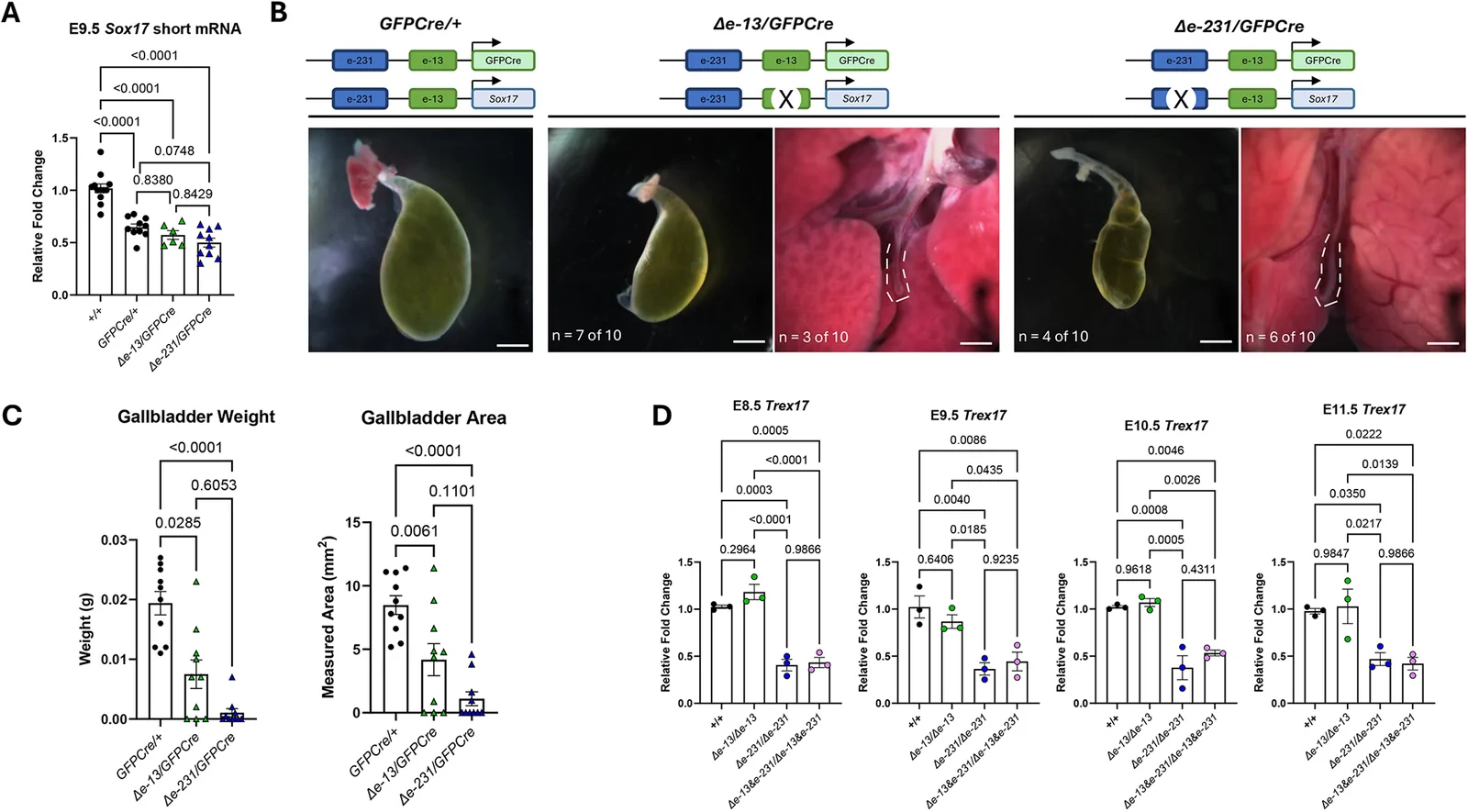
**Sox17e-13 and Sox17e-231 are functionally similar except for the differential regulation of *Trex17.*** (A) E9.5 whole-embryo RT-qPCR *Sox17* short mRNA measurements. *Sox17*^+/+^, *n*=12; *Sox17^GFPCre/+^*, *n*=10; *Sox17*^Δ*e-13/GFPCre*^, *n*=6; *Sox17*^Δ*e-231/GFPCre*^, *n*=10. Each dot is one biological replicate. (B) Images of adult gallbladders from *Sox17* heterozygous (*Sox17^GFPCre/+^*) and compound heterozygous animals. Schematics of compound heterozygous mutations are shown above the image. Both compound heterozygotes have two phenotypes, the numbers in the corner of each image represent the proportion of animals displaying each phenotype: Left shows gallbladder hypoplasia, right shows gallbladder agenesis with the presence of a cystic duct. Dashed lines highlight the presence of a cystic duct and lack of gallbladder outgrowth. Scale bars: 1 mm. (C) Quantification of wet gallbladder weight and 2D organ area. *n*=10 per genotype. Each dot is one biological replicate. (D) Whole-embryo RT-qPCR of *Trex17* from E8.5 to E11.5 across mutant lines. *n*=3 per genotype and timepoint. All data presented as mean±s.e.m.; significance assessed by one-way ANOVA; all *P*-values shown.

### Differential regulation of the lncRNA *Trex17* by Sox17e-13 and Sox17e-231

As dosage sensitization yielded no differences in enhancer potency, we next compared the effect of single enhancer deletions on expression of the mouse ortholog of human *TREX17* (Transcript Regulating Endoderm and activated by soX17), a functional lncRNA within the human *SOX17* TAD that is dispensable for *SOX17* activation. dCas9-mediated knockdown of *TREX17* in human ESCs has previously demonstrated reduced differentiation towards pancreatic progenitor cells (Landshammer et al., 2023). An enhancer orthologous to mouse Sox17e-231 lies within the human *TREX17* transcription unit (Fig. S6A). Hence, we sought to determine whether *Trex17* is expressed in mice and if it is regulated by Sox17e-13 and/or Sox17e-231. Performing a lift-over of human *TREX17* onto the mouse genome placed the mouse *Trex17* promoter region overlapping the conserved region at −229 kb (Fig. S6B, Fig. 1B) and identified mouse coordinates for all three exons (Fig. S6A,B). We next used a previously described human RT-qPCR assay (Landshammer et al., 2023) to design a comparable mouse *Trex17* assay and determined that *Trex17* is expressed during pancreato-biliary development (Fig. S6C,D). To determine whether *Trex17* is regulated by either Sox17e-231 or Sox17e-13, we performed RT-qPCR from E8.5 to E11.5 and found no change in *Sox17*^Δ*e-13/*Δ^*^e-13^* embryos but a persistent yet similar downregulation in *Sox17*^Δ*e-231/*Δ^*^e-231^* and *Sox17*^Δ*e-13&e-231/*Δ^*^e-13&e-231^* embryos (Fig. 6D). Downregulation of *Trex17* in *Sox17*^Δ*e-231/*Δ^*^e-231^* mutants at E8.5-E11.5 does not differentially influence the expression of pancreatic markers in comparison to *Sox17*^Δ*e-13/*Δ^*^e-13^* mutants (Fig. 4). The reduction in *Trex17* expression caused by deleting Sox17e-231 suggests either that *Trex17* regulates *Sox17* expression, or that *Trex17* regulatory elements are shared with *Sox17.* If so, any perturbation of shared *Sox17/Trex17* regulatory elements would also affect *Sox17* expression and downstream development. In either case, our findings indicate that Sox17e-231 is an enhancer for both *Sox17* and *Trex17*, whereas Sox17e-13 serves only as an enhancer for *Sox17*.

### Sox17e-13 and Sox17e-231 are functionally similar but have different enhancer grammar

Enhancer activity has been suggested to be determined by the affinity, order, orientation, spacing and number of CREs they contain, sometimes referred to as ‘enhancer grammar’ (Jindal and Farley, 2021). Given that both Sox17e-13 and Sox17e-231 are similar with respect to their length, epigenetic profiles, and TF binding, we compared the FOXA2 and GATA4 binding motifs within each enhancer, but found no consistency in their order, orientation, spacing or number (Fig. S7A,B). While the predicted affinities of the FOXA2 motifs were similar for both enhancers, Sox17e-13 had four GATA4 motifs of moderate affinity whereas Sox17e-231 contained only a single GATA4 motif of higher affinity (Fig. S7A,B). Comparison of the human orthologs of Sox17e-13 and Sox17e-231, both of which are functionally validated enhancers (Fig. S7C) (Luo et al., 2023; Kaplan et al., 2024), similarly revealed no consistent pattern other than the presence of several FOXA2 and GATA4 motifs (Fig. S7A,B). Moreover, the orientation and spacing of FOXA2 and GATA4 motifs within each enhancer are markedly dissimilar between mice and humans.

Finally, as the gallbladder is absent in many species (Mann et al., 1920; Higgins, 1926; Cebra et al., 2003; Higashiyama et al., 2018), we compared the Sox17e-13 and Sox17e-231 orthologs between species containing or lacking a gallbladder (Fig. S8A). Orthologous enhancer sequences were similar between species (Fig. S8B,C) with no overt differences in the predicted FOXA2 and GATA4 motif affinities (Fig. S8D,E).

## DISCUSSION

### Evolutionary conservation of the *SOX17/Sox17* locus

In humans, the *SOX17*-containing TAD is ∼336 kb in length and bounded by two tandem-oriented and highly conserved CTCF motifs (Rhodes et al., 2019; Wu et al., 2021; Landshammer et al., 2023). While mice contain two conserved CTCF motifs and a predicted chromatin loop in mouse ESCs of a similar length to that in humans (Fig. S9A) (Dixon et al., 2012), the CTCF motif locations and called loop boundaries do not perfectly align. Thus, our studies do not definitively define the murine *Sox17* TAD. However, using the two CTCF motifs as likely boundaries for the mouse TAD, we studied the evolutionary conservation of sequences within the demarcated ∼352 kb region and identified 38 conserved sequence stretches of at least 100 bp, two of which were studied here.

Sox17e-13 and Sox17e-231 were found to additively regulate expression of *Sox17* during a 2-day timeframe during which crucial events in HPB development occur (Fig. 7A). Whether these enhancers act solely to determine *Sox17* expression within biliary progenitors or also interact with other regulatory regions remains unknown. The pronounced effects of these two enhancers suggest that many, if not all, of the other 36 identified regions, and possibly additional shorter regions below our arbitrary length cutoff, interact in a yet-to-be defined manner to provide a robust modular regulatory architecture that drives the lineage-specific, temporal and quantitative expression of *Sox17* within different lineages (Niakan et al., 2010; Clarke et al., 2013; Corada et al., 2013; Irie et al., 2015; Chew et al., 2019).

**Fig. 7.**
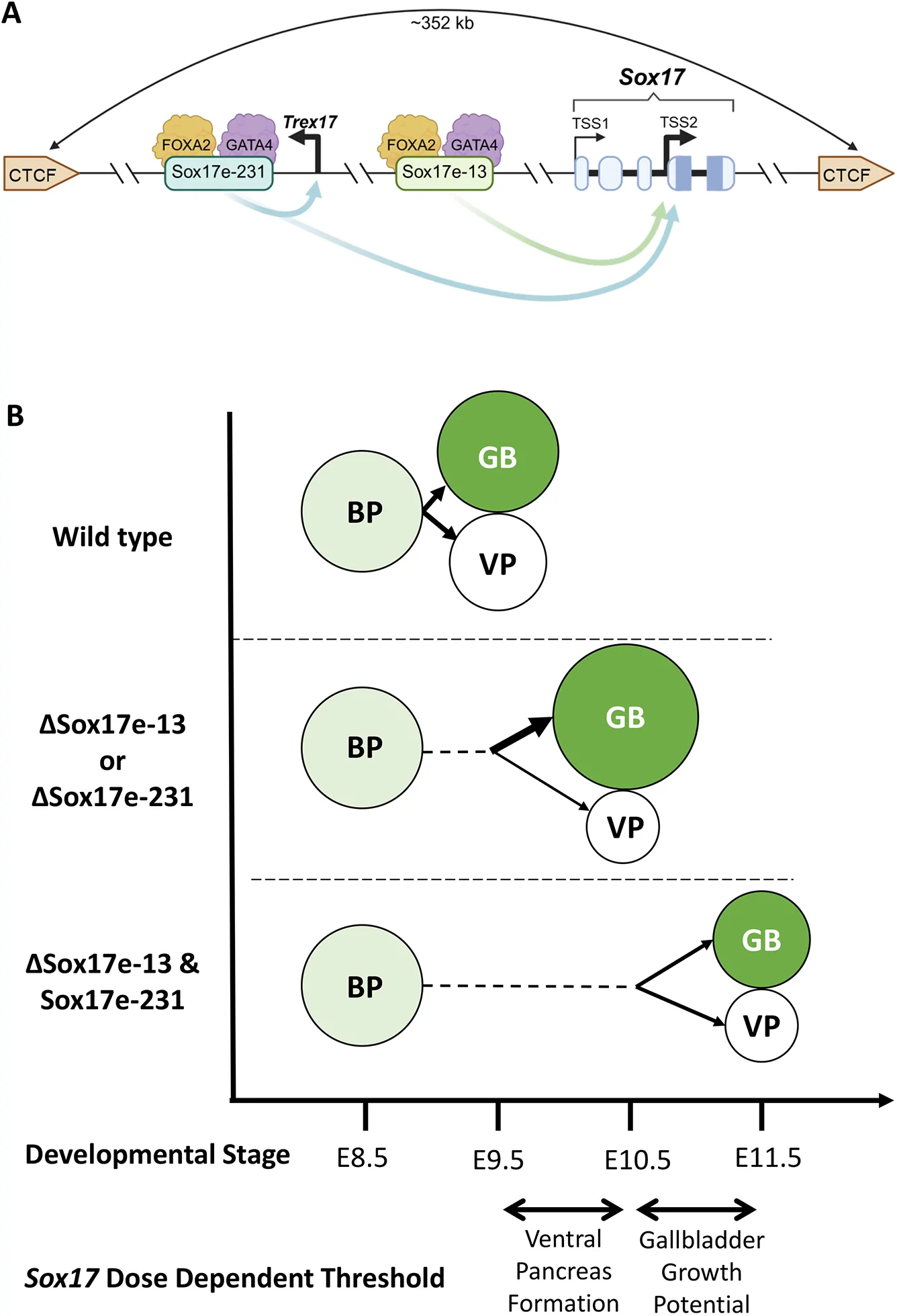
**Models of *Sox17* enhancer action and outcomes of enhancer deletions.** (A) Model of the likely mouse *Sox17* TAD, with boundaries denoted by CTCF binding sites and with FOXA2 and GATA4 bound to each enhancer. Sox17e-13 stimulates *Sox17* TSS2-driven expression, whereas Sox17e-231 stimulates both *Trex17* and *Sox17* TSS2-driven expression. Not to scale. Created in BioRender by Finnel, R., 2026. https://BioRender.com/mx790tx. This figure was sublicensed under CC-BY 4.0 terms. (B) Schematic of the delays in the ventral pancreato-biliary state transition and their phenotypic consequences across the various mutants. BP, bipotential progenitors; GB, gallbladder; VP, ventral pancreas. In the *Sox17^e-13^* or *Sox17^e-231^* null condition, the cell-state transition leads to an increase in biliary and a decrease in ventral pancreatic progenitors at E10.5. *Sox17^e-13&e-231^* null animals have a decrease in both biliary and ventral pancreatic progenitors at E10.5 with the persistence of a higher percentage of bipotential progenitors. Sox17e-13 and Sox17e-231 promote ventral pancreas formation between E9.5 and E10.5, as supported by decreased ventral pancreatic progenitors, decreased marker gene expression at E9.5 and E10.5, as well as a smaller pancreas at E18.5. Between E10.5 and E11.5 in the *Sox17^e-13&e-231^* null condition, a gallbladder growth potential may be conferred as embryos have decreased biliary progenitors and a sustained decrease in *Sox17* short mRNA expression, which manifests as a smaller gallbladder in the adult animal.

Our findings provide additional support for the assertion that single gene-occupancy TADs containing key developmental TFs serve as organizational recruitment and scaffolding domains for TFs and co-activators to build lineage-specific, permissive chromatin environments (Abassah-Oppong et al., 2024). The topological isolation of *Sox17* may provide an insulated environment for the surrounding chromatin to serve as a nexus for the input of cell type-specific regulatory information to respond quickly to spatial-temporal cues to maintain developmental tempo and lineage allocation during transitional states, a principle that may be applied broadly to key developmental regulators.

### Sox17e-13 and Sox17e-231 temporally and additively regulate formation of the ventral pancreas and gallbladder

Distributed enhancers, sometimes called shadow enhancers, drive partial or completely overlapping temporal and spatial expression patterns of a target gene (Kvon et al., 2021), ensuring phenotypic robustness under genetic or physiological stress (Hong et al., 2008; Barolo, 2012). Although separated by 218 kb, Sox17e-13 and Sox17e-231 were found to function similarly. While loss of a single enhancer has a minimal effect, loss of both enhancers leads to gallbladder hypoplasia, suggesting that the functional duplication ensures biliary phenotypic robustness. When the single and dual deletion alleles were intercrossed with a *Sox17* null allele, biliary atresia only occurred when both enhancers were absent, further suggesting that the presence of two distributed enhancers protects against *Sox17* dosage effects. However, since deletion of either enhancer alone had measurable effects on both ventral-pancreatic and biliary development, they are not completely functionally redundant, as demanded by a strict definition of shadow enhancers.

Sox17e-13 and Sox17e-231 contribute additively to the proper proportional allocation and developmental tempo of a relatively rapid, binary cell-lineage choice made between E9.5 and E10.5 by lineage-bipotential ventral pancreato-biliary progenitor cells to pursue either a ventral-pancreatic or biliary fate (Fig. 7B). These observations add a crucial time component to our understanding of how *Sox17* dosage affects pancreato-biliary development. Altering the timing and level of *Sox17* expression during a short critical window affects the subsequent sizes of the gallbladder and pancreas and is consistent with a prior study showing that the final size of the pancreas mirrors the number of cells allocated to the initiating pancreatic progenitor pool (Stanger et al., 2007).

The increase in biliary progenitor cell allocation caused by deleting either Sox17e-13 or Sox17e-231 likely involves an interplay with *Hes1*, a Notch effector gene required for biliary formation (Sumazaki et al., 2004). SOX17 can positively regulate HES1 levels in SOX17^+^/PDX1^+^ bipotential ventral pancreato-biliary progenitors, which in turn activates a negative feedback loop whereby HES1 spatially restricts SOX17^+^ cells to the ventral foregut between E8.5 and E9.5 by an undefined mechanism (Spence et al., 2009). Increasing *Sox17* expression via Sox17e-13 and Sox17e-231 between E8.5 and E9.5 may enable *Sox17* to reach a dosage threshold necessary to activate the *Hes1* negative feedback loop within SOX17^+^/PDX1^+^ bipotential progenitors to ensure proper spatial and quantitative segregation of ventral-pancreatic and biliary progenitor cells. Delay of this negative feedback mechanism would prolong the temporal co-expression of PDX1 and SOX17 within a sustained bipotential progenitor pool. As forced SOX17 expression within a PDX1^+^ domain directs a biliary over pancreatic fate decision (Spence et al., 2009), the likely delay of the HES1 negative feedback loop may convert cells originally destined for a ventral pancreatic progenitor state toward specification as biliary progenitors.

### Developmental stage-dependent gene-dosage thresholds for the actions of *Sox17* during early ventral pancreato-biliary fate selection

Alterations in the number of ventral pancreatic and biliary progenitor cells and the corresponding changes in pancreatic and gallbladder masses in adult animals suggest that Sox17e-13 and Sox17e-231, acting individually or together, affect formation of both tissues through primary and secondary effects (Fig. 7B). The first proposed *Sox17* dosage effect may occur between E9.5 and E10.5 when *Sox17* expression needs to exceed a threshold necessary to ensure the correct proportional allocation between the ventral pancreatic versus biliary lineages. The evidence supporting this first threshold is that both single-enhancer deletions, which reduce *Sox17* expression at E9.5-E10.5 but not at E11.5, are associated with larger gallbladders and smaller pancreata. Since our finding that reduced *Sox17* gene dosage impairs pancreas development is in alignment with previous reports (Spence et al., 2009) but not with our own prior study (Trinh et al., 2025), we repeated several of our previously reported measurements and obtained different results, resolving this discrepancy (Fig. S9B,C).

The second proposed *Sox17* dosage threshold may occur between E10.5 and E11.5 to regulate biliary outgrowth. The dual-deletion *Sox17*^Δ*e-13&e-231/*Δ^*^e-13&e-231^* embryos exhibit reduced *Sox17* expression at E9.5-E10.5 with continued reduction at E11.5, which by this time has become restricted to the developing gallbladder and extrahepatic biliary tract, leading to a smaller pancreas and smaller gallbladder. The E10.5-E11.5 time window for biliary bud outgrowth is supported by the failure of biliary bud elongation in *Lgr4^Gt/Gt^* mice at E10.5, which is due to loss of *Lgr4* expression within the biliary bud and surrounding mesenchyme (Yamashita et al., 2009). Our previous studies of *Sox17*^Δ*50/*Δ^*^50^* mice, which have gallbladder and cystic duct agenesis, further supports this time window. We demonstrated that SOX17 positive autoregulation is required for biliary bud elongation by E11.5 but is not necessary for initial segregation of the ventral pancreas and gallbladder at E9.5 (Trinh et al., 2025), findings that suggest a *Sox17*-mediated biliary outgrowth threshold between E10.5 and E11.5 is crucial for gallbladder and cystic duct organogenesis.

Differential necessity of the SOX17 positive autoregulatory loop at E9.5 and E11.5 may explain why the single enhancer-deletion animals recover to wild-type *Sox17* expression levels and have biliary outgrowth, whereas dual enhancer-deletion animals have continued downregulation of *Sox17* correlating with diminished biliary outgrowth (Figs 2 and 5). In single enhancer deletion animals, *Sox17* expression levels may exceed the critical threshold necessary for activation of the SOX17 positive autoregulatory loop, ensuring robust biliary outgrowth. In the dual enhancer deletion animals, *Sox17* levels may remain below the threshold necessary to activate the SOX17 positive autoregulatory loop, causing diminished gallbladder outgrowth.

The subtle differences in *Sox17* expression timing and progenitor allocation may also indicate a community effect-like phenomenon in which neighboring cells reinforce a shared differentiation state (Gurdon, 1988; Gurdon et al., 1993). In this context, *Sox17* levels, driven in large part by Sox17e-13 and Sox17e-231, may crucially promote expansion of the biliary progenitor pool to a size necessary for it to dynamically respond to extrinsic signals, such as Notch, Wnt and mesenchymal-to-epithelial signaling, ensuring robust extrahepatic biliary outgrowth (Kalinichenko et al., 2002; Sumazaki et al., 2004; Yamashita et al., 2009).

### Exquisite regional sensitivity and threshold-dependent collapse of gallbladder and EHBD development with reduced *Sox17* dosage

Introduction of the *Sox17^GFPCre^* null allele to each of our enhancer mutants produces a multitude of biliary phenotypes, ranging from gallbladder hypoplasia, gallbladder agenesis with a cystic duct, and biliary atresia, revealing the exquisite sensitivity of the developing EHBD system to *Sox17* dosage and a corresponding hierarchy of regional dosage susceptibility (gallbladder>cystic duct>common bile duct). Progressive reductions in *Sox17* levels drive increasingly severe outcomes, ranging from variable gallbladder defects in *Sox17*^Δ^*^e-13/GFPCre^* and *Sox17*^Δ^*^e-231/GFPCre^* to a nearly persistent loss of organismal viability in *Sox17*^Δ^*^e-13&e-231/GFPCre^*. Together, our findings define a spectrum of dosage-dependent failures within biliary development.

In our model, Sox17e-13 and Sox17e-231 function as protective buffers when the organism is faced with a *Sox17*-related genetic stress. At relatively normal or slightly reduced *Sox17* dosage, the Sox17e-13 and Sox17e-231 enhancers protect full-scale biliary development from *Sox17* dosage effects and may protect against extreme drops in *Sox17* expression that affect organismal viability. The co-existence of various phenotypes that teeter on the brink of developmental collapse in each compound heterozygous condition further supports a threshold model in which small perturbations in *Sox17* expression, which may arise from stochastic variation in gene expression or environmental modifiers, could be sufficient to significantly alter developmental outcomes. Our findings regarding the differential *Sox17* dosage sensitivity within gallbladder and EHBD development, paired with the recent finding that gallbladder hypoplasia with reduced *Sox17* expression causes mispatterning of the extrahepatic and intrahepatic ductal connectivity (Miyazaki et al., 2024), could provide a framework for better understanding how small variations in *Sox17* gene dosage contribute to the developmental etiology of biliary atresia, the leading basis for pediatric liver transplants (Bezerra et al., 2018).

### Functional equivalence despite divergent enhancer grammar

While Sox17e-13 and Sox17e-231 are functionally indistinguishable, they differ in their number and predicted affinities of FOXA2 and GATA4 motifs. These structural differences suggest that various combinations of FOXA2 and GATA4 sites may yield similar functional output. Comparing the mouse enhancers to their human counterparts, or the human Sox17e-13 and Sox17e-231 with each other, revealed no consistent FOXA2/GATA4 enhancer grammar. Given the large evolutionary time between mouse and human, the persistence of Sox17e-13 and Sox17e-231 in both species despite their divergent FOXA2/GATA4 enhancer grammar may reflect an evolutionary pressure to balance mutational tolerance while preserving regulatory function. This balance of mutational tolerance and functional preservation may arise from compensatory interactions between motif affinity and positioning to stabilize enhancer activity within the evolutionary defined window for proper ventral-pancreas and biliary development. Finally, the similarity of Sox17e-13 and Sox17e-231 among species that either contain or lack a gallbladder suggests that evolutionary loss of the organ is not due to divergent *Sox17 cis*-regulation and instead reflects a yet-to-be-defined developmental mechanism, as suggested by studies of mouse–rat interspecies chimeras, which revealed rat cells substantially contributed to gallbladder formation in a mouse milieu (Kobayashi et al., 2010; Wu et al., 2017).

### Concluding comments

Sox17e-13 and Sox17e-231 were identified from 38 conserved regions in the likely *Sox17* TAD and found to additively regulate a crucial developmental period in formation of the HPB system, namely the conversion of bipotential pancreato-biliary cells to unipotent biliary or ventral pancreatic fates. Our derivation of precise single and dual deletions in mice has revealed these enhancers function additively during a narrow time window to ensure that *Sox17* expression meets critical developmental thresholds involved in determining the tempo of developmental decision-making, the numbers of cells allocated to lineage-specific progenitor pools, and the final sizes of the gallbladder and ventral pancreas, according to the summary table in Fig. S9D. Such knowledge would be difficult if not impossible to obtain using pluripotent stem cell-derived organoids in which the normal spatial dynamics of interactive tissue formation processes are likely very incompletely rendered and developmental timing is imprecise, and in which it remains problematic to distinguish dorsal versus ventral pancreatic developmental programs, and where it is not currently possible to measure the effects of subtle changes in gene dosage and timing on overall organ size or mass.

### Study limitations

We used multi-species conservation to identify putative enhancer elements, which may not identify species-specific regulatory elements. Our arbitrary threshold that conserved regions must be at least 100 bp in length excludes shorter regions that may contain functionally important CREs. Lastly, our conclusions are based on loss-of-function mutations without complementary gain-of-function experiments.

## MATERIALS AND METHODS

### Conservation of the *SOX17*/*Sox17* gene locus

Human *SOX17* TAD boundaries were obtained from previously published information (Wu et al., 2021) and compared with human CTCF ChIP-seq data (see Table S3). Mouse CTCF ChIP seq data (see Table S3) was then used to identify mouse CTCF motifs that were then aligned to the human TAD boundaries using Clustal Omega (https://www.ebi.ac.uk/jdispatcher/msa/clustalo). The likely mouse TAD boundary coordinates were lifted over from mm10 to the human genome hg19, which overlapped with the reported human *SOX17* TAD boundaries. The mouse CTCF binding sites were further characterized using the JASPAR 2020 database, PhyloP and PhastCons tracks within the UCSC genome browser.

### Identification of conserved regions within the putative *Sox17* TAD

Conserved regions within the putative mouse *Sox17* TAD were identified using the ‘PhyloP 60-Way-Placental-Mammal’ and ‘PhastCons 60-Way-Placental-Mammal’ tracks within the UCSC genome browser. A conserved region was defined as (1) exhibiting a PhastCons local maxima ≥0.5; (2) having a local PhyloP local maxima ≥1.648; (3) being at least 100 bp in length; and (4) endpoints were called when no stretch of DNA within 200 bp met the PhyloP and PhastCons criteria. Conservation of each region was then quantified using the ‘Table Browser’ tool within the UCSC genome browser.

### Quantification of ATAC-seq, epigenetic ChIP and TF ChIP data within the putative *Sox17* TAD

Epigenetic and TF binding data were obtained from Cernilogar et al. (2019) (see Table S3) and the signal strength for each custom track at each conserved region was quantified using the ‘Table Browser’ tool within the UCSC genome browser. Signals for Fig. 1D were called when the maximum signal score was ≥25% of the local maximum score within ±1 Mb of *Sox17*.

### CRISPR mutagenesis

The three *Sox17* enhancer deletion alleles, *Sox17*^Δ^*^e-13^* (*Rr446^em1Mgn^*; MGI:7522923; mm10 chr1:4506670-4507060), *Sox17*^Δ^*^e-231^* (*Rr460^em1Mgn^*; MGI:7530991; mm10 chr1:4725005-4725319) and *Sox17*^Δ^*^e-13&e-231^* (*Rr606^em1Mgn^*; MGI:7863343; mm39 chr1: 4576921-4577289) (*Rr616^em1Mgn^*; MGI:8167012; mm39 chr1: 4795219-4795547), were generated by Vanderbilt Genome Editing Resource using CRISPR/Cas9-mediated genome editing. Fertilized zygotes from CD-1 outbred mice (Charles River #022) were electroporated with CRISPR/Cas9 ribonucleoprotein complexes and single-stranded DNA donor oligonucleotides. The *Sox17*^Δ^*^e-13^* allele was generated using two CRISPRevolution EZ sgRNAs (Synthego) at 4.0 μM each, wild-type SpCas9 protein (MilliporeSigma) at 4.0 μM, and a 5.0 μM Alt-R™ HDR donor oligo (IDT), all diluted in TE buffer and microinjected into embryos. For the *Sox17*^Δ^*^e-231^* allele, two Alt-R™ CRISPR-Cas9 crRNAs (IDT) at 2.0 μM each were combined with HiFi Cas9 protein (IDT) at 4.0 μM and a 10.0 μM donor oligo (IDT), all diluted in Opti-MEM™. The *Sox17*^Δ^*^e-13&e-231^* dual mutant alleles were generated using four Alt-R™ CRISPR-Cas9 crRNAs (IDT) at 1.5 μM each and HiFi Cas9 protein at 6.0 μM, diluted in Opti-MEM. Electroporated or microinjected embryos were transferred into pseudopregnant CD-1 females. Offspring were weaned at P21, genotyped by PCR to identify founders carrying the desired deletions, and mutations were confirmed by Sanger sequencing. Confirmed founders were backcrossed to CD-1 mice for at least three generations prior to establishing homozygous mutant lines. All animal procedures were approved and monitored by the Vanderbilt Institutional Animal Care and Use Committee (IACUC). See Table S3 for guide RNA and donor oligo sequences.

### Routine husbandry and genotyping

Mice were socially housed with a 12-h light-dark cycle. Mouse lines were maintained in an outbred Crl:CD1 (ICR), or CD-1^®^ IGS background (Charles River, model #022). Genotypes for adult *Sox17*^Δ^*^e-13^*, *Sox17*^Δ^*^e-231^* and *Sox17^GFPCre^* mice were determined by automated qPCR by Transnetyx, whereas genotyping of embryos was done by PCR. All genotyping for *Sox17*^Δ^*^e-13&e-231^* was done by PCR. EconoTaq PLUS 2X Master Mix (Biosearch Technologies, 30035) was used for PCR reactions following standard protocol. PCR products were analyzed using 2% agarose gel electrophoresis. Genotyping primer sequences are shown in Table S3.

### Embryo collections

Timed matings were performed to obtain stage-specific embryos. The presence of a vaginal plug served to define E0.5. Embryonic tissues were micro-dissected at the desired developmental timepoints in cold PBS (Corning, 46-013-CM) and staged according to Theiler staging criteria.

### RT-qPCR

RNA was extracted from samples using Promega Maxwell 16 LEV simply RNA Purification Kit (Promega, AS1280) prior to conversion to cDNA via the High-Capacity cDNA Reverse Transcription Kit (Thermo Fisher Scientific, 4368814). cDNAs underwent qPCR using SYBR Green PCR Master Mix (Thermo Fisher Scientific, 4309155) on a Bio-Rad CFX real time PCR machine. The RT-qPCR program utilized 95°C for 10 min followed by 45 cycles of 95°C for 15 s and 60°C for 1 min (see Table S3 for primers). RT-qPCR amplicons spanned exon junctions when possible, and amplicons were validated by gel electrophoresis. The ΔΔCt method was used to calculate relative fold changes using *Hprt1* as a housekeeping gene. Wild-type littermate samples were used for normalization. Each biological replicate is the average of three technical replicates.

### Immunofluorescence staining

Immunofluorescence staining was performed as previously described (Trinh et al., 2025) using the antibodies and dilutions listed in Table S3. Progenitors were quantified using the ‘Cell Counter’ tool within Fiji. Progenitor cells were quantified first by marking single-positive SOX17^+^ or PDX1^+^ cells. Cells that were marked by both SOX17 and PDX1 were counted as double positive and classified as bipotential pancreato-biliary progenitors. The pancreato-biliary bud was defined as spanning from the first sectional observation of SOX17^+^ cells to the final sectional observation of SOX17^+^ cells. Each replicate consists of quantified progenitors across multiple sections. Cell proportions were calculated using the following denominators: E9.5 wild-type replicates: 961, 952, 1191 and 915 cells; E9.5 *Sox17*^Δ*e-13/*Δ^*^e-13^* replicates: 628, 734, 762 and 592 cells; E9.5 *Sox17*^Δ*e-231/*Δ^*^e-231^* replicates: 347, 1117 and 814 cells; E9.5 *Sox17*^Δ*e-13&e-231/*Δ^*^e-13&e-231^* replicates: 171, 125, 299 and 160 cells; E10.5 wild-type replicates: 1251, 1241 and 2092 cells; E10.5 *Sox17*^Δ*e-13/*Δ^*^e-13^* replicates: 2078, 1113 and 2130 cells; E10.5 *Sox17*^Δ*e-231/*Δ^*^e-231^* replicates: 896, 713 and 335 cells; E10.5 *Sox17*^Δ*e-13&e-231/*Δ^*^e-13&e-231^* replicates: 726, 586 and 457 cells.

### Pancreas characterization at E18.5

Timed matings were performed with the presence of a vaginal plug denoting E0.5. Embryos were harvested 18 days later and micro-dissected on cold PBS and staged according to Theiler criteria. Embryos were then placed in a dry 12-well plate on ice and weighed using a Sartorius Entris II essential precision balance. Embryos were further micro-dissected in cold PBS to obtain the stomach, pancreas and duodenum, which were promptly imaged. Pancreata were then collected and placed back into the 12-well plate with cold PBS. Pancreata were weighed following a blot dry. The percentage of pancreas weight over body weight was calculated while being unaware of genotype. Each datapoint represents one individual embryo.

### Gallbladder and EHBD imaging, calculations and staining

Animals were fasted overnight prior to gallbladder dissection from 8- to 10-week-old mixed-sex littermates and placed into cold PBS. Tissues were imaged using a Leica MZ 16 FA microscope. Gallbladders were then blot dried and weighed using a Sartorius Entris II essential precision balance. Area measurements were quantified using the polygon tool within Fiji. P2 EHBDs were collected in cold PBS and fixed in 10% formalin overnight before being placed in 70% ethanol. Tissues were processed by Vanderbilt Tissue Pathology Shared Resource (TPSR) using established protocols. Hematoxylin & Eosin-stained slides were imaged on a Zeiss Axioplan 2 upright microscope with a QImaging Retiga Exi camera. Luminal area was quantified using the polygon tool within Fiji. Each biological replicate represents an average of three technical replicates.

### Identification and characterization of mouse *Trex17*

The human *TREX17* sequence (Landshammer et al., 2023) (GRCh37/hg19) was lifted over to the mouse genome (GRCm38/mm10) using the LiftOver tool within the UCSC genome browser, and orthologous exonic sequences were identified. Expression of human and mouse *Trex17/TREX17* expression was validated by PCR with 2% gel electrophoresis with species-specific primers targeting exon 1 (primers in Table S3), with human primers targeting *Trex17/TREX17* expression in definitive endoderm and mouse primers targeting E10.5 embryos. Expression of mouse *Trex17* was confirmed by RT-qPCR (primers in Table S3) between E8.5 and E11.5.

### Generation of human definitive endoderm

H9 human ESCs (WiCell, WA09) were differentiated to endoderm and were seeded on a 6-well plate precoated with Growth Factor Reduced Matrigel (Corning, 354230) at passage 30. Cultures were maintained for 4 days in mTESR1 medium (STEMCELL Technologies, 85850) prior to passaging. Cells were disassociated using Gentle Cell Dissociation Reagent (GCDR) (STEMCELL Technologies, 100-1077) and plated at 2.1×10^6^ cells per well to initiate definitive endoderm differentiation with the STEMdiff Definitive Endoderm Kit (STEMCELL Technologies, 05110). Cells were collected at day 0 and day 5 and processed for RNA isolation, cDNA synthesis and RT-qPCR as described above. The H9 human ESCs were not authenticated in-house but were routinely tested for contamination.

### Glucose measurements and glucose tolerance test

Fed blood glucose measurements were obtained from 8- to 10-week-old male mice using an AccuCheck Aviva glucometer and its associated test strips the night prior to dissection. Animals were fasted overnight. The next morning, mice were weighed using a Sartorius Entris II essential precision balance, and a fasted blood glucose measurement was obtained. Mice were then intraperitoneally injected with 1.0 mg/g body mass of 30% D-glucose solution, followed by blood-glucose measurements taken at 0, 15, 30, 60, 90 and 120 min.

### Histochemical staining of embryos, liver, pancreas and gallbladder

For liver, pancreas and gallbladder staining, organs were dissected from 8- to 10-week-old littermates and placed into cold PBS on ice. Organs were fixed in 10% formalin overnight before being placed in 70% ethanol. The Vanderbilt Translational Pathology Shared Resource embedded the samples in paraffin and performed Hematoxylin & Eosin staining. Slides were then imaged on a Zeiss Axioplan 2 upright microscope with a QImaging Retiga Exi camera and surveyed for obvious tissue morphological abnormalities.

### Enhancer grammar analysis

Relative binding site affinity was obtained from the UniProbe database (http://the_brain.bwh.harvard.edu/uniprobe/browse.php) for FOXA2 (UniPROBE accession #UP00073) and GATA4 (UniPROBE accession #UP01604). Relative affinity was calculated by taking the mean intensity score for our sequence of interest over the highest mean intensity score. Identification and characterization of FOXA2 and GATA4 motifs were performed using the JASPAR 2020 track within the UCSC genome browser.

### Identification of orthologous human SOX17 enhancers

Genomic coordinates were obtained for the two human endodermal enhancers SOX17e+2 and SOX17e+10 (Luo et al., 2023), the pancreas enhancer Sox17e-233 (Kaplan et al., 2024) and eSox17 from Landshammer et al. (2023). Coordinates of published human enhancers were lifted over to mouse genome mm10 and compared to mouse Sox17e-13 and Sox17e-231 to determine whether their sequences overlap. Conversely, mm10 coordinates of Sox17e-13 and Sox17e-231 were lifted over to hg19 to identify human orthologous sequences that overlapped the genomic coordinates of the above enhancers.

### Gallbladder evolution analysis

Genomic coordinates of mouse Sox17e-13 and Sox17e-231 (mm10) in the UCSC genome browser were lifted over to squirrel (*Spermophilus tridecemlineatus*, speTri2), rat (*Rattus norvegicus*, rn7) and alpaca (*Vicugna pacos*, vicPac2). Multispecies alignment was performed with Clustal Omega (https://www.ebi.ac.uk/jdispatcher/msa/clustalo). Mouse FOXA2 and GATA4 sites were identified using the JASPAR 2020 track. The relative affinities of FOXA2 and GATA4 binding sites were calculated as described above.

### Statistics, data visualization, and illustrations

The statistical test, exact *n*, and what the *n* represents are described in the figure legends. All statistics and visualization were performed in GraphPad Prism 10 software.

## Supplementary Material



10.1242/develop.205751_sup1 Supplementary information
