# Supplementary material for: Two distributed enhancers of Sox17 additively regulate the developmental fate of bipotential ventral pancreato-biliary progenitor cells
Source: Development. 2026 Sep 18;153(18):dev205751. doi: 10.1242/dev.205751 (PMC13680832; doi:10.1242/dev.205751)
Supplement: Supplementary file 1 [file develop-153-205751-s1.pdf]

A)

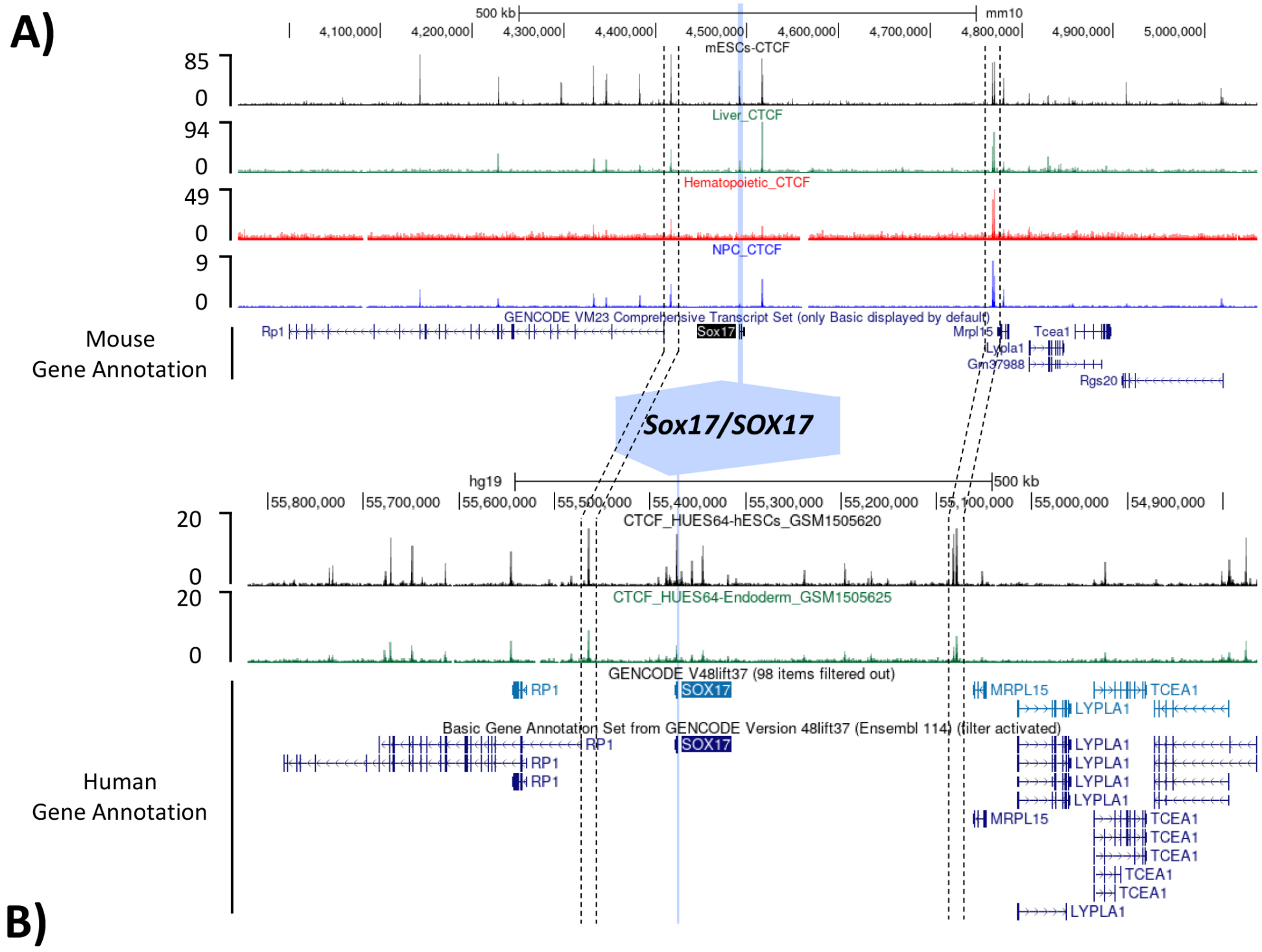

B)

Relative CTCF Site Location	Mouse Coordinates mm10	Mouse CTCF Sequence	Mouse CTCF JASPAR Score	LiftOver Human Coordinates hg19	Human CTCF Sequence	Human CTCF JASPAR Score
Telomeric TAD Boundary	chr1:4416902-4416934	GCGCCACCTTGTGGCCTGCACGCGTTGGTGCAC	825	chr8:55464387-55464419	CTGCACTGATGCTCATGCGCCACAAGATGGAGC	798
Within *Sox17* Gene Body	chr1:4492106-4492120	AGCGCTGGGGGGCGC	379	chr8:55372172-55372186	GCGCCACCCAGCGCC	381
Upstream of *Sox17*	chr1:4516735-4516749	GCCAATAGGTGGCGC	631	chr8:55344463-55344477	GCGCCACCTATTGGT	606
Centromeric TAD Boundary	chr1:4768546-4768576	TGTCACCTAGTGGTCACACACGGCACTGCAG	967	chr8:55082039-55082069	TTGCAGTACCACATCTTGAACAATAGGTGGAGC	767

C)

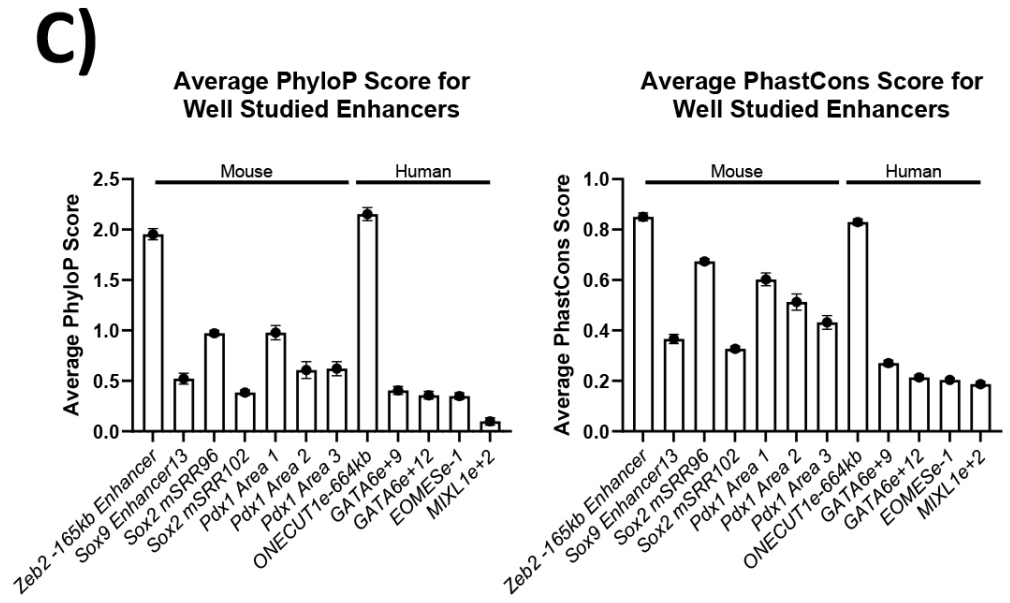

Mouse Enhancer	Target Gene	100bp minimum?	Met PhyloP Minimum?	Met PhastCons Min?
-165 Enhancer	*Zeb2*	Yes	Yes	Yes
Enhancer13	*Sox9*	Yes	Yes	Yes
mSRR96	*Sox2*	Yes	Yes	Yes
mSRR102		Yes	Yes	Yes
Area 1	*Pdx1*	Yes	Yes	Yes
Area 2		Yes	Yes	Yes
Area 3		Yes	Yes	Yes

Human Enhancers	Target Gene	100bp minimum?	Met PhyloP Minimum?	Met PhastCons Min?
ONECUT1e-664kb	*ONECUT1*	Yes	Yes	Yes
GATA6e+9	*GATA6*	Yes	Yes	Yes
GATA6e+12	*GATA6*	Yes	Yes	Yes
EOMESe-1	*EOMES*	Yes	Yes	Yes
MIXL1e+2	*MIXL1*	Yes	Yes	Yes

D)

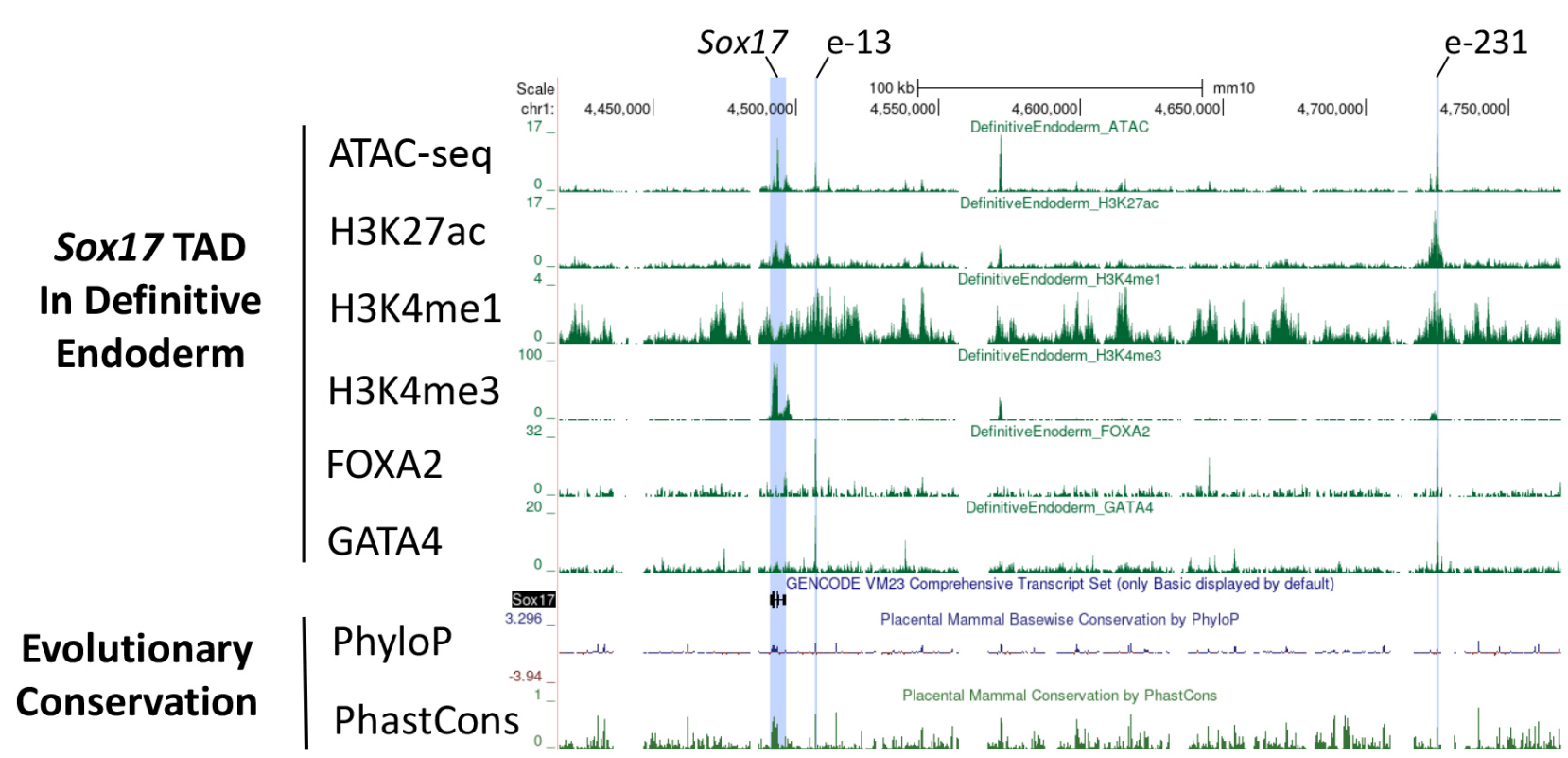

Development • Supplementary information

Fig. S1. Identification and characterization of the putative mouse *Sox17* TAD. A) Comparative mapping between the known human *SOX17* and likely mouse *Sox17* TADs. CTCF ChIP-seq data near mouse (top) and human (bottom) *Sox17*. Approximate viewing windows mm10 chr1:3,949,636-5,046,961 and hg19 chr8:54,764,156-55,821,372. Blue highlight over *Sox17/SOX17* gene body. Dashed lines indicate the corresponding putative TAD boundary CTCF binding locations. NPC, neural progenitor cells. B) Characterization of the conserved CTCF motifs shown in Fig 1A. C) PhyloP and PhastCons scores of select enhancers (left) and their ability to be identified by our conservation rules (right). D) Signal intensities used for quantification in Fig 1C. Blue highlights indicate *Sox17*, Sox17e-13, and Sox17e-231. Approximate viewing window mm10 chr1:4,416,900-4,768,576.3

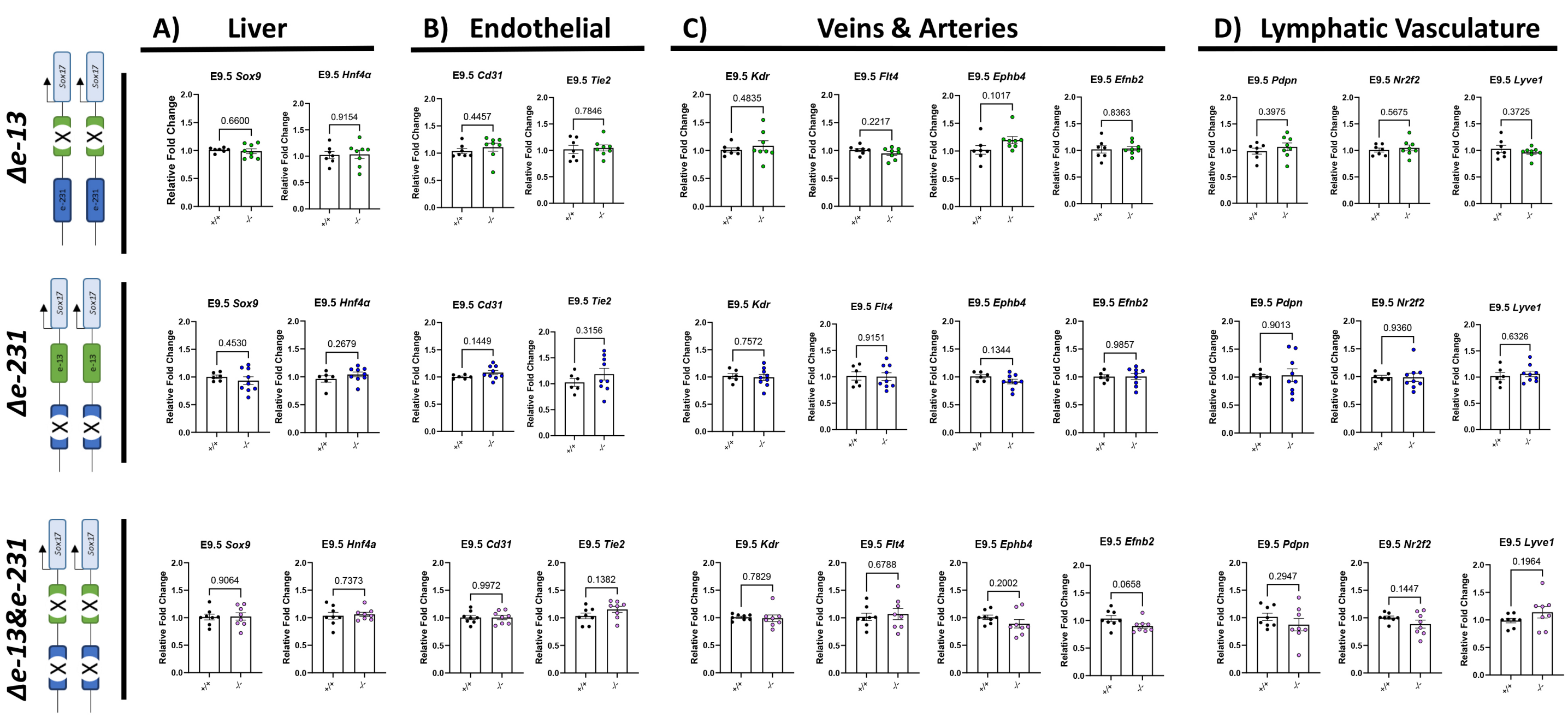

Fig. S2. Enhancer deletion does not affect liver or vasculature gene expression. Whole embryo RT-qPCR at E9.5 for A) liver markers, B) endothelial markers, C) venous & arterial markers, and D) lymphatic vasculature markers. *Sox17*$^{\Delta e\text{-}13}$: +/+ n=7, -/- n=8. *Sox17*$^{\Delta e\text{-}231}$: +/+ n=6, -/- n=9. *Sox17*$^{\Delta e\text{-}13\&e\text{-}231/\Delta e\text{-}13\&e\text{-}231}$: +/+ n=8, -/- n=8. Each dot is one biological replicate (one embryo). Each biological replicate is averaged from three technical replicates. Unpaired Student's *t*-test, all *p*-values shown, data is mean ± s.e.m.

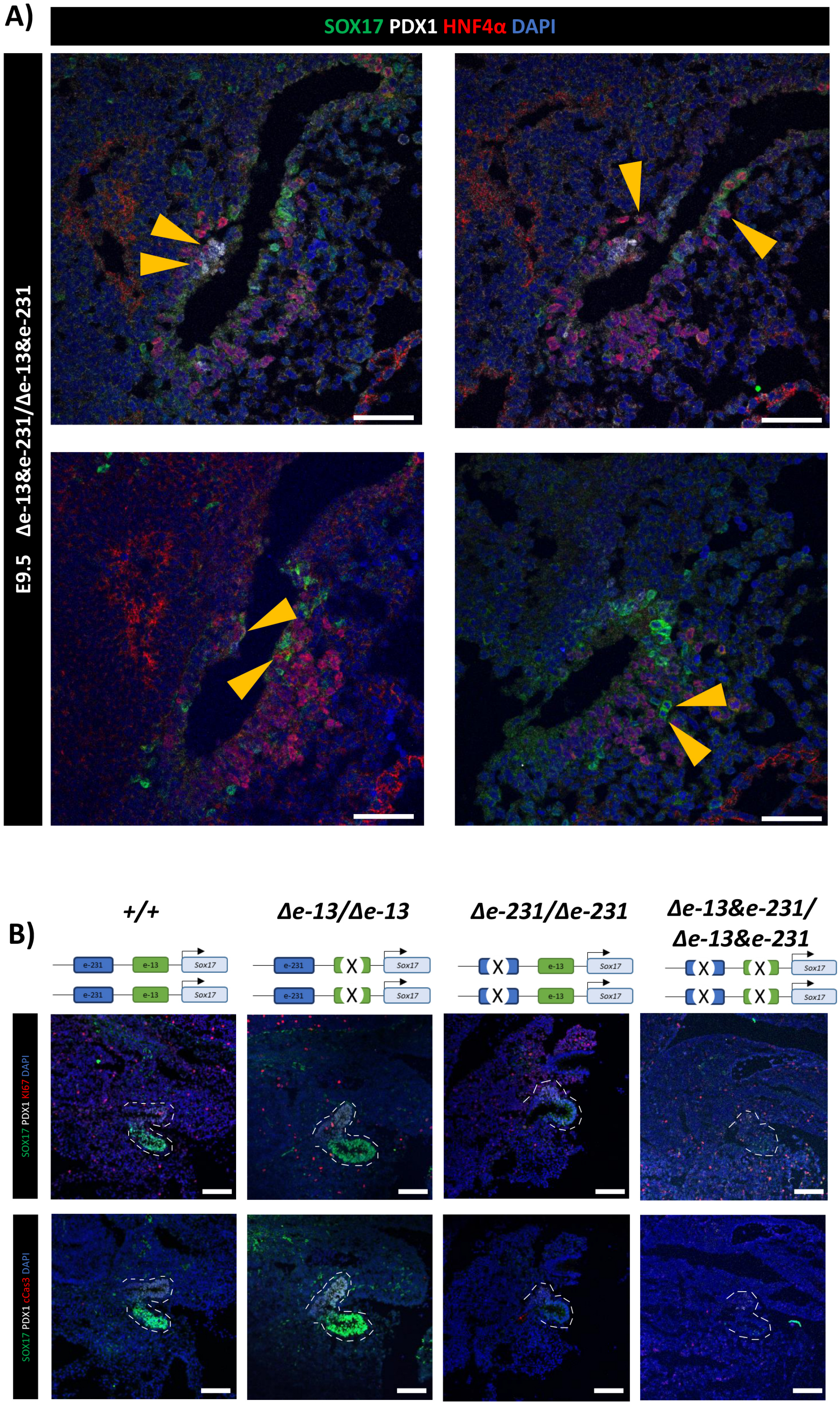
A)
SOX17 PDX1 HNF4α DAPI
E9.5 Δe-13&e-231/Δe-13&e-231
B)
+/+
Δe-13/Δe-13
Δe-231/Δe-231
Δe-13&e-231/
Δe-13&e-231
e-231
e-13
Sox17
SOX17 PDX1 Ki67 DAPI
SOX17 PDX1 cCas3 DAPI

Fig. S3. Organ boundary abnormality in the *Sox17*$^{\Delta e\text{-}13\&e\text{-}231/\Delta e\text{-}13\&e\text{-}231}$ condition and no overt changes in proliferation or apoptosis at E10.5. A) E9.5 immunofluorescent staining of one *Sox17*$^{\Delta e\text{-}13\&e\text{-}231/\Delta e\text{-}13\&e\text{-}231}$ embryo for SOX17 (green, biliary), PDX1 (white, pancreas), HNF4α (red, liver), and DAPI (blue, nucleus). Scale bar, 50 µm. Gold arrowheads indicate progenitors outside their anticipated normal organ domain. n=1 for this observation, images are adjacent serial sections from one embryo. Scale bar, 100 µm. B) E10.5 immunofluorescent staining of enhancer mutants for SOX17 (green, biliary), PDX1 (white, pancreas), Ki67 (red, top row, proliferation), cleaved-Caspase 3 (cCas3, red, bottom row, apoptosis) and DAPI (blue, nucleus). Scale bar, 100 µm.

Development • Supplementary information

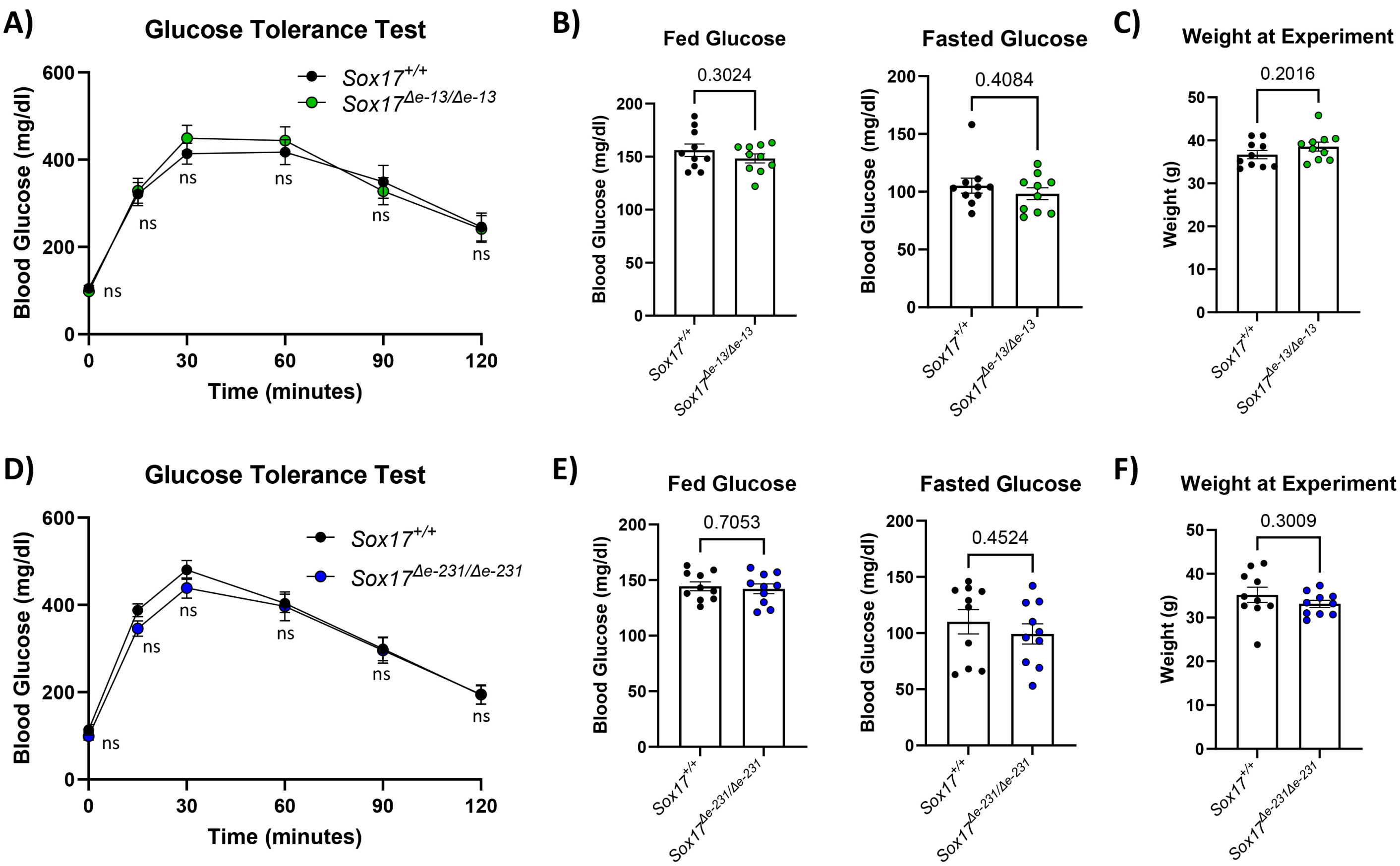

Fig. S4. Reduced pancreas size does not impair overall function in single enhancer-deletion mutants. A) Glucose tolerance test of 8–10-week-old male animals. n=10 per genotype and timepoint. Multiple unpaired *t*-tests. 0 min *p*=0.3827; 15 min *p*=0.8439; 30 min *p*=0.3661; 60 min *p*=0.5420; 90 min *p*=0.6629; 120 min *p*=0.9196. B) Fed and fasted glucose measurements. n=10, unpaired *t*-test. C) Weight of animals after overnight fast prior to fasted glucose measurement and glucose tolerance test. n=10, unpaired *t*-test. D) Glucose tolerance test of 8–10 week-old male animals. n=10 per genotype and timepoint. Multiple unpaired *t*-tests. 0 min *p*=0.3546; 15 min *p*=0.0844; 30 min *p*=0.2123; 60 min *p*=0.8649; 90 min *p*=0.9368; 120 min *p*=0.9820. E) Fed and overnight fasted glucose measurements. n=10, unpaired *t*-test. F) Weight of animals after overnight fast prior to fasted glucose measurement and glucose tolerance test. n=10, unpaired *t*-test. Each dot is one biological replicate. All data is mean ± s.e.m.

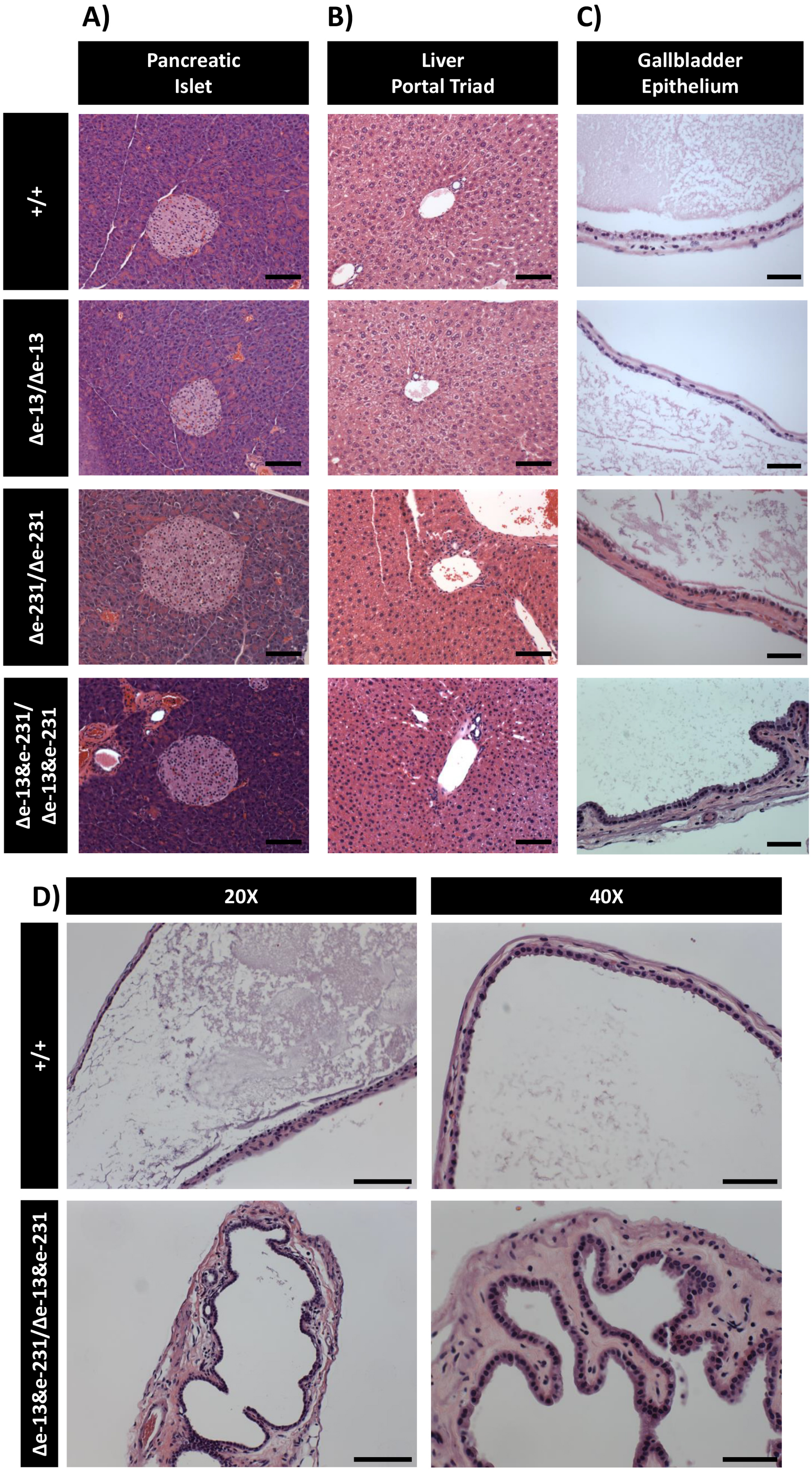
A)
B)
C)
Pancreatic Islet
Liver Portal Triad
Gallbladder Epithelium
+/+
Δe-13/Δe-13
Δe-231/Δe-231
Δe-13&e-231/ Δe-13&e-231
D)
20X
40X
+/+
Δe-13&e-231/Δe-13&e-231

Fig. S5. Enhancer deletion alters the *Sox17*$^{\Delta e\text{-}13\&e\text{-}231/\Delta e\text{-}13\&e\text{-}231}$ gallbladder epithelium. A) H&E staining of pancreatic islets, scale bar 100 µm. B) H&E staining of liver portal triads, scale bar 100 µm. C) H&E staining of gallbladder epithelium, scale bar 50 µm. All samples from 8–10 week-old animals. n=3 per genotype were surveyed. D) H&E staining of the gallbladder epithelium of 8-week–old wildtype and *Sox17*$^{\Delta e\text{-}13\&e\text{-}231/\Delta e\text{-}13\&e\text{-}231}$ littermates. Left images are taken to capture as much of the organ as possible, scale bar 100 µm. Right images focus on one subsection of the epithelium, scale bar 50 µm.

Development • Supplementary information

A)

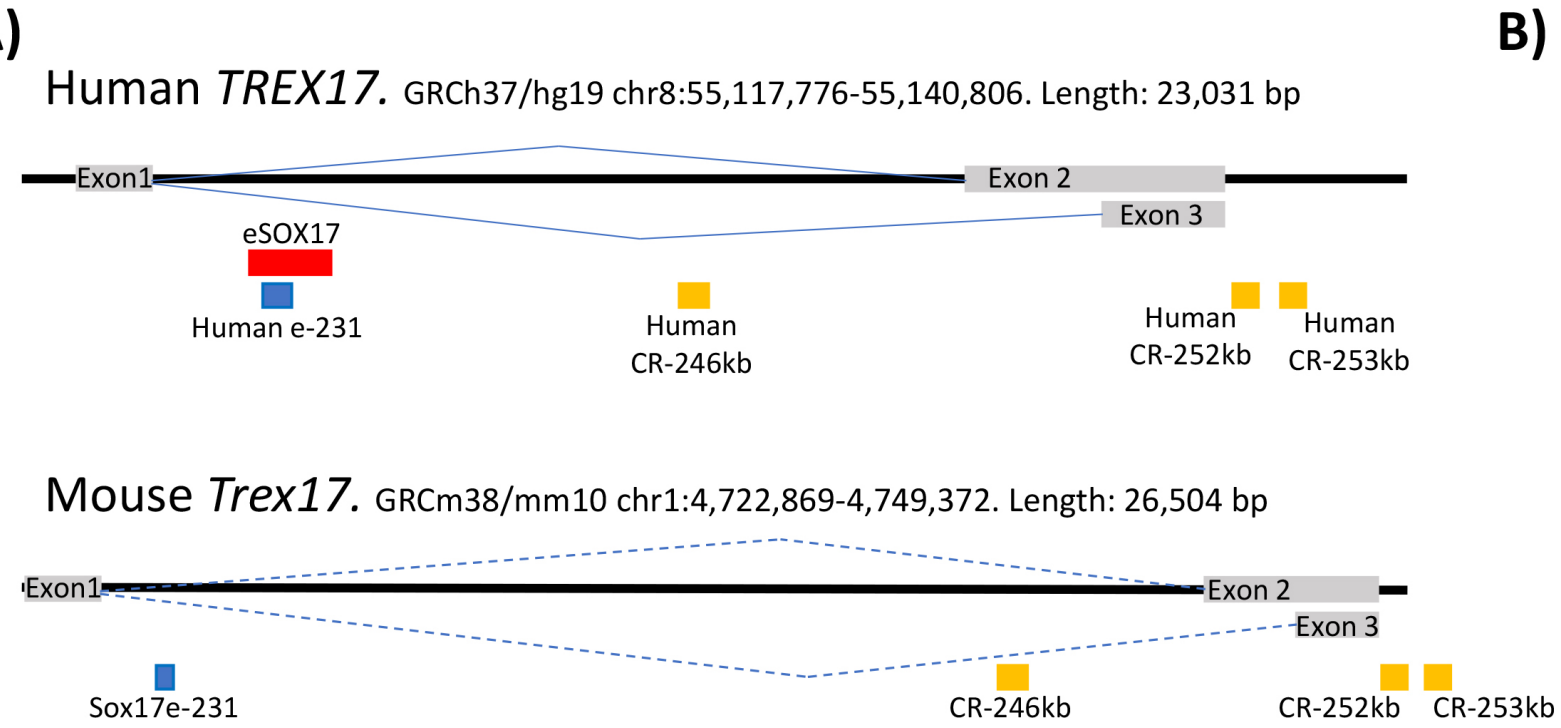

B)

TREX17 Feature	Human *TREX17* Coordinates GRCh37/hg19 Landshammer et al., 2023.	Proposed Mouse *Trex17* Coordinates GRCm38/mm10
Promoter	chr8:55,140,804-55,141,678	chr1:4,721,719-4,722,871
Exon 1	chr8:55,139,648-55,140,801	chr1:4,722,874-4,723,988
Exon 2	chr8:55,123,254-55,127,207	chr1:4,742,932-4,745,896
Exon 3	chr8:55,123,249-55,125,145	chr1:4,744,513-4,745,896
Sox17e-231	chr8:55,137,553-55,137,919	chr1:4,725,005-4,725,319

C) D)

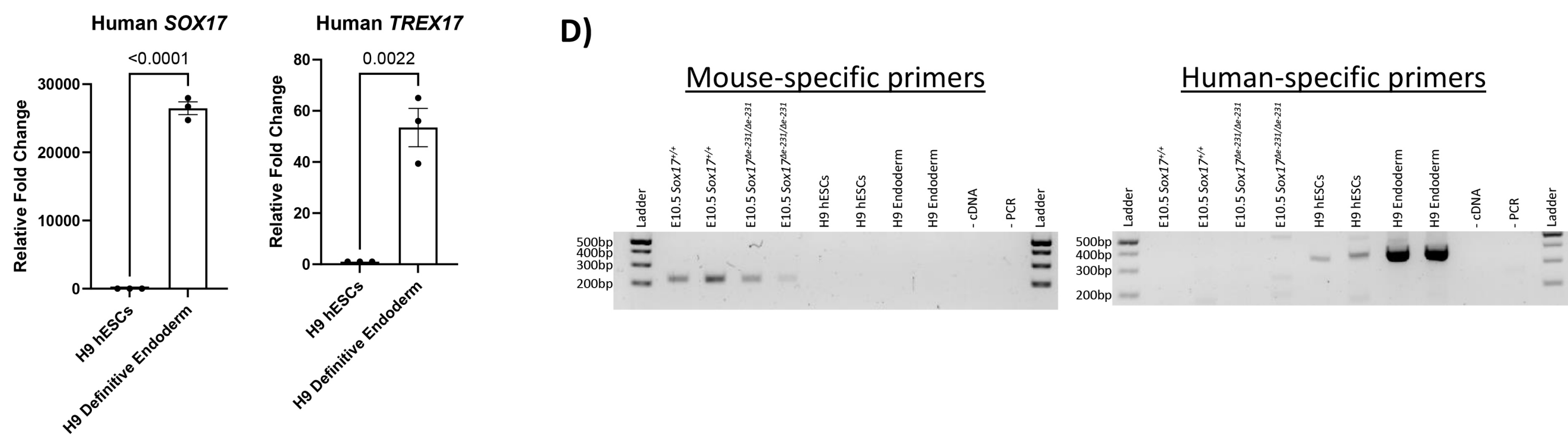

Fig. S6. Identification of murine *Trex17*. A) Schematic comparing human and mouse *TREX17/Trex17*. eSOX17, in red, is an enhancer identified by Landshammer et al. (2023), which overlaps with the human ortholog of Sox17e-231 shown in blue. Locations of human orthologues of conserved regions (CRs) identified in Fig 1, identified via the LiftOver tool within the UCSC genome browser, are shown in gold. B) Genomic locations of *TREX17* identified from Landshammer et al., 2023 and their LiftOver location in the mouse genome. The LiftOver location of the murine *Trex17* promoter partially overlaps with the human orthologue of CR-229kb. C) RT-qPCR from human H9 hESCs or H9 differentiated to definitive endoderm. n=3 per condition and transcript surveyed. Each biological replicate is the average of three technical replicates and normalized to *Hprt*. Unpaired *t*-test, data is mean ± s.e.m. D) PCR against human and mouse Exon 1 of *Trex17/TREX17* using species-specific primers. cDNA from whole-embryo cell lysate was utilized as template material to exclude genomic DNA and determine if the *Trex17/TREX17* is expressed. n=2 per condition as shown.

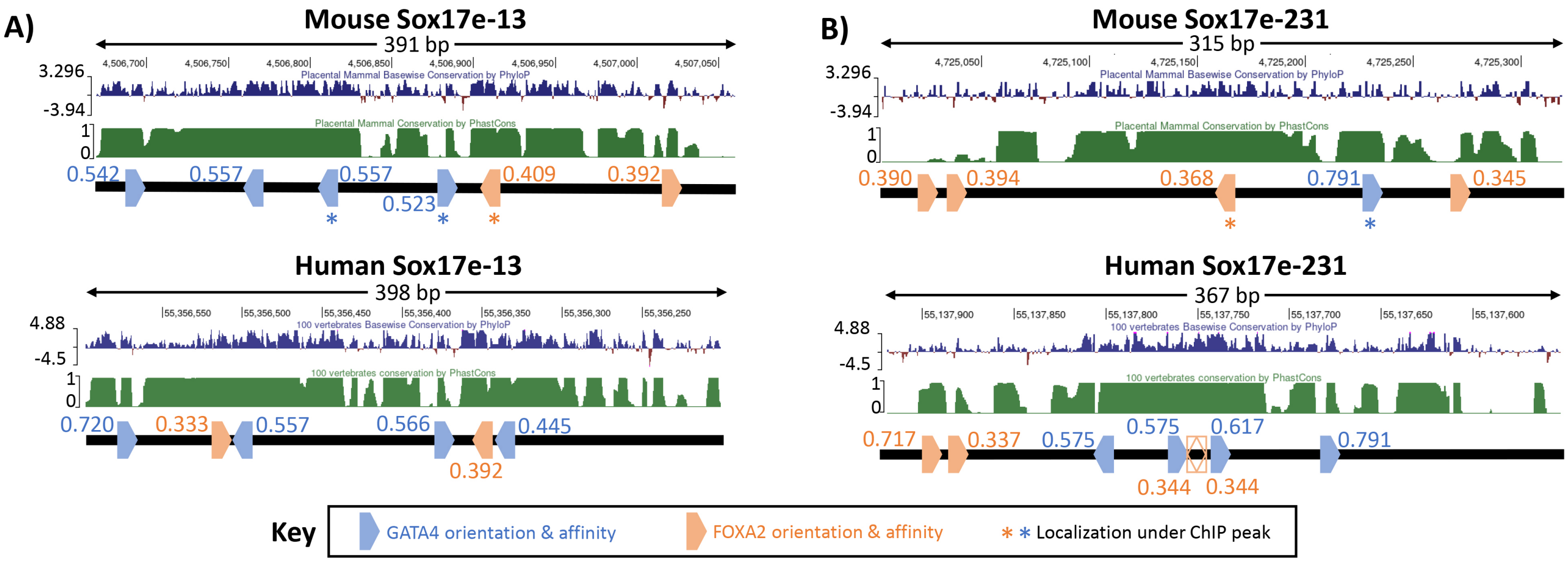

C)

Tissue	Publication	Published Enhancer Name	Published Human Coordinates (hg38)	Coordinates from Human to Mouse Liftover (mm10)	Coordinates From Our Study (mm10)	Do coordinates overlap with our study?
Human Endoderm	Luo et al. PMID: 37488417	SOX17e+2	chr8:54,443,606-54,444,178	chr1:4,506,539-4,507,092	Sox17e-13 chr1:4,506,670-4,507,060	Yes
Human Endoderm	Luo et al. PMID: 37488417	Sox17e+10	chr8:54,224,560-54,225,711	chr1:4,724,740-4,725,708	Sox17e-231 chr1:4,725,005-4,725,319	Yes
Human Pancreas	Kaplan et al. PMID: 39163202	SOX17e-233kb	chr8:54,223,608-54,228,661	chr1:4,722,334-4,725,758	Sox17e-231 chr1:4,725,005-4,725,319	Yes
Human Endoderm	Landshammer et al. PMID: 36719724	eSOX17	chr8:54,224,363-54,225,632	chr1:4,724,807-4,726,033	Sox17e-231 chr1:4,725,005-4,725,319	Yes

Fig. S7. Sox17e-13 and Sox17e-231 do not share similarities in FOXA2 and GATA4 motif enhancer grammar. A) Upper panel, mouse Sox17e-13 with PhyloP and PhastCons conservation tracks with FOXA2 and GATA4 sites identified from the JASPAR 2020 track within the UCSC genome browser. Viewing window mm10 chr1:4,506,670-4,507,060. Lower panel, human Sox17e-13 as identified via LiftOver from mm10 to hg19. PhyloP and PhastCons conservation tracks with FOXA2 and GATA4 sites identified from the JASPAR 2020 track within the UCSC genome browser. Viewing window hg19 chr8:55,356,201-55,356,598. B) Upper panel, mouse Sox17e-231 with PhyloP and PhastCons conservation tracks. FOXA2 and GATA4 sites identified from the JASPAR 2020 track within the UCSC genome browser. Viewing window mm10 chr1:4,725,005-4,725,319. Lower panel, human Sox17e-231 as identified via LiftOver from mm10 to hg19 with PhyloP and PhastCons conservation tracks. FOXA2 and GATA4 sites identified from the JASPAR 2020 track within the UCSC genome browser. Viewing window hg19 chr8:55,137,553-55,137,919. All relative TF-motif affinities were calculated using the UniPROBE database. C) Table of previously validated enhancers of *Sox17* which correspond to mouse Sox17e-13 or Sox17e-231.

A)

Species	Gallbladder?	Sox17e-13 ortholog	Sox17e-13 ortholog
Mouse *(Mus musculus,* mm10)	Yes	chr1:4506670-4507060	chr1:4725005-4725319
Squirrel *(Spermophilus tridecemlineatus,* speTri2)	Yes	JH393306:1235580-1235974	JH393306:1053413-1053772
Rat *(Rattus norvegicus,* rn7)	No	chr5:15006218-15006610	chr5:14792059-14792383
Alpaca *(Vicugna pacos,* vicPac2)	No	KB632479:2698566-2698964	KB632479:2573210-2573575

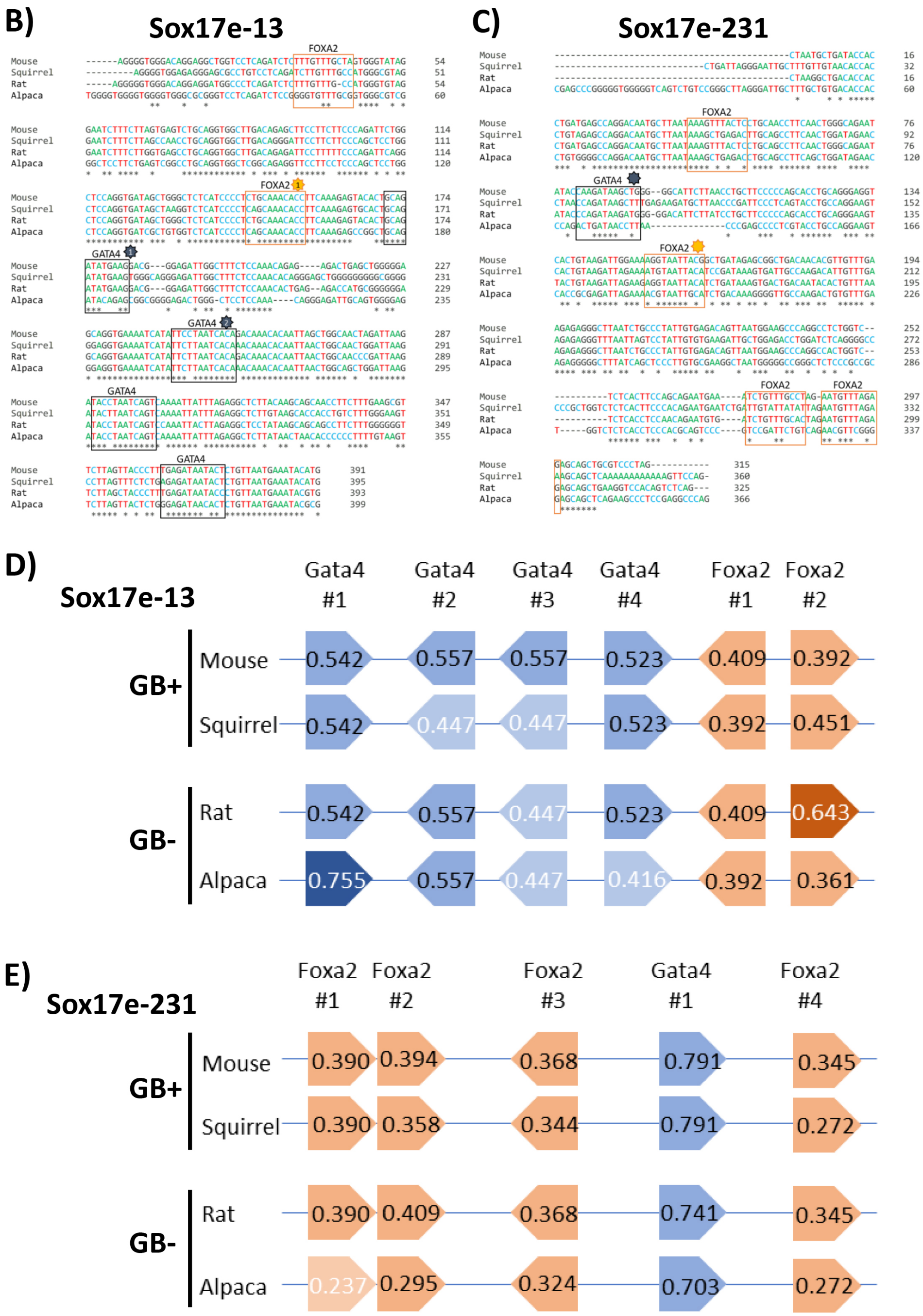

Development • Supplementary information

Fig. S8. Sox17e-13 and Sox17e-231 orthologs are similar in gallbladder present and absent species. A) Table describing gallbladder-present and gallbladder-absent species with the locations of their Sox17e-13 and Sox17e-231 orthologs. B) Multispecies alignment of Sox17e-13 and C) Sox17e-231 orthologs with FOXA2 and GATA4 sites marked in squares. An *denotes the base is conserved across all four species. Enhancer grammar analysis showingthe orientation and affinity of FOXA2 and GATA4 sites in D) Sox17e-13 and E) Sox17e-231 orthologs. Binding sites with a change equal to or greater than 0.010 are shown in white font and are shaded according to affinity increase or decrease.

Development • Supplementary information

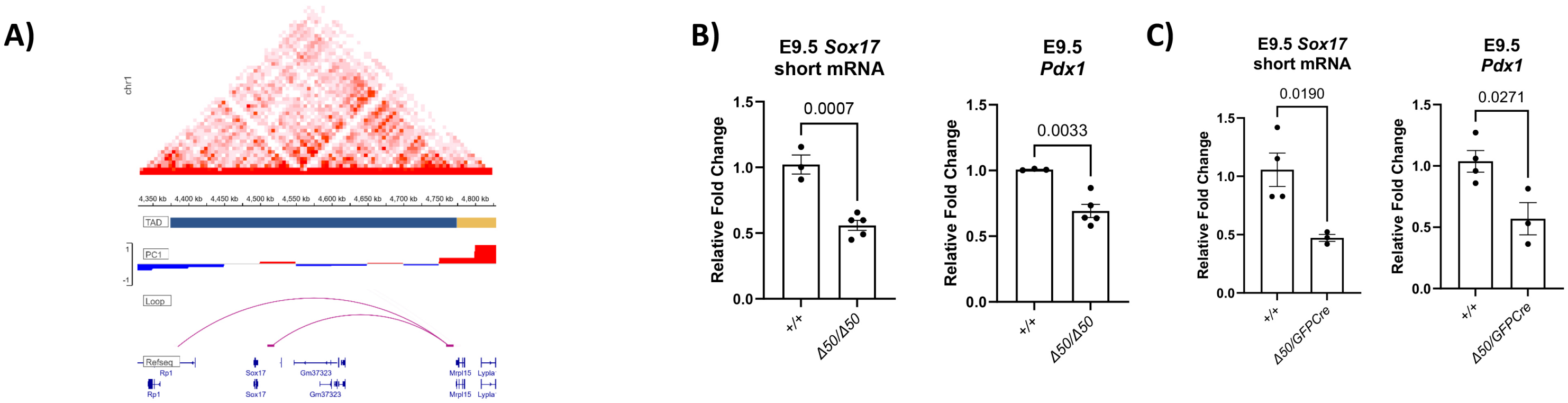

D)

Condition	Δe-13/Δe-13	Δe-231/Δe-231	Δe-13&e-231/Δe-13&e-231	Δe-13/GFPCre	Δe-231/GFPCre	Δe-13&-231/GFPCre
Alleles	$Sox17^{\Delta e\text{-}13}$	$Sox17^{\Delta e\text{-}231}$	$Sox17^{\Delta e\text{-}13\&e\text{-}231}$	$Sox17^{\Delta e\text{-}13}$<br>$Sox17^{GFPCre}$	$Sox17^{\Delta e\text{-}231}$<br>$Sox17^{GFPCre}$	$Sox17^{\Delta e\text{-}13\&e\text{-}231}$<br>$Sox17^{GFPCre}$
Effect on *Sox17* short mRNA form	E8.5: ns<br>E9.5: -31%<br>E10.5 -29%<br>E11.5: ns	E8.5: ns<br>E9.5: -26%<br>E10.5 -17%<br>E11.5: ns	E8.5: ns<br>E9.5: -48%<br>E10.5 -41%<br>E11.5: -40%	E9.5: -43%	E9.5: -49%	E9.5: -61%
Effect on E18.5 Pancreas Size	Decrease	Decrease	Decrease	Not surveyed	Not surveyed	Not surveyed
Effect on Adult Gallbladder Size	Increase	Increase	Decrease	Decrease or Agenesis	Decrease or Agenesis	Agenesis
Additional Phenotypes	N/A	Decreased *Trex17* expression	Decrease in *Trex17* expression; Abnormal gallbladder histology	Gallbladder agenesis with a normal cystic duct	Gallbladder agenesis with a normal cystic duct	Biliary atresia

- The $Sox17^{\Delta e\text{-}13}$ allele contains a 391 bp deletion of Sox17e-13.
- The $Sox17^{\Delta e\text{-}231}$ allele contains a 315 bp deletion of Sox17e-231.
- The $Sox17^{\Delta e\text{-}13\&e\text{-}231}$ allele contains a 369 bp deletion of Sox17e-13 and a 329 bp deletion of Sox17e-231.
- The $Sox17^{GFPCre}$ allele is functionally null. It expresses a GFPCre in lieu of *Sox17*.

Fig. S9. *Sox17* measurements for reconciliation and summary table. A) Hi-C heatmap near mouse *Sox17* in mESCs from GSE35156 visualized on the 3D Genome Browser 2.0. E9.5 RT-qPCR for *Sox17* short mRNA and *Pdx1* of wildtype, B) $Sox17^{\Delta 50/\Delta 50}$, and C) $Sox17^{\Delta 50/GFPCre}$ embryos. *Sox17* short mRNA measurements were performed as a control to validate Trinh & Finnel et al. *Development* (2025)/Figure 2B. *Pdx1* measurements were performed in $Sox17^{\Delta 50/\Delta 50}$ to compare to Trinh & Finnel et al. *Development* (2025)/Figure 1E and in $Sox17^{\Delta 50/GFPCre}$ embryos to serve as a control to match the findings of Trinh & Finnel et al. *Development* (2025)/Figure 6A. Each dot represents one biological replicate. Each biological replicate averages three technical replicates. Significance assessed by an unpaired *t*-test. All *p*-values shown. Data is mean ± s.e.m. C) Summary table of phenotypes and description of alleles used within this study. ns, non-significant.

Development • Supplementary information

Table S1. Characterization of the conserved regions within the likely mouse *Sox17* TAD.

Distance to Sox17 TSS2 promoter	Genomic Coordinates (mm10)	Length	Placental Cons (PhyloP60wayPlacental) Min	Placental Cons (PhyloP60wayPlacental) Max	Placental Cons (PhyloP60wayPlacental) Range	Placental Cons (PhyloP60wayPlacental) Mean	Placental Cons (PhyloP60wayPlacental) Variance	Placental Cons (PhyloP60wayPlacental) Standard Deviation	Placental Cons (PhastCons60way Placental) Min	Placental Cons (PhastCons60way Placental) Max	Placental Cons (PhastCons60way Placental) Range	Placental Cons (PhastCons60way Placental) Mean	Placental Cons (PhastCons60way Placental) Variance	Placental Cons (PhastCons60way Placental) Standard Deviation	Definitive Endoderm (DE) ATAC Seq Max (GSM3223248)	Definitive Endoderm (DE) H3K27ac Max (GSM3223297)	Definitive Endoderm (DE) H3K4me1 Max (GSM3223309)	Definitive Endoderm (DE) H3K4me3 Max (GSM3223313)	Definitive Endoderm (DE) FOXA2 Max (GSM3223327)	Definitive Endoderm (DE) GATA4 Max (GSM3223330)
+67 kb	chr1:4,426,773-4,427,222	450 bp	-4.635	2.784	7.419	0.38028	1.12877	1.06244	0	1	1	0.285502	0.157176	0.396454	0.78	1.06	1.61	0.39	4.1	1.65
+63 kb	chr1:4,430,103-4,430,644	542 bp	-3.591	3.073	6.664	1.04303	1.34417	1.15938	0	1	1	0.647848	0.171284	0.413864	0.47	0.82	0.46	0.39	1.49	0.33
+61 kb	chr1:4,432,450-4,432,952	503 bp	-3.77661	3.185	6.96161	1.35157	1.48707	1.21946	0	1	1	0.812464	0.119745	0.346042	0.94	0.71	1.83	0.58	1.49	0.99
+60 kb	chr1:4,433,756-4,433,964	209 bp	-1.35845	2.944	4.30245	0.716548	0.748637	0.865238	0	1	1	0.590476	0.144601	0.380264	0	0.71	0.69	0	0	0.33
+31 kb	chr1:4,461,736-4,462,315	580 bp	-4.128	2.799	6.927	0.627873	1.3656	1.16859	0	1	1	0.499611	0.22029	0.469351	0.62	0.59	0.54	0	0	0.99
+22 kb	chr1:4,471,328-4,471,723	396 bp	-4.81674	2.756	7.57274	0.419737	1.43991	1.19996	0	1	1	0.411815	0.199111	0.446219	0.78	2.24	2.22	1.45	3.35	0.66
-4 kb	chr1:4,497,943-4,498,265	323 bp	-2.15301	3.016	5.16901	0.421517	0.753082	0.867803	0	1	1	0.327076	0.160208	0.40026	1.25	2.59	2.22	10.31	0.74	1.98
-13 kb	chr1:4,506,670-4,507,060	391 bp	-2.79172	3.189	5.98072	1.23147	1.44485	1.20202	0	1	1	0.735868	0.160221	0.400276	8.58	1.88	1.68	2.22	46.54	19.85
-20 kb	chr1:4,513,961-4,514,346	386 bp	-3.086	3.08	6.166	1.28914	1.21351	1.1016	0	1	1	0.870752	0.0826021	0.287406	0.47	1.18	1.91	0.87	3.91	1.32
-31 kb	chr1:4,527,473-4,527,774	302 bp	-2.92887	2.562	5.49087	0.486692	0.913323	0.955679	0	1	1	0.430385	0.172633	0.415492	0.47	1.18	0.99	0.58	0	0.33
-40 kb	chr1:4,533,760-4,534,412	653 bp	-3.282	2.765	6.047	0.303355	0.882897	0.939626	0	1	1	0.276616	0.152554	0.390581	1.25	0.59	1.38	0.19	4.84	1.65
-43 kb	chr1:4,536,941-4,537,350	410 bp	-3.52801	3.005	6.53301	0.290321	1.39509	1.18114	0	1	1	0.2913	0.181343	0.425844	0.47	0.59	1.83	0.77	1.49	0.99
-50 kb	chr1:4,543,973-4,544,508	536 bp	-2.093	3.296	5.389	1.22565	1.49329	1.222	0	1	1	0.66199	0.185012	0.43013	3.59	4.83	4.28	2.02	10.8	2.32
-77 kb	chr1:4,570,905-4,571,060	156 bp	-3.05044	2.826	5.87644	0.664822	1.11336	1.05516	0	1	1	0.481476	0.222719	0.471931	0.16	0.59	1.68	0.96	1.12	1.98
-78 kb	chr1:4,571,623-4,572,587	965 bp	-4.74164	3.242	7.98364	0.975012	1.86137	1.36432	0	1	1	0.625907	0.191918	0.438085	17.62	6.48	1.61	39.31	2.42	3.97
-80 kb	chr1:4,573,972-4,574,432	461 bp	-3.85599	2.37633	6.23232	0.328566	0.961192	0.980404	0	1	1	0.348728	0.187204	0.43267	0.94	0.47	0.84	0.48	3.16	1.65
-90 kb	chr1:4,583,432-4,583,870	439 bp	-2.96892	3.141	6.10992	0.578483	1.08063	1.03953	0	1	1	0.390401	0.185512	0.430711	0.47	1.06	0.76	0.19	1.12	1.32
-105 kb	chr1:4,598,064-4,599,535	1,472 bp	-4.391	3.296	7.687	0.585649	1.30791	1.14364	0	1	1	0.441613	0.200402	0.447663	0.79	1.88	3.58	2.22	6.33	3.31
-109 kb	chr1:4,602,557-4,602,721	165 bp	-1.82275	3.235	5.05775	1.08776	1.26678	1.12551	0	1	1	0.782773	0.125827	0.354721	0.62	1.18	2.98	0.96	0.93	0.99
-112 kb	chr1:4,605,946-4,606,262	317 bp	-2.91969	2.625	5.54469	0.463638	1.1239	1.06014	0	1	1	0.333168	0.165997	0.407427	0.62	0.94	0.38	0	0	0.99
-120 kb	chr1:4,613,279-4,613,582	304 bp	-5.054	2.939	7.993	0.510917	1.57324	1.25429	0	1	1	0.347013	0.201535	0.448927	1.25	1.65	3.59	1.83	2.05	2.32
-123kb	chr1:4,616,441-4,617,167	727 bp	-2.72106	3.213	5.93406	0.553331	1.29558	1.13824	0	1	1	0.305419	0.176118	0.419664	0.47	0.82	1.83	0.1	0	3.31
-124 kb	chr1:4,617,401-4,617,995	595 bp	-2.96354	3.128	6.09154	0.991989	1.31447	1.1465	0	1	1	0.646794	0.182795	0.427546	0.31	0.94	1.45	0.1	0	1.98
-128 kb	chr1:4,622,380-4,622,823	444 bp	-2.04681	3.03699	5.0838	0.826873	1.21339	1.10154	0	1	1	0.564943	0.193462	0.439843	0.31	0.82	0.76	0.29	0.74	1.98
-130 kb	chr1:4,623,343-4,623,568	226 bp	-4.398	3.096	7.494	1.23486	1.41206	1.1883	0	1	1	0.874329	0.0772589	0.277955	0.31	1.41	0.99	0.29	1.3	0.33
-147 kb	chr1:4,641,030-4,641,327	298 bp	-2.025	2.88	4.905	0.772852	0.952002	0.975706	0	1	1	0.58524	0.194056	0.440518	1.56	1.06	2.29	0.96	5.58	1.32
-150 kb	chr1:4,644,531-4,645,124	594 bp	-3.372	2.989	6.361	0.431261	0.990212	0.995094	0	1	1	0.431785	0.194221	0.440705	3.12	0.59	3.06	2.02	21.41	4.96
-160 kb	chr1:4,653,780-4,654,326	547 bp	-3.648	3.175	6.823	1.05122	1.63228	1.27761	0	1	1	0.615627	0.191812	0.437963	0.94	1.77	1.76	1.83	2.05	8.27
-178 kb	chr1:4,671,844-4,672,618	775 bp	-3.425	2.993	6.418	0.745878	1.22574	1.10713	0	1	1	0.559919	0.193108	0.439441	0.47	2.59	2.83	1.16	2.61	1.32
-212 kb	chr1:4,706,066-4,706,826	761 bp	-3.353	3.154	6.507	0.85214	1.5091	1.22846	0	1	1	0.532908	0.211526	0.45992	0.78	2.12	1.15	0.1	1.12	0.33
-229 kb	chr1:4,722,776-4,722,877	102 bp	-1.1965	2.72239	3.9189	0.832977	0.792719	0.890348	0	1	1	0.719778	0.140835	0.37528	5.3	4.12	1.45	4.91	1.12	0.66
-231 kb	chr1:4,725,005-4,725,319	315 bp	-2.22519	2.995	5.22019	0.752254	1.11807	1.05739	0	1	1	0.588526	0.189891	0.435765	18.25	3.53	1.07	5.59	31.46	19.52
-246 kb	chr1:4,739,461-4,739,925	465 bp	-2.28055	2.962	5.24255	1.40836	1.38343	1.17619	0	1	1	0.826856	0.118809	0.344687	0.78	2.36	2.6	0.67	1.68	2.32
-252 kb	chr1:4,746,027-4,746,370	344 bp	-3.8342	3.09216	6.92636	0.306907	0.928946	0.963818	0	1	1	0.323865	0.176514	0.420135	0.62	0.71	0.38	0	0	0.66
-253 kb	chr1:4,746,692-4,747,135	444 bp	-4.256	3.154	7.41	0.736575	1.63077	1.27702	0	1	1	0.515074	0.196439	0.443215	0.31	2.12	1.07	0.29	1.68	0.99
-266 kb	chr1:4,759,229-4,759,505	277 bp	-2.0008	2.905	4.9058	1.28077	1.09882	1.04824	0	1	1	0.806788	0.126159	0.355189	0.16	0.82	0.69	0	1.49	1.65
-267 kb	chr1:4,760,369--4,760,813	445 bp	-2.747	2.998	5.745	0.977415	1.45163	1.20484	0	1	1	0.593966	0.197754	0.444696	1.25	2.59	1.15	0.67	1.86	1.98
-274 kb	chr1:4,767,810-4,768,066	257 bp	-3.20513	2.694	5.89913	0.856881	0.989058	0.994514	0	1	1	0.675787	0.167956	0.409824	0.47	2.37	1.45	0.48	0.93	0.33

Table S2. Chi-squared analysis for each mutant mouse line and compound heterozygous animals analyzed within this study.

*Sox17^e-13^* at P21	Observed	Expected	Value
+/+	11	12.5	0.18
$Sox17^{\Delta e\text{-}13/+}$	28	25	0.36
$Sox17^{\Delta e\text{-}13/\Delta e\text{-}13}$	11	12.5	0.18
Chi Squared Value	0.72		
Degrees of Freedom	2		
P Value	0.698		

*Sox17^e-13/GFPCre^* at P21	Observed	Expected	Value
+/+	7	8.75	0.35
$Sox17^{\Delta e\text{-}13/+}$	6	8.75	0.86
$Sox17^{GFPCre/+}$	6	8.75	0.86
$Sox17^{\Delta e\text{-}13/GFPCre}$	4	8.75	2.58
Chi Squared Value	4.66		
Degrees of Freedom	3		
P Value	0.198		

*Sox17^e-231^* at P21	Observed	Expected	Value
+/+	14	11.5	0.54
$Sox17^{\Delta e\text{-}231/+}$	20	23	0.39
$Sox17^{\Delta e\text{-}231/\Delta e\text{-}231}$	12	11.5	0.02
Chi Squared Value	0.96		
Degrees of Freedom	2		
P Value	0.619		

*Sox17^e-231/GFPCre^* at P21	Observed	Expected	Value
+/+	11	8.75	0.58
$Sox17^{\Delta e\text{-}231/+}$	4	8.75	2.58
$Sox17^{GFPCre/+}$	5	8.75	1.61
$Sox17^{\Delta e\text{-}231/GFPCre}$	9	8.75	0.01
Chi Squared Value	4.77		
Degrees of Freedom	3		
P Value	0.189		

*Sox17^e-13&e-231^* at P21	Observed	Expected	Value
+/+	13	12.5	0.02
$Sox17^{\Delta e\text{-}13\&\text{-}231/+}$	27	25	0.16
$Sox17^{\Delta e\text{-}13\&e\text{-}231/\Delta e\text{-}13\&e\text{-}231}$	10	12.5	0.5
Chi Squared Value	0.68		
Degrees of Freedom	2		
P Value	0.712		

*Sox17^e-13&e-231/GFPCre^* at P21	Observed	Expected	Value
+/+	17	8.75	7.78
$Sox17^{\Delta e\text{-}13\&e\text{-}231/+}$	10	8.75	0.18
$Sox17^{GFPCre/+}$	7	8.75	0.35
$Sox17^{\Delta e\text{-}13\&e\text{-}231/GFPCre}$	1	8.75	6.86
Chi Squared Value	15.17		
Degrees of Freedom	3		
P Value	0.002		

Development • Supplementary information

Table S3. Key resource table for reagents and datasets used in this study.

Alleles Used						
**Strain**	**Common Name**	**Background**	**Description of Mutation**	**Genmic Location (GRCm38 unless noted)**	**MGI Allele ID**	**Strain MGI Allele ID**
$Sox17^{tm2(EGFP/cre)Mgn}$	$Sox17^{GFPCre}$	CD1	Coding sequence replaced with eGFPCre	Chr1: 4561154-4567577 (GRCm39)	5468103	5468554
$Rr446^{em1Mgn}$	$Sox17^{\Delta e\text{-}13}$	CD1	391 bp deletion at -13 kb from the TSS2 promoter	Chr1:4,506,670-4,507,060	7522923	7840371
$Rr460^{em1Mgn}$	$Sox17^{\Delta e\text{-}231}$	CD1	315 bp deletion at -231 kbp from the TSS2 promoter	Chr1:4725005-4725318	7530991	7840374
$Rr606^{em1Mgn}$	$Sox17^{\mathbf{\Delta e\text{-}13}\,\&e\text{-}231}$	CD1	e-13 deletion within the e-13&e-231 dual deletion model	Chr1: 4576921-4577289 (GRCm39)	7863343	7863344
$Rr616^{em1Mgn}$	$Sox17^{\Delta e\text{-}13\&\mathbf{e\text{-}231}}$	CD1	e-231 deletion within the e-13&e-231 dual deletion model	Chr1: 4795219-4795547 (GRCm39)	8167012	7863344

Mutant Mouse Generation	
**Oligo**	**Sequence 5' --> 3'**
$Sox17^{\Delta e\text{-}13}$ 5' gRNA	TAACAGAGTATTATCTCAAA
$Sox17^{\Delta e\text{-}13}$ 3' gRNA	TAATCAAGGCACAGAACAAG
$Sox17^{\Delta e\text{-}13}$ ssDNA	CTCTTAGAGCTTTTATTGCTTTTACTGGAGCTTCATTGCATCGTTATAAGAGAAAGAATTCCTTTAAAAGGGGCACCTCTGTGAACTCGAGTGTTCTGTGCCTTGATTACTACTTCTGGAGAAGTAGTCACTTTGCAGTAGGACTCACAATTTAGTATTAAAAGTTTATTAAAGTAG
$Sox17^{\Delta e\text{-}231}$ 5' crRNA	GGAATACCCTGTAAGTGCTA
$Sox17^{\Delta e\text{-}231}$ 3' crRNA	ATCAGGTGGTATCAGCATTA
$Sox17^{\Delta e\text{-}231}$ ssDNA	GAGCTAAGCACTCAGCTAGTTGAGAGTGGCTGACATCAACTGGAATACCCTGTAAGTGCTCTCGAGGCTAATCAAAGTCCACCTCTGAATTGTGACATGAATGGACCTGCGTCACTCTGCTTCATC
$Sox17^{\Delta e\text{-}13\&e\text{-}231}$ e-13 5' crRNA	TAACAGAGTATTATCTCAAA
$Sox17^{\Delta e\text{-}13\&e\text{-}231}$ e-13 3' crRNA	TAATCAAGGCACAGAACAAG
$Sox17^{\Delta e\text{-}13\&e\text{-}231}$ e-231 5' gRNA	GGAATACCCTGTAAGTGCTA
$Sox17^{\Delta e\text{-}13\&e\text{-}231}$ e-231 3' gRNA	ATCAGGTGGTATCAGCATTA

PCR Primers			
**Primers**	**Purpose**	**Sequence 5' ---> 3'**	**Annealing Temp**
gtCR3-F	Genotyping PCR of Sox17e-13 mutation	CTGGAGCTTCATTGCATCGTTATAAGAG	65 C
gtCR3-R		GTGAGTCCTACTGCAAAGTGACTAC	
Sox17-CR4-Fwd1	Genotyping PCR of Sox17e-231 mutation	CTTTCATTCTAGTGAGTGGATCC	61 C
Sox17-CR4-Rev1		CATAAGACCACACTGAACAAACC	
Sox17-S2	Genotyping PCR of GFPCre	CAGAGGTATGCAGATCTCTGT	57 C
Sox17-3'UTR		CATTCTGGTCAACATGTAAGGT	
hT-REX17_Ex1_365bp_fwd	Detection of human TREX17	GAGACCACAGAGGGGTTGAG	66 C
hT-REX17_Ex1_365bp_rev		GAGCAGCAGGGAAACGAAAA	
mTREX17_F2	Detection of mouse TREX17	GAAACAGACTGTGGAACTGCCTAC	66 C
mTREX17_R2		GCAGCGATTACTATCCATTTCAGGG	

RT-qPCR Primers	
**Primers**	**Sequence 5' ---> 3'**
mSox17-a	GGTTAGGTCTACCAGGAGAGC
mSox17-c	CTTCTCTGCCAAGGTCAACG
mSox17-F1	CCAACACTCCTCCCAAAGTATCTATC
mSox17-R1	GAAATGACGATTGAAAACAGTAGC
mPdx1-F	CGGCTGAGCAAGCTAAGGTT
mPdx1-R	TGGAAGAAGCGCTCTCTTTGA
mPtf1a-F	GTAACCAGGCCCAGAAGG
mPtf1a-R	GTCCACACTTTAGCTGTACGG
mTrex17-F	CTTTGTCCTTGCTCTGATTTTG
mTrex17-R	TGTGTGTGGTGTGTCCTTG
hT-REX17_Ex1_365bp_fwd	GAGACCACAGAGGGGTTGAG
hT-REX17_Ex1_365bp_rev	GAGCAGCAGGGAAACGAAAA
mSox9-F	TGAAGAACGGACAAGCGGAG
mSox9-R	GATTGCCCAGAGTGCTCGC
mHNF4α-F	GTGAGCCTGGAGGATTACATC
mHNF4α-R	GGCATCGTGTTAGCAACAATG
mCd31-F	CTTGTCGCAGTATCAGAATTTC
mCd31-R	CCTATGGCATGTCCTTTTATG
mTie2-F	TTTCGGCATCAGACACAAGA
mTie2-R	CCGGCTTAGTTCTCTGTGGA
mKdr-F	GGACTGGCTTTGGCCCAAT
mKdr-R	CTTGCTGTCCCAGGAAATTCTG
mFlt4-F	CTGGCAAATGGTTACTCCATGA
mFlt4-R	ACAACCCGTGTGTCTTCACTG
mEphb4-F	CTACGTCTCTAACCTCCCATCT
mEphb4-R	GCTGGTCACCCTTTCTCTTT
mEfnb2-F	GAACTGGGAGCGGCTTGG
mEfnb2-R	TTGTCCGGGTAGAAATTTGGAGTT
mPdpn-F	CAACGAGATCAAGATGTGGACC
mPdpn-R	CATTAAGCCCTCCAGTAGCACC
mNr2f2-F	GGACCACATACGGATCTTCCAA
mNr2f2-R	ACATCAGACAGACCACAGGCAT
mLyve1-F	CAAAGCCTATTGCCACAACTC
mLyve1-R	CATGGAGGTCAAAGGTGGAG
hSox17-F	TGCCCTTTGTGTATAAGCCCGAGA
hSox17-R	GGGTAGTTGCAATAGTAGACCGCT

Development • Supplementary information

Antibodies		
**Antibody**	**Source**	**Identifier**
Goat anti-SOX17 (1:100 dilution for immunofluorescence staining (IF))	R&D Systems	Cat# AF1924, RRID:AB_355060
Guinea pig anti-PDX1 (1:1000 dilution for IF)	Christopher V.E. Wright's lab	N/A
Rabbit anti-HNF4A (C11F12) (1:500 dilution for IF)	Cell Signaling Technology	Cat# 3113, RRID:AB_2295208
Rabbit anti KI-67 (1:200 for IF)	Thermo Fisher Scientific	Cat# RM-9106-S1
Rabbit anti-cleaved caspase 3(C-CAS3, 1:400 dilution for IF)	Cell Signaling Technology	Cat #9661
Alexa Fluor 555 conjugated Donkey anti-Rabbit IgG (1:1000 dilution for IF)	Thermo Fisher Scientific	Cat# A31572, RRID:AB_162543
Alexa Fluor 488 conjugated Donkey anti-Goat IgG (1:1000 dilution for IF)	Thermo Fisher Scientific	Cat# A11055, RRID:AB_2534102
Alexa Fluor 647 conjugated Donkey anti-Guinea pig IgG (1:1000 dilution for IF)	Jackson Immunoresearch	Cat# 706-605-148, RRID: AB_2340476

GEO Datasets	
**GEO Dataset**	**Use**
GSM3223248	Mouse definitive endoderm ATAC-seq
GSM3223297	Mouse definitive endoderm H3K27ac ChIP-seq
GSM3223309	Mouse definitive endoderm H3K4me1 ChIP-seq
GSM3223313	Mouse definitive endoderm H3K4me3 ChIP-seq
GSM3223327	Mouse definitive endoderm FOXA2 ChIP-seq
GSM3223330	Mouse definitive endoderm GATA4 ChIP-seq
GSM5509516	Mouse embryonic stem cells CTCF ChIP-seq
GSE207692	Mouse liver CTCF ChIP-seq
GSE96107	Mouse neural progenitor cells CTCF ChIP-seq
GSM4403033	Mouse hematopoietic stems cells CTCF ChIP-seq
GSM1505620	Human HUES64 hESC CTCF ChIP-seq
GSM1505625	Human HUES64 definitive endoderm CTCF ChIP-seq

Development • Supplementary information
