# Supplementary material for: Two distributed enhancers of Sox17 additively regulate the developmental fate of bipotential ventral pancreato-biliary progenitor cells
Source: Development. 2026 Sep 18;153(18):dev205751. doi: 10.1242/dev.205751 (PMC13680832; doi:10.1242/dev.205751)
Supplement: Supplementary file 2 [file develop-153-205751_review_history.pdf]

Development

Two distributed enhancers of Sox17 additively regulate the developmental fate of bipotential ventral pancreato-biliary progenitor cells

Ryan R. Finnel, Jessica R. Musselman, Margaret E. McFadden, Isabella M. Silvestri, Jennifer Skelton, Leesa L. Sampson, Anna B. Osipovich, Christopher V. E. Wright and Mark A. Magnuson

DOI: 10.1242/dev.205751

Editor: James M Wells

Review timeline

Original submission:	20 April 2026
Editorial decision:	23 May 2026
First revision received:	11 August 2026
Accepted:	19 August 2026

Original submission

First decision letter

MS ID#: dev.205751

MS TITLE: Two distributed enhancers of Sox17 additively regulate the developmental fate of bipotential ventral pancreato-biliary progenitor cells

AUTHORS: Ryan R. Finnel, Jessica R. Musselman, Margaret E. McFadden, Isabella M. Silvestri, Jennifer Skelton, Leesa L. Sampson, Anna B. Osipovich, Christopher V. E. Wright and Mark A. Magnuson

Dear Dr Magnuson,

I have now received all the referees reports on the above manuscript, and have reached a decision. The referees' comments are appended below.

The overall evaluation is positive and we would like to publish a revised manuscript in Development, provided that the referees' comments can be satisfactorily addressed. Please attend to all of the reviewers' comments in your revised manuscript and detail them in your point-by-point response. If you do not agree with any of their criticisms or suggestions explain clearly why this is so. If it would be helpful, you are welcome to contact us to discuss your revision in greater detail. Please send us a point-by-point response indicating your plans for addressing the referees' comments, and we will look over this and provide further guidance.

Reviewer 1

SUMMARY OF THE ADVANCE MADE IN THIS PAPER AND ITS POTENTIAL SIGNIFICANCE TO THE FIELD

The Finnel et al. study identified two murine Sox17 enhancers, which show cross-species conservation with two previously characterized SOX17 enhancers in humans. The authors conducted careful deletion (single and double deletions) experiments and analyzed the impact of enhancer deletion on Sox17 expression, as well as subsequent dosage sensitive regulation of the fate of the bipotential ventral pancreato-biliary cells.

© 2026. Published by The Company of Biologists under the terms of the Creative Commons Attribution License (https://creativecommons.org/licenses/by/4.0/).

Overall, the study was carefully executed. They demonstrate elegantly how enhancers exquisitely regulate gene dosage, and how different dosages of transcription factors lead to different developmental outcomes. The findings are of general interest to the developmental biology field.

SUGGESTIONS TO AUTHORS

Main points:
1. The title says that "... enhancers of Sox17 additively regulate …". While it seems correct based on data in some figures, it's not correct in others. For instance, in most later figures, the double deletion appears to have the same impact as the single deletion. Please revise and/or discuss this.

2. Figure 1A
The authors appear to rely primarily on CTCF binding patterns to define the TAD. However, CTCF occupancy alone is insufficient to delineate TAD structure, which is typically defined from chromatin contact maps (e.g., Hi-C or Micro-C) using established boundary-calling approaches. The authors should therefore support their TAD assignment using chromatin contact data or reference previously published TAD annotations (in a matched cellular context if possible). Including a Hi-C map at this locus would also provide visual support for the claim.

In addition, the CTCF sites at the proposed boundary appear to be oriented in the same direction. Under the loop extrusion model, convergent motif orientation is commonly associated with stable loop anchors and boundary formation. While not strictly required, the absence of convergent orientation suggests that the current assignment may be overinterpreted.

3. Figure 1D
The authors identify 38 conserved regions within the Sox17 TAD and prioritize two regions of interest based on epigenetic features and TF binding profiles. While Figure 1C and 1D provide useful information and a clear schematic summary, they should complement these with representative genome browser tracks (e.g., IGV or UCSC) showing the underlying data, including ATAC-seq, H3K27ac, FOXA2, GATA4, and CTCF ChIP-seq signals. This would allow readers to directly assess the primary evidence supporting the prioritization.

4. Figure 2.
The analysis of the deletion effects on SOX17 expression is very informative. However, one question is that the SOX17 expression itself also changes over time. While comparison of gene expression changes to the wild-type control makes sense, it would also be informative to see the changes of SOX17 expression over time (and how the impact of enhancer deletion correlates with temporal changes of SOX17 expression.

Related to this, what are the relative expression levels of the SOX17 long versus short isoform? Given that the enhancer perturbations only affect the short isoform, the relative expression is important as it will affect how the deletions impact on SOX17 protein abundance (which is not directly quantified in the study; immunostaining is available but not quantitative).

5. The Sox17 dosage relationship with the developmental outcome is a bit confusing. Previously, the Spence et al paper suggested that strong SOX17 loss of function leads to be biliary to pancreas conversion. In the 2025 Development paper from this group, they show that biliary fate is more sensitive to Sox17 dosage reduction, and pancreas is less sensitive (lost only when there's a severe loss of Sox17). In this new paper, the observations seem to point to a higher sensitivity of the pancreas to Sox17 dosage reduction (with single enhancer deletion causing a decrease in the PDX1+ domain and an expansion of the biliary domain), and a small reduction of Sox17 actually leads to an increase of the biliary fate. While this description may have over simplified things (as there are temporal differences in the perturbation / readout), it would be great that the authors reconcile these findings and/or clarify.

6. Despite the stronger impact of the double deletion (compared to the singles) on Sox17 expression in Figure 2, their impact on pancreatic development (in figure 4) appeared comparable to the single deletion. Please comment.

© 2026. Published by The Company of Biologists under the terms of the Creative Commons Attribution License (https://creativecommons.org/licenses/by/4.0/).

7. Figure 5 and Figure 6
The second half of Figure 5 (on Sox17Δe-13&e-231/GFPCre) should be together with Figure 6. It's confusing to read in their current order.

8. The relationship between the enhancers and the TREX17 as a lncRNA with a function in Sox17 regulation and pancreatic differentiation should be written with more clarity.

For instance, the following sentence is not accurate:
"Deletion of TREX17 was reported to prevent formation of pancreatic progenitor cells from hESCs (Landshammer et al., 2023)."

The Landshammer did dCas9-mediated knockdown of TREX17, and show a reduction of PDX1+ pancreatic progenitor cells in their differentiation assay, which could be consistent with either TREX17 (lncRNA) regulate SOX17 expression and downstream differentiation events, or TREX17 promoter reside in/near an enhancer region for SOX17 and perturbation of the enhancer affects SOX17 expression and subsequent differentiation (consistent with the current study). Importantly, Landshammer et al also inserted a termination signal to eliminate the lncRNA and show that the TREX17 transcript itself is dispensable for SOX17 activation. These findings are consistent with the authors' interpretation that "Trex17 expression has little impact on pancreas development in mice". Therefore, the authors could synthesize the previous finding better and discuss their new findings accordingly.

Additional points:
1. Please re-write the following paragraph. The three sentences are repeating each other.

"As the cell-state transition towards unipotency is further delayed but not eliminated when both enhancers are deleted, Sox17e-13 and Sox17e-231 may function to maintain the developmental tempo required for proper lineage allocation arising from the binary cell-fate decision occurring in ventral pancreato-biliary progenitors. However, the dual deletion of Sox17e-13 and Sox17e- 231 did not eliminate the bipotent-to-unipotent transition, suggesting that the enhancers are not required for this developmental cell-fate transition to occur, but are required to maintain the proper developmental tempo of the cell-fate transition. Taken together, we conclude that Sox17e-13 and Sox17e-231 function within a two-day period to promote Sox17 expression in ventral pancreato-biliary progenitor cells, ensuring that Sox17 expression reaches critical thresholds necessary to maintain the developmental tempo required for proper progenitor cell specification and lineage allocation."

2. Figure 2 and 3 (and other similar figures). Please clarify what each dot means in the bar graphs. Do individual dots correspond to individual embryos (biological replicates) or do they correspond to technical replicates?

3. Figure 3B and other figures with similar styles, please make sure that the bar graphs are aligned (matching height of the y axis). For instance, the middle panel in Figure 3B is higher compared to the others. This is important as the readers may want to compare between different genotypes (double versus single deletion for example). In addition, the authors can consider adding a dashed line at 0.5 to aid the visualization and comparisons.

4. "To determine if the Sox17e-13 and Sox17e-231 enhancers promote EHBD formation, we had to first reduce Sox17 dosage to bypass the gallbladder hypoplasia phenotype in Fig. 5A."
This sentence is confusing to read. Perhaps the authors meant to bypass the gallbladder "hyperplasia" phenotype in Fig. 5?

Reviewer 2

SUMMARY OF THE ADVANCE MADE IN THIS PAPER AND ITS POTENTIAL SIGNIFICANCE TO THE FIELD

This manuscript by Finnel et al. investigates the roles of two distributed enhancers, Sox17e-13 and Sox17e-231, in regulating Sox17 expression during hepato-pancreato-biliary development. Using elegant CRISPR-mediated enhancer deletions, the authors demonstrate additive and temporally dependent functions of these enhancers in ventral pancreato-biliary lineage specification. A

© 2026. Published by The Company of Biologists under the terms of the Creative Commons Attribution License (https://creativecommons.org/licenses/by/4.0/).

particularly interesting aspect of this study is the proposal that enhancer dosage regulates the developmental tempo of ventral pancreato-biliary fate resolution, rather than simply binary lineage specification. The study provides important insights into Sox17 dosage-dependent developmental regulation and enhancer function in vivo. The experiments are carefully performed, and the manuscript is overall well written and of broad interest to the developmental biology field.
I have several minor comments that may help improve the clarity and conceptual framing of the manuscript.

SUGGESTIONS TO AUTHORS

1. Recovery of Sox17 short mRNA expression at E11.5
In Sox17Δe-13/Δe-13 and Sox17Δe-231/Δe-231 embryos, Sox17 short mRNA expression is reduced at E9.5 and E10.5, but appears to recover to wild-type levels by E11.5. In contrast, Sox17Δe-13&e-231/Δe-13&e-231 embryos continue to show reduced expression at E11.5. Additional discussion or explanation regarding the apparent recovery of Sox17 expression in the single-enhancer deletion embryos would strengthen the interpretation of these findings.
2. Schematic illustration of the Sox17 gene structure
The manuscript describes two alternative promoters within Sox17, where TSS2-derived short mRNA is associated with endodermal expression and TSS1-derived long mRNA is associated with endothelial expression. However, it is somewhat difficult for readers to fully understand the genomic organization solely from the text. It would be helpful to include a schematic diagram showing the Sox17 gene structure, the positions of TSS1 and TSS2, the structures of the short and long transcripts, and the locations of the analyzed enhancer regions.
3. Quantification of cell populations in Fig. 3
The immunostaining images in Fig. 3 suggest that the morphology and overall size of the ventral pancreato-biliary domain differ among genotypes. Therefore, percentage-based quantification alone may not fully capture the biologically relevant changes in progenitor allocation. In addition, the denominator used for calculating the percentages of SOX17+, PDX1+, and SOX17+/PDX1+ cells should be clearly stated in the figure legend and/or Methods section.
4. Ki67 signals in Supplementary Fig. 3B
In Supplementary Fig. 3B, the Ki67 signal in Sox17Δe-13/Δe-13 embryos appears somewhat stronger than in the other genotypes. The authors should clarify whether this reflects a biological difference or is due to staining or imaging conditions.
5. Opposite phenotypes between single- and double-enhancer deletions
The finding that single-enhancer deletions lead to enlarged gallbladders whereas double-enhancer deletions result in smaller gallbladders is particularly interesting. Additional discussion regarding the possible mechanisms underlying these apparently opposite phenotypes would further strengthen the manuscript.
6. Significance of experiments using the Sox17GFPCre/+ background
Because Sox17GFPCre/+ mice already exhibit abnormalities in biliary development, the rationale for using this sensitized background could be explained more explicitly. It would also be helpful to clarify how the compound heterozygous phenotypes extend beyond those observed in Sox17GFPCre/+ animals alone.
7. Relationship to the concept of shadow enhancers
The manuscript would benefit from a slightly deeper discussion of how Sox17e-13 and Sox17e-231 relate to the classical concept of "shadow enhancers," particularly because deletion of either enhancer alone already produces measurable phenotypes.
8. Conservation of these regulatory regions in species lacking a gallbladder
Since the study demonstrates important roles of Sox17e-13 and Sox17e-231 in gallbladder and extrahepatic bile duct development, it would be interesting to discuss whether these regulatory regions are conserved in animal species lacking a gallbladder, such as rats.

<u>Reviewer 3</u>

In this study, Finnel et al use existing genomic datasets to identify two enhancers of Sox17 (Sox17e-13 and Sox17e-231), and then use mouse genetics to assess how loss of one or both of these enhancers, either alone or as compound mutants with heterozygous deletion of Sox17, affects Sox17 dosage and corresponding development of the hepato-pancreato-biliary (HPB) system. The main claims are that 1) Sox17e-13 and Sox17e-231 regulate Sox17 expression in an additive and

© 2026. Published by The Company of Biologists under the terms of the Creative Commons Attribution License (https://creativecommons.org/licenses/by/4.0/).

time-dependent fashion, 2) fate restriction of bipotent pancreato-biliary cells is Sox17 dosage-dependent, and that 3) Sox17 dosage dependency varies by developmental stage.
This is a meticulous, well-executed study of an important transcription factor with a clinically relevant phenotype. The study also has some broader importance, as it demonstrates how precise TF dosage thresholds can depend exquisitely on development stage and at the scale of phenotype (i.e. molecular vs cellular vs gross morphological). I broadly agree with the three main claims made, but have four related comments that I believe could help strengthen the claims and make the authors' proposed models more accessible to readers:

1) Measuring Sox17 levels in the various mutants: the authors do this by whole-embryo qPCR, which is reasonable given the limited material, but this raises the question of whether the measured Sox17 levels are truly representing the levels in the (presumably small) population of pancreato-biliary progenitors (or their fate-restricted progeny) at E9.5-E11.5. Is Sox17 expressed elsewhere in the embryo at these timepoints, and if so would this change the Sox17 dosages measured in Figure 2D? Perhaps the authors could re-analyze existing whole-embryo scRNA-seq to test this, or use IF or RNA FISH at E9.5.
2) Differing dependencies of HPB phenotypes on Sox17 dosage would be easier for readers to visualize/understand if the authors could plot, for any given phenotype, the phenotype as a function of measured Sox17 dosage (% of wild-type), i.e. the levels shown in Figure 2D or Figure 6A. This would be a more intuitive way of visualizing what the different Sox17 dosage thresholds are without having to refer back to previous panels to understand what % dosage each genotype corresponds to.
3) There are a few places where it seems like the dosage-dependencies don't line up, but this may be due to point 2. For example, when considering gallbladder weight in both the enhancer-only deletions (Figure 5B) and sensitized background (Figure 6C), it would appear that Δe-13/GFPCre has a significantly more severe phenotype than Δe-13&e-231/Δe-13&e-231, despite the two genotypes having approximately the same dosage reduction of ~50%. This could be clarified by placing the data on the same dosage % scale as suggested above.
4) In the authors Figure 7B model, they only rationalize/consider the effects of the enhancer-alone deletions. What about the phenotypes of compound heterozygous deletions (i.e. Figure 6)? How do these fit into the model? This relates to my broader observation that the enhancer-only deletions and the senitizing background deletions are treated as two distinct experiments, where in reality they should be considered as exploring different parts of the Sox17 dosage-response curve. For example, the authors demonstrate a severe reduction in EHBD Lumen Area in the Δe-13&e-231/GFPCre mutant (~60% Sox17 reduction) - is it a very sharp transition to this phenotype? This could be answered by quantifying EHBD Lumen Area some of the other mutants (i.e. Δe-13/Δe-13, Δe-231/Δe-231, Δe-13&e-231/Δe-13&e-231, GFPCre/+, Δe-13/GFPCre, Δe-231/GFPCre)

First revision

Author response to reviewers' comments

We thank all reviewers for their informed and helpful comments.

Reviewer 1:
1. The title says that "... enhancers of Sox17 additively regulate ...". While it seems correct based on data in some figures, it's not correct in others. For instance, in most later figures, the double deletion appears to have the same impact as the single deletion. Please revise and/or discuss this.

Our assertion that the enhancers function in an additive manner is based on measurements of *Sox17* mRNA where we found that the single deletions reduced expression by 29% and 17% and the double deletion reduced expression by 41% in whole embryos at E10.5. We prefer to retain the word “additive” as it succinctly summarizes the bulk of our findings.

2. Figure 1A. The authors appear to rely primarily on CTCF binding patterns to define the TAD. However, CTCF occupancy alone is insufficient to delineate TAD structure, which is typically

© 2026. Published by The Company of Biologists under the terms of the Creative Commons Attribution License (https://creativecommons.org/licenses/by/4.0/).

defined from chromatin contact maps (e.g., Hi-C or Micro-C) using established boundary-calling approaches. The authors should therefore support their TAD assignment using chromatin contact data or reference previously published TAD annotations (in a matched cellular context if possible). Including a Hi-C map at this locus would also provide visual support for the claim.

In addition, the CTCF sites at the proposed boundary appear to be oriented in the same direction. Under the loop extrusion model, convergent motif orientation is commonly associated with stable loop anchors and boundary formation. While not strictly required, the absence of convergent orientation suggests that the current assignment may be overinterpreted.

We agree our studies do not definitively define the murine *Sox17* TAD. That was not the principal goal of our study, nor do our conclusions depend upon it. The two CTCF binding sites that are conserved between humans and mice, and which in human are tandemly oriented, provided a logical boundary around mouse *Sox17* to identify putative enhancers, with this region being found to contain 38 conserved regions of at least 100 bp, two of which were studied in this report. We agree it still remains arguable whether the two CTCF sites truly define the murine *Sox17* TAD but published Hi-C data visualized on the 3D Genome Browser 2.0 (Dixon et al., 2012) (GSE35156, mESCs) supports this notion and suggest a loop from mm10 chr1:4,380,001-4,390,000 to chr1:4,760,001-4,770,000. While the data is limited by a 10 kb resolution, their called centromeric boundary overlaps with where CTCF binding occurs. However, the called telomeric boundary and our CTCF-defined boundary do not overlap. Given these data limitations, we have modified our discussion to temper our conclusions about the murine *Sox17* TAD.

NOTE: Figure provided for reviewer has been removed. It showed Dixon et al., 2012.

Dixon, J. R., Selvaraj, S., Yue, F., Kim, A., Li, Y., Shen, Y., Hu, M., Liu, J. S. and Ren, B. (2012) Topological domains in mammalian genomes identified by analysis of chromatin interactions. Nature 485(7398): 376-80. doi: https://doi.org/10.1038/nature11082

3. Figure 1D. The authors identify 38 conserved regions within the Sox17 TAD and prioritize two regions of interest based on epigenetic features and TF binding profiles. While Figure 1C and 1D provide useful information and a clear schematic summary, they should complement these with representative genome browser tracks (e.g., IGV or UCSC) showing the underlying data, including ATAC-seq, H3K27ac, FOXA2, GATA4, and CTCF ChIP-seq signals. This would allow readers to directly assess the primary evidence supporting the prioritization.

We now show the requested data in Figure 1E and have updated both the figure caption and related text. The TAD-wide view of the epigenetic and TF binding data was originally shown in Figure S1D. To improve visibility in the larger genomic context, we have highlighted Sox17e-13 and Sox17e-231 in Figure S1D.

4. Figure 2. The analysis of the deletion effects on SOX17 expression is very informative. However, one question is that the SOX17 expression itself also changes over time. While comparison of gene expression changes to the wild-type control makes sense, it would also be informative to see the changes of SOX17 expression over time (and how the impact of enhancer deletion correlates with temporal changes of SOX17 expression. Related to this, what are the relative expression levels of the SOX17 long versus short isoform? Given that the enhancer perturbations only affect the short isoform, the relative expression is important as it will affect how the deletions impact on SOX17 protein abundance (which is not directly quantified in the study; immunostaining is available but not quantitative).

Given that both enhancers stimulate *Sox17* expression and that we've previously described the importance of a positive autoregulatory loop for *Sox17* (Trinh et al., 2025), we predict that expression of *Sox17* short mRNA would indeed change over time. We attempted to normalize our relative *Sox17* expression measurements, but this was complicated by the massive embryonic growth that occurs between E8.5 and E11.5. Also, the number of biliary progenitor cells changes greatly over this timeframe, adding an additional confounding variable.

© 2026. Published by The Company of Biologists under the terms of the Creative Commons Attribution License (https://creativecommons.org/licenses/by/4.0/).

Due to the massive expansion of the vasculature after E10.5, the relative expression levels of the long and short *Sox17* mRNAs do indeed change, with the TSS2-derived mRNA becoming progressively less abundant when viewed in a relative manner. On account of this, and since our focus has been on expression from TSS2 whose functional impairment affects hepato-pancreato-biliary development, we used promoter-specific primer pairs that independently assess the TSS1 and TSS2 derived mRNAs. Previously we reported that deletion of the TSS2 promoter causes embryonic lethality mainly by disrupting hepato-pancreato-biliary development, whereas deletion of the TSS1 promoter has a much weaker phenotype, only affecting some lymphatic genes (Trinh et al., 2022).

5. The Sox17 dosage relationship with the developmental outcome is a bit confusing. Previously, the Spence et al paper suggested that strong SOX17 loss of function leads to be biliary to pancreas conversion. In the 2025 Development paper from this group, they show that biliary fate is more sensitive to Sox17 dosage reduction, and pancreas is less sensitive (lost only when there's a severe loss of Sox17). In this new paper, the observations seem to point to a higher sensitivity of the pancreas to Sox17 dosage reduction (with single enhancer deletion causing a decrease in the PDX1+ domain and an expansion of the biliary domain), and a small reduction of Sox17 actually leads to an increase of the biliary fate. While this description may have over simplified things (as there are temporal differences in the perturbation / readout), it would be great that the authors reconcile these findings and/or clarify.

The reviewer makes a critically important observation. Prompted by this comment, we repeated the RT-qPCR of *Pdx1* and *Sox17* short mRNA expression in *Sox17*$^{\Delta 50/\Delta 50}$ and *Sox17*$^{\Delta 50/GFPCre}$ embryos, as shown below. The measurements, which were performed by a different individual using different RNA samples, contradict the data shown in Figure 1 of Trinh & Finnel et al. published in *Development*, 2025. We show the new data in Supplemental Figure 9 and have tried to reconcile the differences in the discussion.

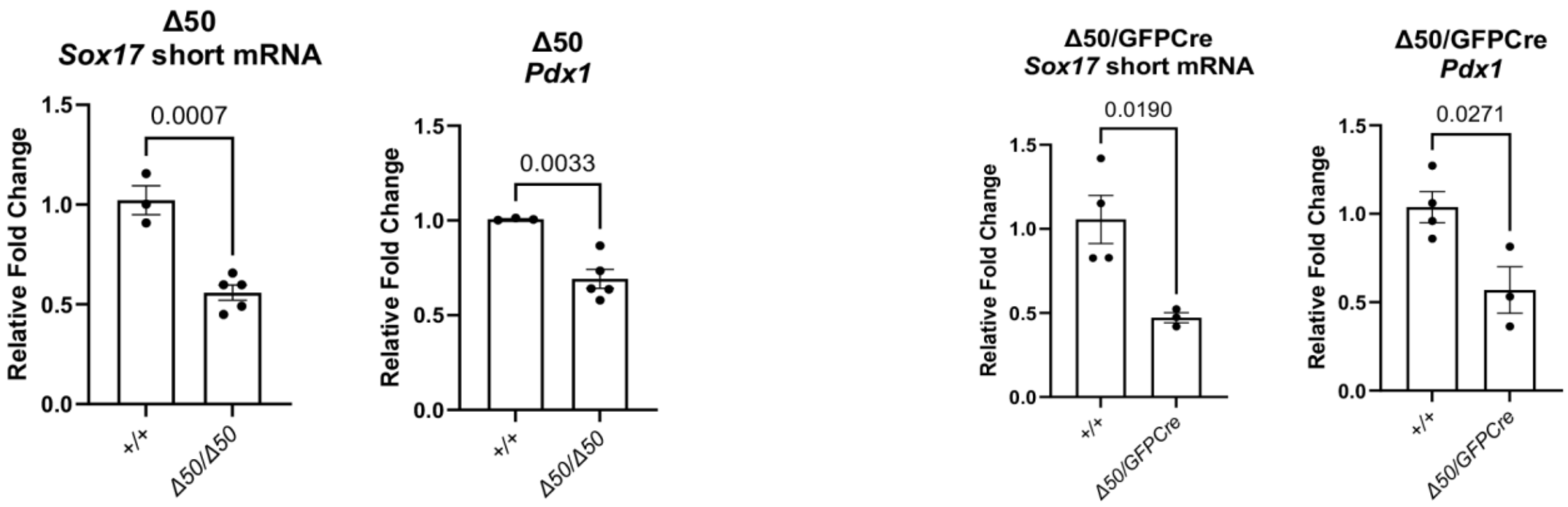

6. Despite the stronger impact of the double deletion (compared to the singles) on Sox17 expression in Figure 2, their impact on pancreatic development (in figure 4) appeared comparable to the single deletion. Please comment.

We agree that the pancreas weight of the entire organ at E18.5 is not significantly different between the single and dual enhancer deletion mutants (Figure 4G) and we hypothesize this is likely related to three factors. First, the dorsal pancreatic bud contributes more mass to the mature pancreas than the ventral pancreatic bud (Li et al., 2018). Second, *Sox17* is specifically involved in the development of the ventral, but not dorsal, pancreatic bud (Pan and Wright, 2011). Third, distinguishing dorsally and ventrally derived pancreas is difficult because of the lack of distinct markers. Given the lack of pancreatic-bud specific markers and differential contributions of the dorsal and ventral pancreatic buds, our ability to precisely measure changes arising from only the ventral bud within the whole organ is limited.

7. Figure 5 and Figure 6. The second half of Figure 5 (on Sox17Δe-13&e-231/GFPCre) should be together with Figure 6. It's confusing to read in their current order.

Thank you for pointing out this conceptual difficulty. We have adjusted our rationale to focus on why we included the *Sox17*$^{\Delta e\text{-}13\&e\text{-}231/GFPCre}$ in Figure 5.

© 2026. Published by The Company of Biologists under the terms of the Creative Commons Attribution License (https://creativecommons.org/licenses/by/4.0/).

8. The relationship between the enhancers and the TREX17 as a lncRNA with a function in Sox17 regulation and pancreatic differentiation should be written with more clarity. For instance, the following sentence is not accurate: “Deletion of TREX17 was reported to prevent formation of pancreatic progenitor cells from hESCs (Landshammer et al., 2023).” The Landshammer paper did dCas9-mediated knockdown of TREX17, and showed a reduction of PDX1+ pancreatic progenitor cells in their differentiation assay, which could be consistent with either TREX17 (lncRNA) regulate SOX17 expression and downstream differentiation events, or TREX17 promoter resides in/near an enhancer region for SOX17 and perturbation of the enhancer affects SOX17 expression and subsequent differentiation (consistent with the current study). Importantly, Landshammer et al also inserted a termination signal to eliminate the lncRNA and show that the TREX17 transcript itself is dispensable for SOX17 activation. These findings are consistent with the authors’ interpretation that “Trex17 expression has little impact on pancreas development in mice”. Therefore, the authors could synthesize the previous finding better and discuss their new findings accordingly.

Thank you again for these helpful and important comments. We altered our text to more clearly point out that TREX17 was previously studied by dCas9-mediated knockdown. Our revised text also more clearly states that TREX17 is dispensable for *Sox17* activation and knockdown results in diminished pancreatic progenitor differentiation. We have also elaborated on the potential interplay & shared regulatory mechanisms between *Sox17* and *Trex17*.

Additional points:

1. Please re-write the following paragraph. The three sentences are repeating each other. “As the cell-state transition towards unipotency is further delayed but not eliminated when both enhancers are deleted, Sox17e-13 and Sox17e-231 may function to maintain the developmental tempo required for proper lineage allocation arising from the binary cell-fate decision occurring in ventral pancreato-biliary progenitors. However, the dual deletion of Sox17e-13 and Sox17e- 231 did not eliminate the bipotent-to-unipotent transition, suggesting that the enhancers are not required for this developmental cell-fate transition to occur, but are required to maintain the proper developmental tempo of the cell-fate transition. Taken together, we conclude that Sox17e-13 and Sox17e-231 function within a two-day period to promote Sox17 expression in ventral pancreato-biliary progenitor cells, ensuring that Sox17 expression reaches critical thresholds necessary to maintain the developmental tempo required for proper progenitor cell specification and lineage allocation.”

We agree that the prior sentences were duplicative and have condensed the entire section into two sentences.

2. Figure 2 and 3 (and other similar figures). Please clarify what each dot means in the bar graphs. Do individual dots correspond to individual embryos (biological replicates) or do they correspond to technical replicates?

Each dot represents one biological replicate (one embryo/organ). We changed the legend of Figure 2, Figure 3, Figure 4, Figure 5, Figure 6, and Figure S2, to clarify what each dot means within the bar graphs.

3. Figure 3B and other figures with similar styles, please make sure that the bar graphs are aligned (matching height of the y axis). For instance, the middle panel in Figure 3B is higher compared to the others. This is important as the readers may want to compare between different genotypes (double versus single deletion for example). In addition, the authors can consider adding a dashed line at 0.5 to aid the visualization and comparisons.

We have aligned the graphs as recommended. All graphs in Figure 2 now have a y-axis height of 2, the two graphs in Figure 3 now have a y-axis height of 80, all RT-qPCR graphs in Figure 4 have a y-axis height of 1.5, and all graphs in Figure S2 have a y-axis height of 2.

4. “To determine if the Sox17e-13 and Sox17e-231 enhancers promote EHBD formation, we had to first reduce Sox17 dosage to bypass the gallbladder hypoplasia phenotype in Fig. 5A.”

© 2026. Published by The Company of Biologists under the terms of the Creative Commons Attribution License (https://creativecommons.org/licenses/by/4.0/).

This sentence is confusing to read. Perhaps the authors meant to bypass the gallbladder "hyperplasia" phenotype in Fig. 5?

We changed the wording of the introductory sentences for this experiment to improve clarity.

Reviewer 2:

1. Recovery of Sox17 short mRNA expression at E11.5, In Sox17Δe-13/Δe-13 and Sox17Δe-231/Δe-231 embryos, Sox17 short mRNA expression is reduced at E9.5 and E10.5, but appears to recover to wild-type levels by E11.5. In contrast, Sox17Δe-13&e-231/Δe-13&e-231 embryos continue to show reduced expression at E11.5. Additional discussion or explanation regarding the apparent recovery of Sox17 expression in the single-enhancer deletion embryos would strengthen the interpretation of these findings.

The recovery of *Sox17* short mRNA expression in the single enhancer-deletion animals and lack of recovery in dual enhancer deletion animals remains an unanswered quandary. We suggest that the enhancers cooperate to drive *Sox17* expression above two temporal developmental thresholds, potentially activating the SOX17 positive autoregulatory loop (Trinh et al., 2025). We speculate that when only one enhancer is lost, *Sox17* may remain above the threshold required for biliary outgrowth. Indeed, the progenitor cells may be in a 'teetering state' without enough *Sox17* expression to maintain developmental tempo, but just enough to promote outgrowth. Progenitor cells within the dual enhancer deletion may enter the same 'teetering state' but not have enough *Sox17* expression to promote biliary outgrowth.

Our previous paper demonstrated that a SOX17 positive-autoregulatory loop is not required for initial segregation of the ventral pancreas and gallbladder at E9.5 but is required for biliary outgrowth at E11.5 (Trinh et al., 2025). Integrating that finding with the logic above, we speculate that single enhancer-deletion animals express sufficient *Sox17* to activate the SOX17 positive autoregulatory loop and drive *Sox17* to a level compatible with biliary outgrowth. Conversely, the dual enhancer deletion may not have enough *Sox17* to fully activate the positive autoregulatory loop, limiting *Sox17* expression and causing stunted biliary outgrowth.

We have condensed these thoughts and inserted them into the discussion under "Developmental stage-dependent gene-dosage thresholds for the actions of *Sox17* during early ventral pancreato-biliary fate selection."

2. Schematic illustration of the Sox17 gene structure. The manuscript describes two alternative promoters within Sox17, where TSS2-derived short mRNA is associated with endodermal expression and TSS1-derived long mRNA is associated with endothelial expression. However, it is somewhat difficult for readers to fully understand the genomic organization solely from the text. It would be helpful to include a schematic diagram showing the Sox17 gene structure, the positions of TSS1 and TSS2, the structures of the short and long transcripts, and the locations of the analyzed enhancer regions.

Thank you for pointing out the conceptual difficulty. To make our data more approachable, we have added a gene diagram in Figure 2A which shows the two alternate TSS and their mRNA forms, as well as the previously reported SOX17 positive autoregulation. These changes are reflected in the manuscript and figure legend.

3. Quantification of cell populations in Fig. 3. The immunostaining images in Fig. 3 suggest that the morphology and overall size of the ventral pancreato-biliary domain differ among genotypes. Therefore, percentage-based quantification alone may not fully capture the biologically relevant changes in progenitor allocation. In addition, the denominator used for calculating the percentages of SOX17+, PDX1+, and SOX17+/PDX1+ cells should be clearly stated in the figure legend and/or Methods section.

We agree that a percentage-based quantification alone may not fully capture the biological relevant changes in progenitor allocation. Obtaining the type of additional data suggested would require additional experimentation that would exceed the three-month timeframe allowed for revision.

© 2026. Published by The Company of Biologists under the terms of the Creative Commons Attribution License (https://creativecommons.org/licenses/by/4.0/).

We have now included the denominators used to calculate relative percentages within the methods section.

4. Ki67 signals in Supplementary Fig. 3B, In Supplementary Fig. 3B, the Ki67 signal in Sox17Δe-13/Δe-13 embryos appears somewhat stronger than in the other genotypes. The authors should clarify whether this reflects a biological difference or is due to staining or imaging conditions.

We agree that the Ki67 staining signal within the *Sox17*$^{\Delta e\text{-}13/\Delta e\text{-}13}$ is stronger than the other genotypes. To remedy what was an imaging issue on our part, we analyzed adjacent sections and selected a different representative image, which is now in Figure S3B.

5. Opposite phenotypes between single- and double-enhancer deletions. The finding that single-enhancer deletions lead to enlarged gallbladders whereas double-enhancer deletions result in smaller gallbladders is particularly interesting. Additional discussion regarding the possible mechanisms underlying these apparently opposite phenotypes would further strengthen the manuscript.

Our response is similar to Reviewer 2 – Point 1. We have added a paragraph in the discussion under "Developmental stage-dependent gene-dosage thresholds for the actions of *Sox17* during early ventral pancreato-biliary fate selection."

6. Significance of experiments using the Sox17GFPCre/+ background. Because Sox17GFPCre/+ mice already exhibit abnormalities in biliary development, the rationale for using this sensitized background could be explained more explicitly. It would also be helpful to clarify how the compound heterozygous phenotypes extend beyond those observed in Sox17GFPCre/+ animals alone.

We have improved our wording within the results to make it more explicit how the using the *Sox17*^{GFPCre} allele sensitizes development to fluctuations in *Sox17* expression.

We have also added a sentence within the results describing how the compound heterozygous extend beyond the phenotype in *Sox17*^{GFPCre} animals by unmasking the differential sensitivity of the cystic duct and gallbladder to perturbations of *Sox17* expression.

7. Relationship to the concept of shadow enhancers. The manuscript would benefit from a slightly deeper discussion of how Sox17e-13 and Sox17e-231 relate to the classical concept of "shadow enhancers," particularly because deletion of either enhancer alone already produces measurable phenotypes.

We agree and have extended our discussion of shadow enhancers.

8. Conservation of these regulatory regions in species lacking a gallbladder. Since the study demonstrates important roles of Sox17e-13 and Sox17e-231 in gallbladder and extrahepatic bile duct development, it would be interesting to discuss whether these regulatory regions are conserved in animal species lacking a gallbladder, such as rats.

To explore this insightful question, we identified the orthologous Sox17e-13 and Sox17e-231 in another gallbladder-containing species (squirrel, *Spermophilus tridecemlineatus*) and two species that lack a gallbladder (rat, *Rattus norvegicus* ; alpaca, *Vicugna pacos*) using the LiftOver tool within the UCSC genome browser (Figure S8A). Unfortunately, the JASPAR 2020 tracks used to identify and compare FOXA2 and GATA4 motifs in human and mouse are not present in these additional species. However, we performed a sequence alignment of the Sox17e-13 and Sox17e-231 orthologs and found the FOXA2 and GATA4 motifs in mice were present in the species lacking a gallbladder (Figure S8B, S8C). We did not attempt to determine if additional FOXA2 and GATA4 motifs are present in the additional species. The predicted binding affinities of the FOXA2 and GATA4 motifs across species show limited variation and no overt differences between gallbladder-present and gallbladder-absent species (Figure S8D, S8E). This data is shown in Supplemental Figure 8, described in the results, and briefly commented on in the discussion.

© 2026. Published by The Company of Biologists under the terms of the Creative Commons Attribution License (https://creativecommons.org/licenses/by/4.0/).

Reviewer 3:

1) Measuring Sox17 levels in the various mutants: the authors do this by whole-embryo qPCR, which is reasonable given the limited material, but this raises the question of whether the measured Sox17 levels are truly representing the levels in the (presumably small) population of pancreato-biliary progenitors (or their fate-restricted progeny) at E9.5-E11.5. Is Sox17 expressed elsewhere in the embryo at these timepoints, and if so would this change the Sox17 dosages measured in Figure 2D? Perhaps the authors could re-analyze existing whole-embryo scRNA-seq to test this, or use IF or RNA FISH at E9.5.

A similar concern was raised by Reviewer 1 – Point 4. *Sox17* is initially expressed in early development in primitive (extra-embryonic) endoderm and then in definitive endoderm during gastrulation (Nowotschin et al., 2019). Endodermal *Sox17* then becomes gradually restricted to the posterior foregut by E8.5, then to just ventral pancreato-biliary progenitors, before becoming fully restricted to the extrahepatic biliary system by E11.5 (Uemura et al., 2010). *Sox17* is also critical for the expansive vascular development. At E9.5, *Sox17* is expressed within the vascular endothelium where it is critical for the specification of arterial cell fate and will remain expressed in arteries throughout the life of the animal (Corada et al., 2013). In addition, *Sox17* is critical for development of the hemogenic endothelium, a transient subset of vascular cells which produce hematopoietic stem cells, which between E10.5 and E11.5 is primarily restricted to the aorta-gonad-mesonephros region (Clarke et al., 2013).

We do not think that expression of *Sox17* in vasculature would affect the *Sox17* dosages measured in Figure 2. Our prior studies have shown that the TSS1-derived *Sox17* long mRNA form is restricted to the vasculature, whereas the TSS2-derived *Sox17* short mRNA is preferentially expressed in the endoderm, with limited residual expression in the endothelium (Trinh et al., 2022). Moreover, our prior studies showed that the deletion of critical *cis*-regulatory elements within the TSS2 promoter has little effect on vasculature development (Trinh et al., 2025). In total, we do not think that the issue has been definitively settled and hope that future spatial transcriptomics experiments resolve the lineage specificity of *Sox17* mRNA forms and their relative abundances across lineages.

2) Differing dependencies of HPB phenotypes on Sox17 dosage would be easier for readers to visualize/understand if the authors could plot, for any given phenotype, the phenotype as a function of measured Sox17 dosage (% of wild-type), i.e. the levels shown in Figure 2D or Figure 6A. This would be a more intuitive way of visualizing what the different Sox17 dosage thresholds are without having to refer back to previous panels to understand what % dosage each genotype corresponds to.

The summary table below aggregates *Sox17* dosage, type of *Sox17* disruption, embryonic phenotypes, and adult phenotypes for six different allelic combinations. We are hesitant to include a dosage vs. phenotypic graph given the complex temporal nature of our alleles and have included this table in Supplemental Figure 9.

Condition	Δe-13/Δe-13	Δe-231/Δe-231	Δe-13&e-231/Δe-13&e-231	Δe-13/GFPCre	Δe-231/GFPCre	Δe-13&-231/GFPCre
Alleles	*Sox17*$^{\Delta e\text{-}13}$	*Sox17*$^{\Delta e\text{-}231}$	*Sox17*$^{\Delta e\text{-}13\&e\text{-}231}$	*Sox17*$^{\Delta e\text{-}13}$<br>*Sox17*^{GFPCre}	*Sox17*$^{\Delta e\text{-}231}$<br>*Sox17*^{GFPCre}	*Sox17*$^{\Delta e\text{-}13\&e\text{-}231}$<br>*Sox17*^{GFPCre}
Effect on *Sox17* short mRNA form	E8.5: ns<br>E9.5: -31%<br>E10.5 -29%<br>E11.5: ns	E8.5: ns<br>E9.5: -26%<br>E10.5 -17%<br>E11.5: ns	E8.5: ns<br>E9.5: -48%<br>E10.5 -41%<br>E11.5: -40%	E9.5: -43%	E9.5: -49%	E9.5: -61%
Effect on E18.5 Pancreas Size	Decrease	Decrease	Decrease	Not surveyed	Not surveyed	Not surveyed
Effect on Adult Gallbladder Size	Increase	Increase	Decrease	Decrease or Agenesis	Decrease or Agenesis	Agenesis
Additional Phenotypes	N/A	Decreased *Trex17* expression	Decrease in *Trex17* expression; Abnormal gallbladder histology	Gallbladder agenesis with a normal cystic duct	Gallbladder agenesis with a normal cystic duct	Biliary atresia

3) There are a few places where it seems like the dosage-dependencies don't line up, but this may be due to point 2. For example, when considering gallbladder weight in both the enhancer-only deletions (Figure 5B) and sensitized background (Figure 6C), it would appear that Δe-13/GFPCre has a significantly more severe phenotype than Δe-13&e-231/Δe-13&e-231, despite the two genotypes

© 2026. Published by The Company of Biologists under the terms of the Creative Commons Attribution License (https://creativecommons.org/licenses/by/4.0/).

having approximately the same dosage reduction of ~50%. This could be clarified by placing the data on the same dosage % scale as suggested above.

We agree that the compound heterozygous animals have a more severe phenotype than the dual deletion given their adult biliary phenotypes. When comparing the compound heterozygotes to the enhancer deletions, it is difficult to discern how having similar reductions in *Sox17* leads to different phenotypes as the enhancer deletions have temporal effects, whereas the *Sox17*^{GFPCre} allele (functional null) has a constitutive reduction in *Sox17*. This constitutive reduction in *Sox17* may further delay or alter the tempo of transcriptional state transitions within progenitor cells prior to Sox17e-13 or Sox17e-231 activity, which may cause different phenotypes observed despite having similar reductions in *Sox17* dosage.

4) In the authors Figure 7B model, they only rationalize/consider the effects of the enhancer-alone deletions. What about the phenotypes of compound heterozygous deletions (i.e. Figure 6)? How do these fit into the model? This relates to my broader observation that the enhancer-only deletions and the sensitizing background deletions are treated as two distinct experiments, where in reality they should be considered as exploring different parts of the Sox17 dosage-response curve. For example, the authors demonstrate a severe reduction in EHBD Lumen Area in the Δe-13&e-231/GFPCre mutant (~60% Sox17 reduction) - is it a very sharp transition to this phenotype? This could be answered by quantifying EHBD Lumen Area some of the other mutants (i.e. Δe-13/Δe-13, Δe-231/Δe-231, Δe-13&e-231/Δe-13&e-231, GFPCre/+, Δe-13/GFPCre, Δe-231/GFPCre)

The complexity of our findings and likely need for an even deeper analysis led us to treat the enhancer deletions and compound heterozygous analyses as two distinct experiments.

The findings from the compound heterozygotes do not easily fit into a dosage-response curve model. The *Sox17*^{GFPCre} allele causes a constant decrease in *Sox17* expression which is further reduced in a temporal manner by deleting each enhancer. We have added a short description of the alleles used within the study to Supplemental Figure 9C to emphasize their different nature.

The driving reason behind measuring the lumen area of the *Sox17*$^{\Delta e\text{-}13\&e\text{-}231/GFPCre}$ was embryonic lethality. Our goal was to try to connect reductions in *Sox17* dosage to the disease phenotype of biliary atresia, as opposed to simply surveying graded developmental differences. In a future study, we hope to uncover if the EHBDs become smaller in response to *Sox17* reductions in a gradient or threshold-like fashion.

Clarke, R. L., Yzaguirre, A. D., Yashiro-Ohtani, Y., Bondue, A., Blanpain, C., Pear, W. S., Speck, N. A. and Keller, G. (2013) 'The expression of Sox17 identifies and regulates haemogenic endothelium', *Nat Cell Biol* 15(5): 502-10.
Corada, M., Orsenigo, F., Morini, M. F., Pitulescu, M. E., Bhat, G., Nyqvist, D., Breviario, F., Conti, V., Briot, A., Iruela-Arispe, M. L. et al. (2013) 'Sox17 is indispensable for acquisition and maintenance of arterial identity', *Nat Commun* 4: 2609.
Dixon, J. R., Selvaraj, S., Yue, F., Kim, A., Li, Y., Shen, Y., Hu, M., Liu, J. S. and Ren, B. (2012) 'Topological domains in mammalian genomes identified by analysis of chromatin interactions', *Nature* 485(7398): 376-80.
Li, L. C., Qiu, W. L., Zhang, Y. W., Xu, Z. R., Xiao, Y. N., Hou, C., Lamaoqiezhong, Yu, P., Cheng, X. and Xu, C. R. (2018) 'Single-cell transcriptomic analyses reveal distinct dorsal/ventral pancreatic programs', *EMBO Rep* 19(10).
Nowotschin, S., Setty, M., Kuo, Y. Y., Liu, V., Garg, V., Sharma, R., Simon, C. S., Saiz, N., Gardner, R., Boutet, S. C. et al. (2019) 'The emergent landscape of the mouse gut endoderm at single-cell resolution', *Nature* 569(7756): 361-367.
Pan, F. C. and Wright, C. (2011) 'Pancreas organogenesis: from bud to plexus to gland', *Dev Dyn* 240(3): 530-65.
Trinh, L. T., Finnel, R. R., Osipovich, A. B., Musselman, J. R., Sampson, L. L., Wright, C. V. E. and Magnuson, M. A. (2025) 'Positive autoregulation of Sox17 is necessary for gallbladder and extrahepatic bile duct formation', *Development* 152(2).
Trinh, L. T., Osipovich, A. B., Sampson, L., Wong, J., Wright, C. V. E. and Magnuson, M. A. (2022) 'Differential regulation of alternate promoter regions in Sox17 during endodermal and vascular endothelial development', *iScience* 25(9): 104905.

© 2026. Published by The Company of Biologists under the terms of the Creative Commons Attribution License (https://creativecommons.org/licenses/by/4.0/).

Uemura, M., Hara, K., Shitara, H., Ishii, R., Tsunekawa, N., Miura, Y., Kurohmaru, M., Taya, C., Yonekawa, H., Kanai-Azuma, M. et al. (2010) 'Expression and function of mouse Sox17 gene in the specification of gallbladder/bile-duct progenitors during early foregut morphogenesis', *Biochem Biophys Res Commun* 391(1): 357-63.

Second decision letter

MS ID#: dev.205751R1

MS TITLE: Two distributed enhancers of Sox17 additively regulate the developmental fate of bipotential ventral pancreato-biliary progenitor cells

AUTHORS: Ryan R. Finnel, Jessica R. Musselman, Margaret E. McFadden, Isabella M. Silvestri, Jennifer Skelton, Leesa L. Sampson, Anna B. Osipovich, Christopher V. E. Wright and Mark A. Magnuson

Dear Dr Magnuson,

I am happy to tell you that your manuscript has been accepted for publication in Development, pending our standard publication integrity checks.

Reviewer 1

SUMMARY OF THE ADVANCE MADE IN THIS PAPER AND ITS POTENTIAL SIGNIFICANCE TO THE FIELD

The revised manuscript addressed all previous concerns.

Reviewer 2

The authors have adequately addressed all of the reviewers' comments and concerns. I believe that the manuscript is now suitable for publication in Development and recommend its acceptance.

Reviewer 3

The authors have adequately addressed my comments

© 2026. Published by The Company of Biologists under the terms of the Creative Commons Attribution License (https://creativecommons.org/licenses/by/4.0/).
